# Large transcription units unify copy number variants and common fragile sites arising under replication stress

**DOI:** 10.1101/gr.177121.114

**Published:** 2015-02

**Authors:** Thomas E. Wilson, Martin F. Arlt, So Hae Park, Sountharia Rajendran, Michelle Paulsen, Mats Ljungman, Thomas W. Glover

**Affiliations:** 1 Department of Pathology,; 2 Department of Human Genetics,; 3 Department of Radiation Oncology and Translational Oncology Program, University of Michigan, Ann Arbor, Michigan 48109, USA

## Abstract

Copy number variants (CNVs) resulting from genomic deletions and duplications and common fragile sites (CFSs) seen as breaks on metaphase chromosomes are distinct forms of structural chromosome instability precipitated by replication inhibition. Although they share a common induction mechanism, it is not known how CNVs and CFSs are related or why some genomic loci are much more prone to their occurrence. Here we compare large sets of de novo CNVs and CFSs in several experimental cell systems to each other and to overlapping genomic features. We first show that CNV hotpots and CFSs occurred at the same human loci within a given cultured cell line. Bru-seq nascent RNA sequencing further demonstrated that although genomic regions with low CNV frequencies were enriched in transcribed genes, the CNV hotpots that matched CFSs specifically corresponded to the largest active transcription units in both human and mouse cells. Consistently, active transcription units >1 Mb were robust cell-type-specific predictors of induced CNV hotspots and CFS loci. Unlike most transcribed genes, these very large transcription units replicated late and organized deletion and duplication CNVs into their transcribed and flanking regions, respectively, supporting a role for transcription in replication-dependent lesion formation. These results indicate that active large transcription units drive extreme locus- and cell-type-specific genomic instability under replication stress, resulting in both CNVs and CFSs as different manifestations of perturbed replication dynamics.

Structural chromosome alterations account for a large proportion of genomic diversity and cause many human genomic disorders and cancers. In the germline, predominant alterations are kb- to Mb-scale copy number variants (CNVs), including simple deletions and tandem duplications, as well as inversions and more complex intrachromosomal rearrangements with multiple breakpoint junctions (Conrad et al. 2010b; Mills et al. 2011; Liu et al. 2012). Cancer genomes show somatic acquisition of all of these changes in addition to inter-chromosomal rearrangements (Stratton 2011).

CNVs serve as a prototype for understanding many types of gross chromosomal rearrangements. Mechanistically, most CNVs can be divided into those that arise by nonallelic homologous recombination (NAHR) and those that use nonhomologous repair and show, at most, microhomology at junctions (Liu et al. 2012). NAHR-mediated CNVs are recurrent in that multiple de novo events all duplicate or delete the same genomic sequence between large blocks of homology. Although more frequent overall, nonhomologous CNVs are nonrecurrent, meaning that different de novo events rarely represent the same mutation. Regional clustering of nonrecurrent CNVs is nonetheless observed across many patients, which reflects a phenotypic selection bias and also a potential for poorly understood locus-specific influences on CNV formation.

Many observations link nonrecurrent CNV formation to errors of DNA replication. Studies of human pathogenic CNVs have revealed simple deletions and duplications as well as complex events hypothesized to reflect one or more microhomology-mediated template switches from a single stalled or collapsed replication fork (Lee et al. 2007; Hastings et al. 2009). Similar human polymorphic CNVs suggest a generality of this mechanism, which contrasts with the possible formation of CNVs by nonhomologous end joining (NHEJ) (Conrad et al. 2010a). Support for a replication-error origin of nonrecurrent CNVs comes from experimental studies of cultured cells. Both simple and complex CNVs with nonhomologous breakpoint junctions arise sporadically and are induced in normal human cells by partial inhibition of replication with low-doses of aphidicolin (APH), hydroxyurea (HU), or ionizing radiation (IR) (Fig. 1A; Arlt et al. 2009, 2011, 2014). Similar CNVs were also induced in mouse embryonic stem (mES) cells in the presence or absence of XRCC4, a key NHEJ protein, reinforcing the idea that nonrecurrent CNVs are mainly created by replicative repair mechanisms when fork progression is impaired (Arlt et al. 2012).

**Figure 1.**
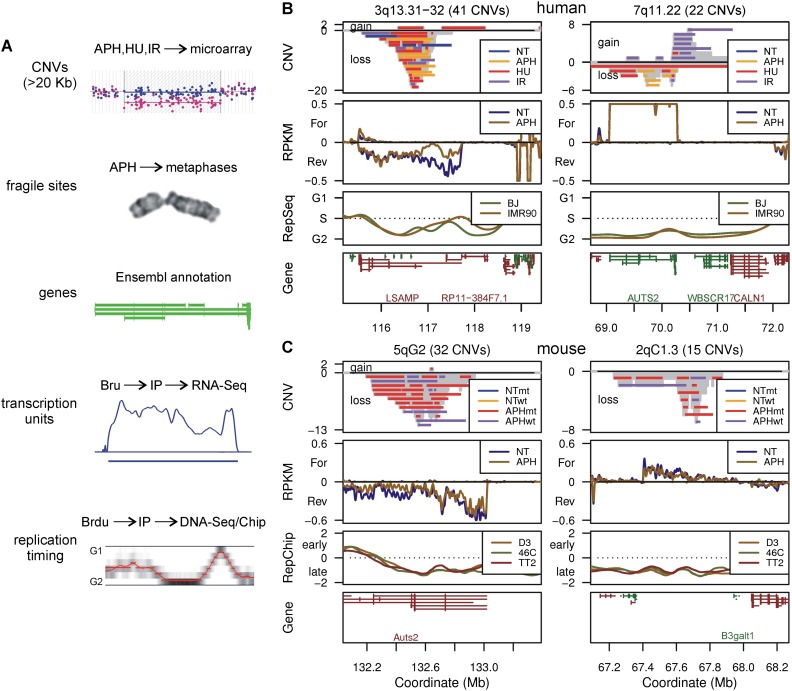
Human and mouse CNV hotspot examples. (*A*) Summary of the genome features compared in this study and their acquisition methods. (*B*,*C*) Profiles of the two most highly clustered de novo CNV regions in human 090 fibroblasts and mES cells, respectively. CNVs are drawn as horizontal bars. The number of CNVs overlapping each genome bin is plotted as a gray histogram: positive CNV counts, duplications/gains; negative counts, deletions/losses. Bru-seq transcription data are plotted as follows: positive RPKM, forward transcription; negative RPKM, reverse transcription. ENCODE Repli-seq data (RepSeq) are plotted as the calculated replication timing, Repli-chip (RepChip) as the log_2_ of the replication timing ratio. Genes are shown as Ensembl transcripts: green lines, forward gene orientations; red lines, reverse orientations. Except for mouse *B3galt1*, labels are suppressed under 500-kb gene lengths. (NT) Untreated; (APH) aphidicolin; (HU) hydroxyurea; (IR) ionizing radiation; (wt) *Xrcc4*^+/+^; (mt) *Xrcc4*^−/−^. See Supplemental Figure S1 for additional profile plots.

Common fragile sites (CFSs) are a distinct phenomenon of structural chromosome instability seen as repeated breaks and gaps in specific regions of metaphase chromosomes (Fig. 1A) (Glover et al. 1984). Like CNVs, CFSs are also induced by low dose-replication stress insufficient to stop cell division. CFSs are thought to be cytogenetic markers of loci prone to structural alterations, an idea supported by CFS rearrangements in multiple tumor types (Arlt et al. 2006; Bignell et al. 2010). In addition, many of the spontaneous and replication stress-induced CNVs seen in experimental cell systems overlap in specific genomic regions called hotspots, some of which correspond to CFSs (Durkin et al. 2008; Arlt et al. 2011). However, other CFSs were devoid of CNVs, making the nature of the CNV-CFS relationship uncertain.

The potential link between CNVs and CFSs is informative due to other described CFS properties. CFSs often occur within large genes (Smith et al. 2006). They are also known to replicate late, to be driven to extremes of late replication under replication stress, and to show reduced dormant origin usage in response to such stress (Le Beau et al. 1998; Letessier et al. 2011). CFSs further possess low-complexity A/T-rich sequences prone to forming non-B DNA structures that interfere with replication (Zlotorynski et al. 2003; Walsh et al. 2013). Some combination of these properties appears to lead CFSs to a state of underreplication at metaphase, which must be resolved to breaks to prevent the formation of anaphase bridges (Chan et al. 2009; Naim et al. 2013; Ying et al. 2013). However, the relationships between these phenomena and how they relate to CNV formation remain unclear.

Here we exploit our large sets of experimentally induced CNVs to characterize the genomic features contributing to CNV and CFS formation and clustering. Head-to-head comparisons establish that CNV hotspots and CFSs are the same loci in a given cell line. This correspondence is explained by a strong contribution of active transcription to formation of CNVs genome-wide and a specific association of CNV hotspots and CFSs with the largest active transcription units (TUs). Results from cell lines with differential gene isoform expression further establish TU length as a robust cell-type-specific predictor of locus instability under replication stress. Finally, TU length correlates with replication timing in a manner that confers a regional organization of CNV positions. A unified model is suggested in which large TUs drive extreme locus instability due to a high susceptibility to fork failure, especially double-fork failure, combined with an impaired ability to silently resolve these lesions.

## Results

### Induced CNV cluster regions and hotspots

We combined all previously reported de novo CNVs from our in vitro cell systems into (1) a human set with 360 CNVs from untreated (NT) and APH-, HU- and IR-treated 090 TERT-immortalized fibroblasts and (2) a mouse set with 377 CNVs from NT and APH-treated *Xrcc4*^+/+^ and *Xrcc4*^−/−^ mES cells. The median size of the human and mouse CNVs was 186 kb (1 kb to 80 Mb) and 63 kb (8 kb to 26 Mb), respectively (Supplemental Table S1). Forty-eight human and 41 mouse breakpoint junctions in these CNVs have been sequenced and all showed microhomologies, blunt ends, or small insertions (Arlt et al. 2009, 2011, 2012, 2014). These large sizes and junction properties identify the experimental CNVs examined here as being most similar to the nonrecurrent class of human pathogenic CNVs.

CNV clustering was assessed using simulation analysis (see Supplemental Methods). Here we define a “cluster” as a location in the genome where we observed two or more overlapping or closely adjacent CNVs. A “hotspot” is a cluster with more CNVs than could be accounted for by random chance. A “singleton” is an isolated CNV that did not cluster with any others. Finally, “region” is a generic term that refers to any location where we observed one or more CNVs, which thus includes both clusters and singletons. Overlapping or adjacent CNVs separated by no more than 750 kb were collapsed into a total of 196 human and 189 mouse CNV regions containing from 1 to 41 CNVs (Supplemental Table S2). During this process, a small subset of 25 (6.9%) and 8 (2.1%) exceptionally large CNVs >2.5 Mb were omitted from the human and mouse sets, respectively, to prevent genomic spans on the scale of chromosome arms from being uninformatively nominated as CNV hotspots. Observing even one CNV region containing five or more CNVs was a significant deviation from a random distribution (*P* < 0.017 and *P* < 0.011 for human and mouse, respectively). In addition, simulation iterations rarely showed the large number of regions actually observed with two to four CNVs (Supplemental Table S2). Moreover, because CNV sets were sparsely sampled relative to the size of the genome, even singleton CNVs might represent nonrandom loci. Accordingly, we used all CNV regions in analyses below, with stratification into confirmed hotspots (≥5 CNVs), nonhotspot clusters (2–4 CNVs), and singletons.

The two most highly clustered human and mouse CNV hotspots are depicted in Figure 1, B and C. All CNV regions are depicted in Supplemental Figure S1, G and H and tabulated in Supplemental Table S3A. All CNV endpoints in cluster regions were distinct so that CNV hotspots represented nonrecurrent breakpoint junctions spread over Mb-scale genomic spans. Many regions included CNVs from different inducing agents or *Xrcc4* genotypes, reinforcing the interpretation that all CNVs represent the same formation mechanism.

### CNV hotspots correspond to CFSs

**Figure 2.**
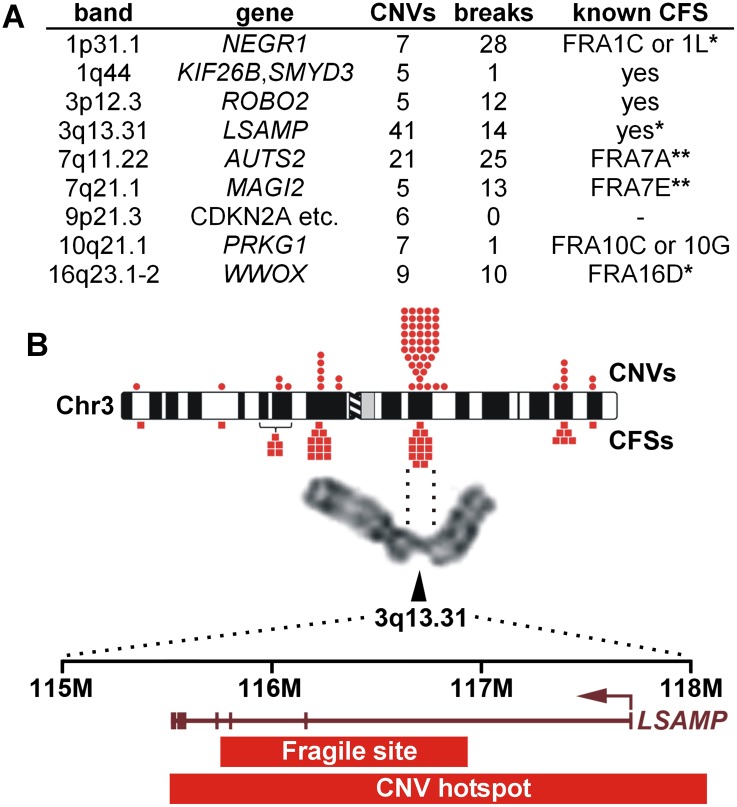
CNV hotspots correspond to CFSs. (*A*) Counts of 090 CNVs from 223 cell clones and CFS breaks from 100 metaphases at the nine 090 CNV hotspots. Correspondence to known CFSs is indicated: (*) CFS location previously characterized by FISH; (**) CFS location characterized by FISH in Figure 5; (–) no known CFS. (*B*) Chr 3 and the 3q13.31/*LSAMP* hotspot/CFS. Symbols *above* and *below* the ideogram denote 090 CNVs and CFS breaks, respectively. CFSs are marked in the *middle* of the corresponding band, or with a bracket indicating multiple possible source bands. 3q13.31 fragile site boundaries are from Le Tallec et al. (2011). See Supplemental Figure S2 and Supplemental Table S4 for complete CFS-CNV data.

We previously noted a partial correspondence of replication-stress-induced CNVs and known CFSs (Arlt et al. 2011) but were uncertain of the extent of this connection given that fibroblasts show cell-type-specific CFS patterns (Murano et al. 1989; Le Tallec et al. 2011). We therefore scored APH-induced metaphase chromosome breakage in our human 090 fibroblast cell line (Supplemental Fig. S2; Supplemental Table S4) and observed CFS breaks at eight of its nine CNV hotspots (Fig. 2A). The exception at 9p21.3 was unusual in that its status as a hotspot was driven by four IR-induced duplications out of six CNVs (see more below). Three of the CFSs at 090 CNV hotspots (FRA16D, FRA3q13, FRA1C/L) have been previously characterized by FISH so that their precise locations are known, all of which coincided well with our hotspot regions (e.g., Fig. 2B; Krummel et al. 2000; Le Tallec et al. 2011). These results suggest that CNV hotspots and CFSs are the same loci and represent different manifestations of the same source lesion. This association with CNVs appeared to extend to nearly all 090 CFS breaks (Supplemental Fig. S2; Supplemental Table S4), although assignments at other sites were hindered by the lower resolution of CFSs on banded chromosomes as compared to CNV localization by microarray. We therefore exploit below the higher kb-scale resolution afforded by CNVs to systematically explore the basis of unstable CNV/CFS loci.

### CNV regions are enriched in large genes

**Figure 3.**
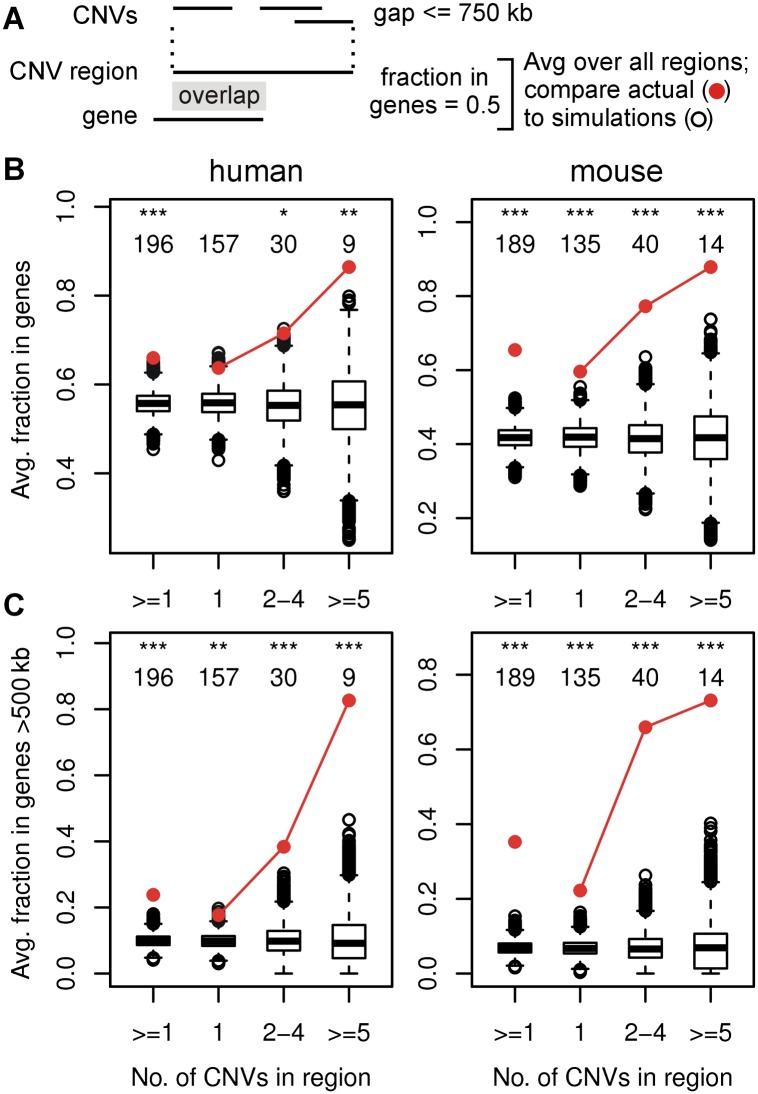
CNV hotspots are enriched in large genes. (*A*) Methods used to merge CNVs into CNV regions and assess overlap with genome features. (*B*,*C*) Enrichment plots for the fraction of CNV regions in genes and genes >500 kb, respectively, for human 090 fibroblasts (*left* panels) and mES cells (*right* panels). Red circles show the actual average values for the indicated CNV region groups. Box and whisker plots show the distribution of averages over all simulation iterations. The number of CNV regions in each group and significant differences between the actual value and iteration distributions are indicated: (*) *P* < 0.01; (**) *P* < 0.001; (***) *P* < 0.0001. See Supplemental Figure S3 for additional enrichment plots.

To quantify enrichment for various genomic features in CNV regions, we again used simulation analysis (summarized in Supplemental Table S5). Importantly, any feature considered to explain CNV and CFS clusters must account for their large and regional nature. Consistently, CNV hotspots did not show enrichment for either G/C content, CpG islands, or small repetitive sequence elements (Supplemental Figs. S3B–D, S3I–K). In contrast, CNV regions often corresponded to some of the largest human and mouse genes (Fig. 1B,C; Supplemental Table S3B). When we compared all 090 fibroblast CNV regions to the Ensembl annotation (Flicek et al. 2014), the actual regions were not significantly more likely to overlap a gene than randomly permuted regions (*P* = 0.17) (Supplemental Fig. S3E). CNV regions in mES cells did show modest gene enrichment (*P* = 0.00022) (Supplemental Fig. S3L). More revealing was that gene enrichment became significant for both 090 and mES cells when scored as the fraction of the span of the CNV regions that overlapped genes (“fraction in genes,” *P* = 7.5 × 10^−5^ and 1.6 × 10^−15^, respectively) (Fig. 3A,B), indicating that CNV regions and genes did not just cross at their edges—they had a high degree of overlap. Finally, the length of the longest gene overlapped by CNV hotspots was significant (*P* = 1.9 × 10^−5^ and 1.6 × 10^−6^) (Supplemental Fig. S3G,N), leading to correlations of CNV clustering with gene size (r = 0.41 and 0.42) and a clear enrichment for the overlap with genes >500 kb (*P* = 2.2 × 10^−13^ and 3.2 × 10^−57^) (Fig. 3C). In total, like CFSs (Smith et al. 2006), large genes are a feature of many spontaneous and induced CNV regions, especially hotspots.

### CNV hotspots correspond to active large transcription units

One hypothesis to explain gene enrichment in CNV regions is that those genes were actively transcribed in our cell lines (Helmrich et al. 2011). To directly relate unstable loci to transcription, we used Bru-seq, a technique in which bromouridine is used to label and capture RNA molecules engaged in synthesis (Fig. 1A; Paulsen et al. 2013a). High-throughput sequencing of immunoprecipitated nascent RNA reveals the complete set of active transcription units (TUs), operationally defined as contiguous genomic DNA spans undergoing transcription in the cell type under study and typically corresponding to the longest expressed isoform of a gene (see Supplemental Methods). Notably, TUs detected by Bru-seq can correspond to unannotated and unstable transcripts as well as extragenic transcription.

Bru-seq was performed on human 090 fibroblasts and mES cells in the presence and absence of low-dose APH. NT and APH samples showed correlation of gene expression over a wide range of transcription intensities, validating their reliability (Supplemental Fig. S4A,B). TUs called from the merged samples are tabulated in Supplemental Table S3C. Strikingly, the most highly clustered CNV regions were principally contained within active large TUs > 500 kb, including *AUTS2*/*Auts2* and other orthologous sites in human and mouse cells (Fig. 1B,C). Moreover, the TUs in the CNV regions at human 3q13.31 and mouse 2qC1.3 predicted the existence of large transcript isoforms not present in the conservative RefSeq annotation. The human 3q13.31 TU extended the 5′ end of the *LSAMP* gene by a remarkable 1.6 Mb, while the mouse 2qC1.3 TU corresponded to an exceptionally long 0.6-Mb isoform of the small annotated gene *B3galt1*.

**Figure 4.**
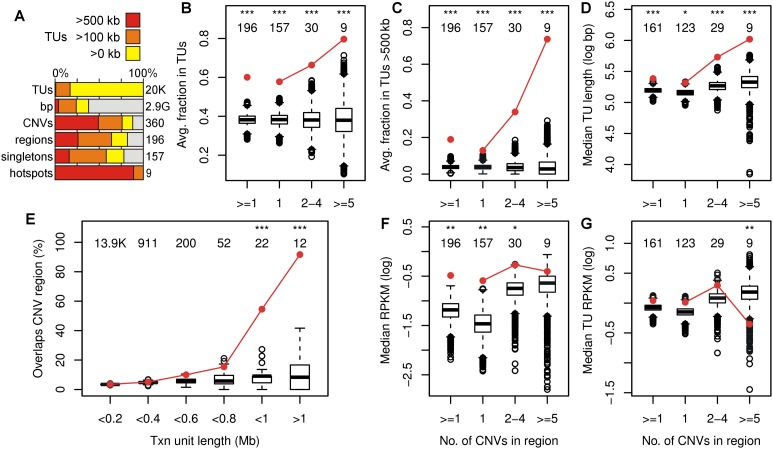
CNV hotspots correspond to active large transcription units. (*A*) Rows represent the total number of TUs, bp, CNVs, CNV regions, singleton CNVs, and hotspots in the mappable genome for human 090 fibroblasts. Colors indicate the percentage that overlapped TUs > 500 kb, > 100 kb, or any length. (*B*–*D*) 090 CNV region enrichment plots for the fraction in Bru-seq TUs, fraction in TUs > 500 kb, and length of the longest overlapped TU, respectively, similar to Figure 3B. (*E*) 090 TU enrichment plot for the percentage of TUs that overlapped one or more CNVs. (*F*,*G*) 090 CNV region enrichment plots for the region’s Bru-seq RPKM and RPKM of the most highly expressed and overlapping TU, respectively. See Supplemental Figures S5 and S6 for mES cell and additional enrichment plots.

In total, the 156 human 090 fibroblast TUs > 500 kb in length accounted for just 0.8% of the 19,803 total TUs and 3.8% of the genome but matched the locations of 175 of 360 individual CNVs (49%), 50 of 196 CNV regions (26%), and eight of nine CNV hotspots with ≥5 CNVs (89%) (Fig. 4A). The 3284 TUs > 100 kb in length accounted for 24% of the genome but matched 274 (76%), 126 (64%), and nine (100%) of the CNVs, CNV regions, and hotspots, respectively. Similar patterns were observed for mES cells (Supplemental Fig. S5A). Unlike CNV assessments, mES cell Bru-seq measurements could only be made after passage without feeder cells, which may influence mES cell transcription. We therefore focus further discussion on 090 fibroblasts, although all conclusions are supported by mES cell data in the Supplemental Material (Supplemental Fig. S5; Supplemental Table S5; and others below).

Simulation analysis confirmed that all 090 CNV region groups, whether containing 1, 2–4, or ≥5 CNVs, were significantly enriched for overlap with active TUs (*P* = 2.2 × 10^−9^, 4.5 × 10^−7^, and 2.3 × 10^−6^, respectively) (Fig. 4B; Supplemental Table S5). Moreover, CNV regions in all three cluster groups were highly enriched for the transcribed portions of genes (*P* = 3.9 × 10^−15^) but depleted for nontranscribed portions (*P* = 9.3 × 10^−5^) (Supplemental Fig. S6A,B). Thus, it appeared to be active transcription, or a property closely associated with transcription, that led to CNV formation in some genes.

Although significant for all groups, the overlap of 090 CNV regions with large TUs > 500 kb was notably greater for hotspots (*P* = 5.0 × 10^−11^, 1.2 × 10^−15^, and ∼0 for 1, 2–4, or ≥5 CNVs, respectively) (Fig. 4C), leading to a correlation of clustering intensity with this parameter (r = 0.42) and with the longest overlapping TU (r = 0.46) (Fig. 4D). Thus, large TU size increased the likelihood of CNVs occurring repeatedly within a locus. This relationship was also forcefully demonstrated by simulation analysis of randomly permuted TUs (Supplemental Table S5), which revealed a striking increase in the correspondence to overlapping CNVs as TU length increased past ∼800 kb (Fig. 4E). Notably, 11 of the 12 TUs > 1 Mb (i.e., 92% of the largest 0.1% of active TUs) matched one or more 090 CNVs (P∼0).

Transcription intensity is a distinct property that might influence CNV formation. We did observe a small enhancement of 090 Bru-seq signal, expressed in reads per kb per million reads (RPKM) units, within CNV regions (*P* = 0.00045) (Fig. 4F). However, this result compares CNV regions, which preferentially overlap active TUs, to the genome at large, most of which is not transcribed (Supplemental Fig. S4D,E). More importantly, we found that 090 CNV regions were not unusual with respect to the highest RPKM of their overlapping TUs (*P* = 0.05) (Fig. 4G). In fact, hotspots showed a small tendency toward lower TU RPKM (*P* = 0.00045), including many of the most highly clustered CNV regions (Fig. 1). Similar conclusions could be reached from TU simulations comparing TU length to RPKM (Supplemental Fig. S6C,D).

### Exceptions to CNVs at large transcription units

There were exceptions to the relationship between 090 CNVs and active large TUs, most notably the 9p21.3 hotspot that was not a CFS. The core five CNVs in this region overlapped transcribed genes *MIR31HG*, *MTAP*, *CDKN2A* (also known as *p16*), *CDKN2B*, and *CDKN2B-AS1* (Supplemental Fig. S1A). Although these TUs were 667 kb in total, they were distinct and did not conform to the single large TUs at other hotspots. Conversely, the 1.0-Mb TU at 2q21.2, gene *NCKAP5*, appeared at risk but matched no 090 CNVs (Supplemental Fig. S1B). This exception might reflect insufficient CNV data or locus-specific modifiers of the CNV-transcription relationship. As CNV regions became less intensely clustered, there were more variations, with singleton CNVs sometimes overlapping large TUs, multiple shorter TUs, single shorter TUs, or none at all (e.g., Supplemental Fig. S1C–F). Overall, 78% of singleton CNVs overlapped an active TU (Supplemental Table S6), with 67% of singletons having one or both of their ends fall within TUs (*P* = 5.4 × 10^−5^) (Supplemental Fig. S6E).

### Validation of the large transcription unit predictor for CNVs and CFSs

To determine whether the observations above establish a predictive model for CNV and CFS formation under replication stress, we performed a prospective study of a new cell line UMHF1 (HF1) for which we have reported detailed Bru-seq descriptions (Paulsen et al. 2013b). Like 090, HF1 is a TERT-immortalized normal human fibroblast line. Nevertheless, Bru-seq revealed differences in their transcription profiles (Supplemental Fig. S4C), most importantly in their >1-Mb active TUs (Table 1). Some were “on/off” differences, while others entailed one line expressing a much longer isoform of a gene. Working from these differences, we predicted that HF1 would fail to show CNVs at the two most intense 090 hotspots at *LSAMP* (3q13.31), which HF1 did not express, and *AUTS2* (7q11.22), for which HF1 expressed a smaller 0.1-Mb isoform. Conversely, we predicted that HF1 would show CNVs at its much longer 1.6-Mb *DAB1* isoform (1p32.1).

**Table 1.**
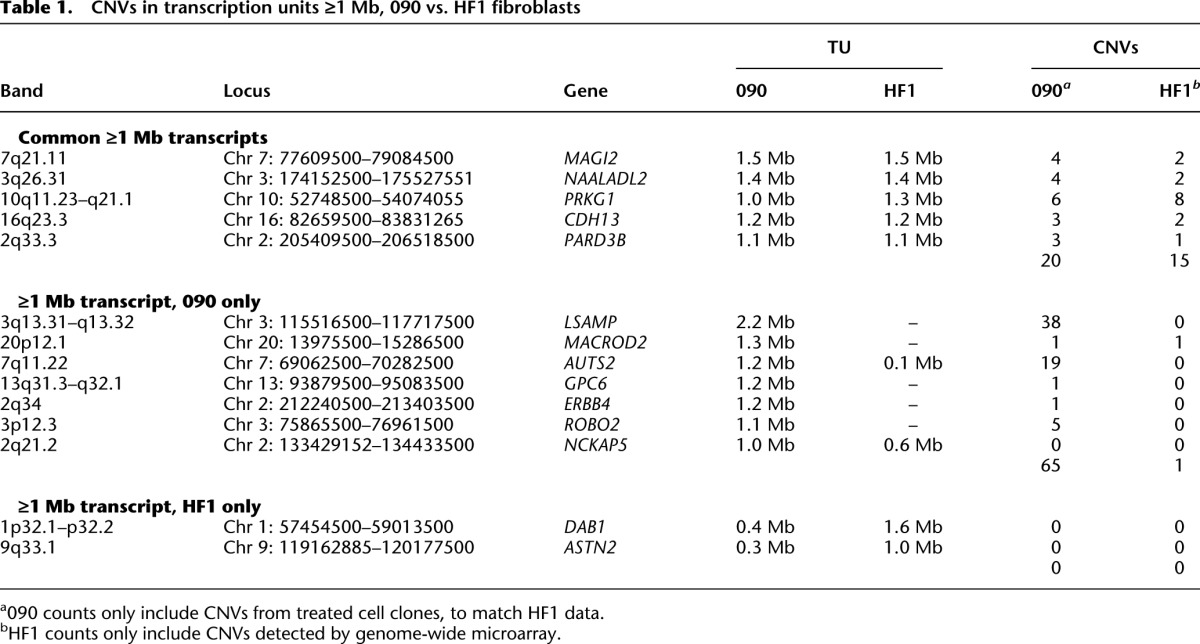
CNVs in transcription units ≥1 Mb, 090 vs. HF1 fibroblasts

**Figure 5.**
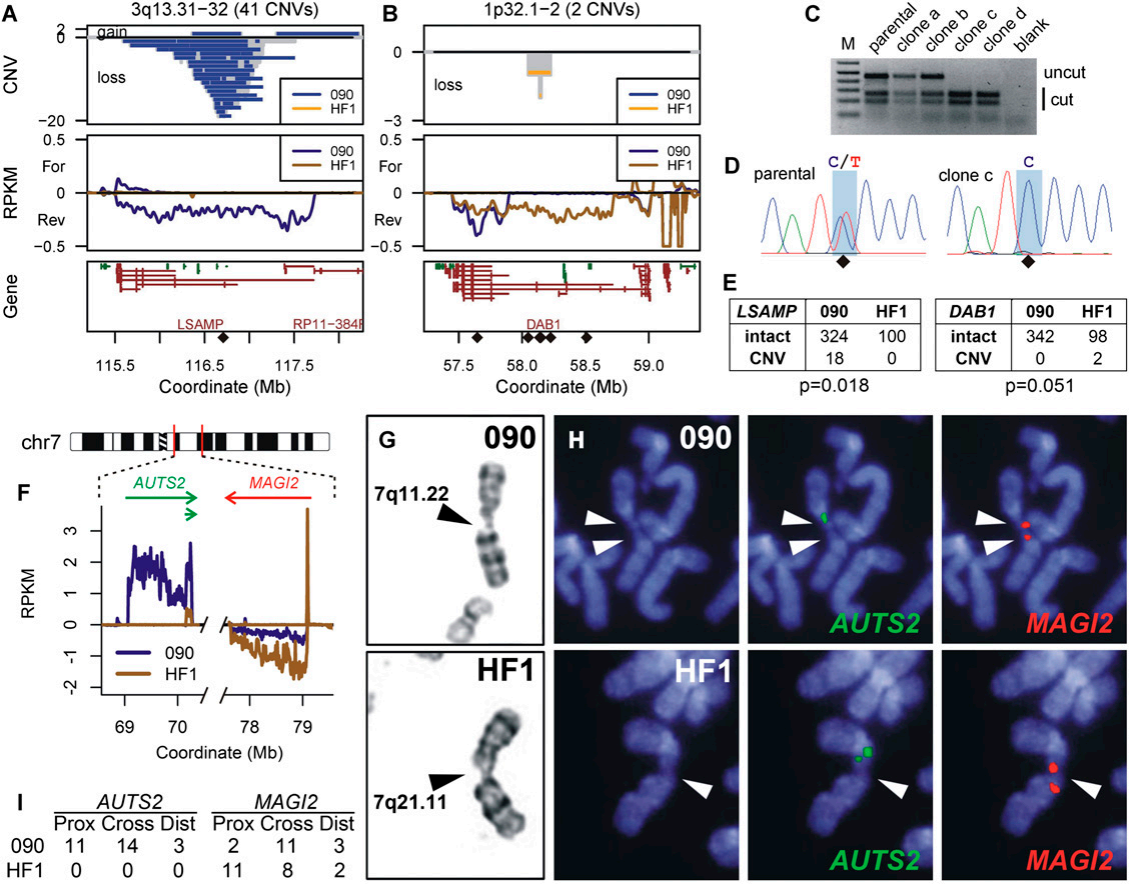
Cell-type-specific prediction of unstable loci at active large transcription units. (*A*,*B*) Chromosome region profiles, similar to Figure 1, B and C, for genes *LSAMP* and *DAB1*, respectively, with CNVs colored by their detection in either 090 or HF1 fibroblasts. Diamonds mark the positions of SNP RFLPs interrogated in HF1. (*C*) BccI digestion of SNP rs79114629 PCR products for HF1 parental cells and two APH-treated clones lacking (a and b) and containing (c and d) a deletion CNV. (*D*) Sequence analysis of clone c demonstrating LOH at SNP rs79114629. (*E*) Allele counts for *LSAMP* and *DAB1*, where 090 counts only include CNVs from treated clones detectable by the HF1 RFLP analysis. (*F*) Portions of 090 and HF1 Bru-seq transcription data relevant to CFS analysis at 7q11.22–q21.11, showing differential transcription of *AUTS2*. (*G*) Examples of G-banded chromosomes demonstrating breaks at 7q11.22 in 090 (*top*) and 7q21.11 in HF1 (*bottom*). (*H*) Representative FISH on DAPI stained chromosomes using probes to *AUTS2* (green, *middle*) and *MAGI2* (red, *right*). 090 shows breaks at both loci in a single chromosome (*top*), while HF1 shows a break at *MAGI2* (*bottom*). (*I*) Summary of 090 and HF1 CFS breaks with respect to *AUTS2* and *MAGI2* FISH probes.

We performed SNP microarray analysis on 14 HF1 cell clones treated with 0.4 µM APH (Table 1; Supplemental Table S8). We detected 15 de novo CNVs in the five genes for which HF1 expressed a matching long transcript to 090, compared to 20 CNVs in 171 treated 090 cell clones. In contrast, we detected only one CNV in the seven genes for which HF1 had a much shorter or no TU, compared to 65 CNVs in 090 (*P* = 1.4 × 10^−7^ by Fisher’s exact test) (Table 1). To increase statistical power at specific loci, we scored an additional 36 HF1 cell clones for loss-of-heterozygosity (LOH) at one SNP in the middle of the 090 *LSAMP* CNV hotspot and six SNPs in *DAB1* (Fig. 5A–D). Consistent with predictions, we detected no LOH events indicative of deletion CNVs in the untranscribed HF1 *LSAMP* gene, for a total of 0/100 HF1 *LSAMP* alleles with CNVs (*P* = 0.018 in comparison to 090) (Fig. 5A,E). In contrast, we detected two HF1 CNVs at *DAB1* (*P* = 0.051) (Fig. 5B,E). *DAB1* clearly demonstrated the high CNV risk at active large TUs, since using only Bru-seq profiles we correctly predicted a site where multiple CNVs would occur in only 50 HF1 cell clones.

We also used HF1 to test whether large active TUs could precisely predict CFS loci. We first scored a more limited set of APH-treated HF1 metaphases and observed that this cell line did manifest breaks and gaps at many of the CNV hotspots it held in common with 090, as well as at its own CNV clusters (Supplemental Fig. S2; Supplemental Table S4). More importantly, 090 fibroblasts had CNV hotspots and large TUs at both *AUTS2* (7q11.2) and the nearby *MAGI2* (7q21.11) genes, while HF1 showed CNVs and a large TU only at *MAGI2* (Table 1; Fig. 5F). We therefore predicted that both of these 7q loci would be fragile in 090, but that only *MAGI2* would be fragile in HF1. This prediction appeared to be confirmed on G-banded chromosomes (e.g., Fig. 5G), but these metaphases lacked the resolution required to define the precise band at which all 7q11–q21 breaks occurred. We therefore performed metaphase FISH using BAC probes specific to *AUTS2* and *MAGI2* (e.g., Fig. 5H). Because CFS loci are larger than BAC probes, a CFS will have FISH signals immediately adjacent to, or split by, different breaks. 090 demonstrated this CFS FISH pattern at both *AUTS2* (28 breaks in 188 Chr 7 homologs) and *MAGI2* (16 breaks in 188 Chr 7 homologs). Consistent with predictions, HF1 only demonstrated a CFS at *MAGI2* (21 breaks in 300 Chr 7 homologs) (Fig. 5I).

### CNV hotspots/large transcription units replicate late

We next asked whether replication timing in the cell cycle could refine our understanding of the relationship between CFSs/CNV hotspots and transcription. We used publicly available ENCODE Repli-seq data for human fibroblast lines IMR-90 and BJ (Hansen et al. 2010) and Repli-chip data for 46C, D3, and TT2 mES cells (Fig. 1A; Hiratani et al. 2008). Use of these data was justified because replication timing is much less variable than transcription between cell lines of the same type, including at CNV hotspots (Supplemental Fig. S1G,H), with excellent correlation between paired fibroblast or lymphoblastoid lines (r = 0.937 and 0.977, respectively) (Supplemental Fig. S8A–C). Also, GM12878 lymphoblastoid cell Bru-seq data obtained here and publicly available IMR-90 GRO-seq transcription data (Core et al. 2008) provided confirmation of observations below using explicitly matched replication and transcription data.

**Figure 6.**
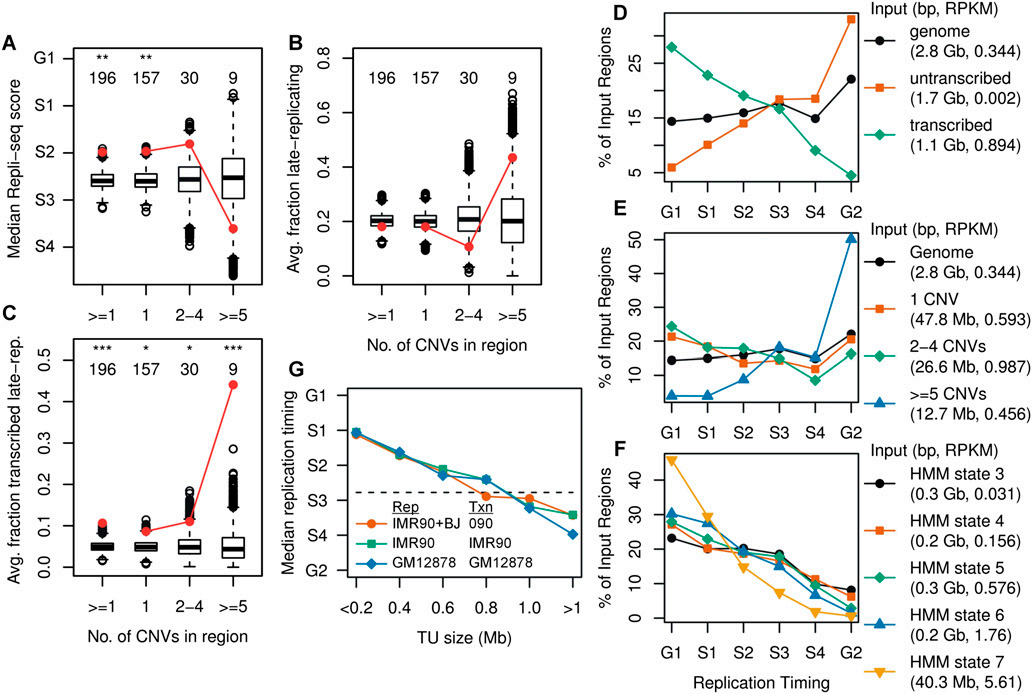
CNV clustering extent stratifies by replication timing. (*A*–*C*) Human 090 fibroblast CNV region enrichment plots for the average replication timing in IMR-90 + BJ Repli-seq data, fraction in late-replicating segments, and fraction in the transcribed portions of late-replicating segments, respectively. (*D*) Distribution of replication timing for the entire genome as well as the transcribed and untranscribed portions of the genome, based on 090 Bru-seq and IMR-90 + BJ Repli-seq data. The legend indicates the aggregate size and Bru-seq RPKM of all input genome regions contributing to each trace. Each trace sums to 100% of its input regions. (*E*,*F*) Replication timing plots for CNV region groups and the transcribed portion of the genome stratified by transcription intensity, respectively. (*G*) Median replication timing for all TUs stratified into 200-kb size bins for different paired Repli-seq (Rep) and Bru-seq/GRO-seq (Txn) samples. A horizontal line indicates the IMR-90 + BJ genome median. See Supplemental Figures S7 and S8 for mES cell and additional enrichment plots.

Interestingly, CNV hotspots, and thus CFSs, did not show significant enrichment for late replication timing scores for either 090 fibroblasts or mES cells (*P* = 0.08 and 0.18, respectively) (Fig. 6A; Supplemental Fig. S7A). Similarly, 090 hotspots did not show enrichment for the fraction of hotspots in late-replicating genomic segments with Repli-seq scores from mid-S4 to G2, as defined by Hansen et al. (2010) (*P* = 0.05) (Fig. 6B), although mES cell hotspots did show enrichment for late-replicating segments with Repli-chip ratios below −0.8, as defined by Hiratani et al. (2008) (*P* = 3.3 × 10^−6^) (Supplemental Fig. S7B). We did notice a trend of 090 hotspots toward late replication and considered that this did not achieve significance because hotspots were 0.4% of the genome whereas ∼20% of the genome is expected to be late-replicating. Indeed, the majority of even very large late-replicating regions did not show any 090 or HF1 CNVs (Supplemental Table S3D), suggesting that late replication alone is insufficient for CNV formation. We surmised that the exceptional replicative attribute of CNV hotspots/CFSs was that they were both late-replicating and transcribed. We indeed observed a strong enrichment for this combination of properties in both 090 fibroblasts and mES cells (*P* = 1.2 × 10^−17^ and 1.1 × 10^−63^, respectively) (Fig, 6C; Supplemental Fig. S7C).

To explore this relationship further, we stratified the genome into all replication and transcription state combinations. We confirmed observations that active transcription is a dominant factor associated with early replication in multiple cell types (Fig. 6D; Supplemental Figs. S7D, S8D,E; Karnani et al. 2010; Fraser 2013). In marked contrast, CNV hotspots/CFSs, even though transcribed, consistently replicated late (Fig. 6E; Supplemental Fig. S7E). Importantly, this difference did not depend on selection for CNV regions per se. Instead, there was a clear relationship among all TUs across multiple cell types that replication occurred later as TU size increased (Fig. 6G; Supplemental Fig. S8H,I; Lawrence et al. 2013). Further analysis revealed an explanation for the observations above that CNV hotspots are not associated with high levels of transcription. Specifically, increasing transcription intensity was associated with earlier replication in multiple cell types, with the most highly expressed TUs being almost exclusively early-replicating (Figs. 6F; Supplemental Figs. S7F, S8F,G). It was thus consistent that the late-replicating TUs at CNV hotspots were unusually long but not intensely transcribed.

We used FISH to verify late replication of hotspot genes *LSAMP* and *AUTS2* in human 090 and HF1 fibroblasts by comparing the fraction of chromosomes with a duplicated hotspot probe to the early-replicating control gene *C16orf45* (Supplemental Fig. S9A). Each hotspot gene replicated later than control in both 090 and HF1 cells (Supplemental Fig. S9B,C), despite the fact that *LSAMP* and *AUTS2* only showed active large TUs and CNV hotspots in 090 (Table 1). Large TUs were thus not causing late replication of their genes, consistent with the late replication of the untranscribed genome (Fig. 6D). These conclusions were confirmed by comparing IMR-90 fibroblast and GM12878 lymphoblastoid cell lines at all Ensembl-annotated genes >1 Mb (Supplemental Fig. S9D–G). In fact, the presence of large active TUs was associated with slightly earlier replication (Supplemental Fig. S9H), although still much later than most small expressed genes.

### Large transcription units organize replication timing and CNV formation

**Figure 7.**
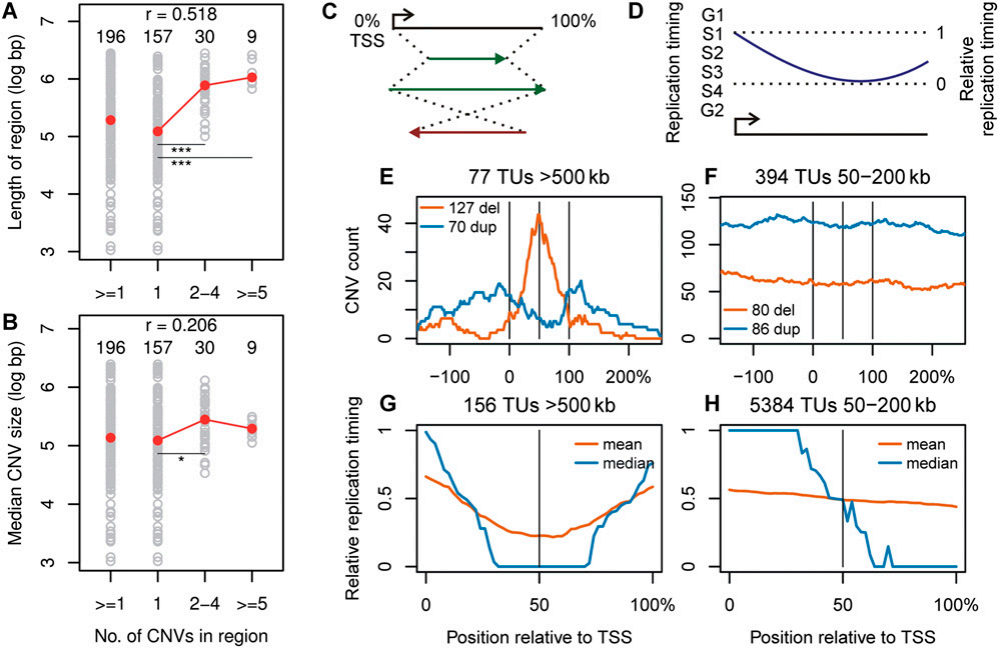
Transcription unit replication dynamics shape CNV size, location, and type. (*A*,*B*) Correlation plots of the number of 090 fibroblast CNVs contained in CNV regions vs. the length of the regions and median size of CNVs in the regions, respectively. Individual regions are plotted as gray circles. Red circles are the group medians. The number of regions in each group, Spearman correlation coefficients (*r*), and significant differences between groups are indicated: (*) *P* < 0.01; (**) *P* < 0.001; (***) *P* < 0.0001. (*C*) Coordinate transformation used to align TUs according to their endpoints. (*D*) Transformation of replication timing data from absolute values to ones relative to the minimum and maximum within a TU. (*E*,*F*) Sum of 090 CNV counts within and flanking TUs > 500 kb and between 50 and 200 kb, respectively. The *y*-axis represents the number of CNVs crossing each plotted position. (*G*,*H*) Mean and median IMR-90 + BJ relative replication timing by position within 090 TUs > 500 kb and between 50 and 200 kb, respectively. See Supplemental Figures S10 and S11 for mES cell and additional alignment plots.

We noted that the number of CNVs in different CNV regions correlated with the region sizes (r = 0.52 and 0.62 for 090 and mES cells, respectively) (Fig. 7A; Supplemental Fig. S10A) and to a lesser extent with the sizes of their individual CNVs (r = 0.21 and 0.33) (Fig. 7B; Supplemental Fig. S10B). These observations suggested that the large TUs at CNV hotspots were driving CNV formation within their boundaries. To explore this idea, we transformed all active TUs > 500 kb into a coordinate system corresponding to the percentage distance along each unit, setting the transcription start site (TSS) to 0% (Fig. 7C). Doing so strikingly revealed not only that deletion CNV spans had a nearly normal distribution within and centered on their TUs, but also that duplication CNVs accumulated on the TU flanks (Fig. 7E; Supplemental Figs. S10C, S11A,B). Selective CNV placement was not observed for smaller 50- to 200-kb TUs (Fig. 7F; Supplemental Fig. S10D). We hypothesized that this distinction resulted from the relative distribution of replication timing within the body of TUs (Fig. 7D) and observed that the middle of large TUs tended to replicate later than their ends (Fig. 7G,H; Supplemental Figs. S10E,F, S11C–E). Thus, the middle of many large genes could be inferred to be dependent on replication forks proceeding inward from the 3′ as well as the 5′ ends. This effect did not account entirely for the late absolute replication timing of large TUs, since even TSSs replicated later as TU size increased (Supplemental Fig. S11F–H).

## Discussion

**Figure 8.**
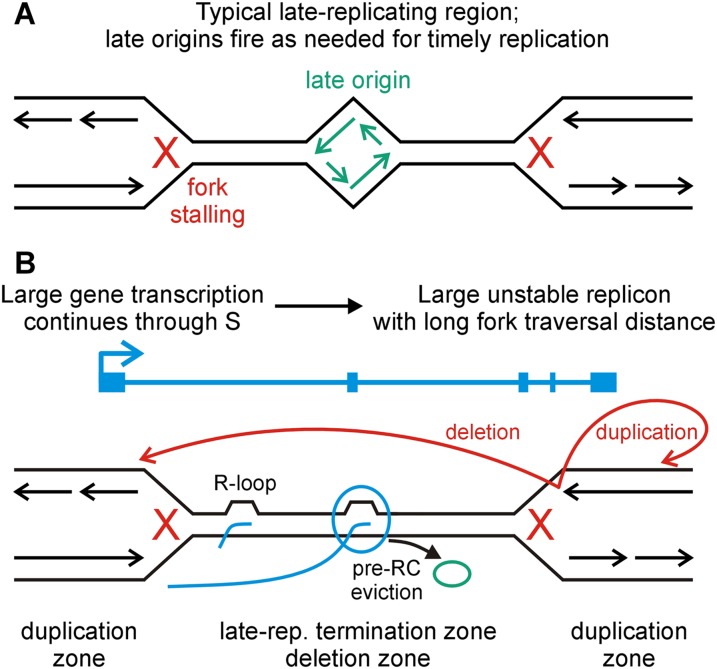
Model for CFS and CNV formation at active large transcription units. (*A*) Replication fork failures, even double-fork failures, occurring in most genomic loci can be rescued by the firing of late “dormant” origins. (*B*) The Transcription-dependent Double-Fork Failure (TrDoFF) model for extreme locus instability under replication stress proposes two mutagenic properties of active large TUs: (1) that they promote simultaneous failure of two converging forks, e.g., through the formation of R-loops; and (2) that they create large late-replicating domains where pre-RC eviction by prolonged transcription into S-phase prevents late origin firing. CFS breaks and deletion CNVs arise in the resulting unreplicated DNA, within the span of the TU, while duplications arise on the flanks, likely by template switching (red arrows).

Bru-seq nascent transcription profiles and large sets of experimentally induced CNVs and CFSs have revealed a synergy between transcription and replication in which replication inhibition disproportionately leads to genomic rearrangements within transcribed genes. TU length predominantly determined the degree of transcription-dependent sensitization to replication stress, with nonrecurrent CNV hotspots and CFSs strongly associated with active TUs > 1 Mb. The combined findings suggest a model in which large TUs define late-replicating domains that promote double-fork failure and extreme locus instability (Fig. 8).

### Transcription as a risk factor for replication-dependent genomic instability

The problem we sought to address was why certain genomic loci are more susceptible to CNVs and CFSs when replication is inhibited. We divided CNV regions into groups ranging from nonrandom hotspots to singleton CNVs. However, the association of transcription with CNV formation in all groups (Fig. 4; Supplemental Table S6) indicates that locus instability is best viewed as a spectrum of risk for a common mechanism. These observations are consistent with substantial literature implicating transcription in genomic instability (Aguilera and Garcia-Muse 2012; Bermejo et al. 2012; Kim and Jinks-Robertson 2012) and refocused the problem to understanding how transcription and impaired replication interact to lead to chromosomal alterations. Here we draw on models of CNV formation in which Fork Stalling and Template Switching (FoSTeS) and Microhomology-Mediated Break-Induced Replication (MMBIR) create de novo junctions at stalled replication forks by invasion of nascent DNA strands into ectopic locations (Lee et al. 2007; Hastings et al. 2009). We also draw on mathematical modeling in which the probability of fork failure at a locus is a function of the distance that a fork must travel (N) divided by the median distance that forks travel prior to stalling (N_s_) (Newman et al. 2013). In this framework, inhibiting replication decreases N_s_ and leads to increased fork failures and FoSTeS and MMBIR events and thus sporadic CNV formation genome-wide.

Extending this logic, increased CNV/CFS risk within active TUs very likely reflects a transcription-dependent increase in fork failure due to replication-transcription collisions, R-loops that impede fork progression, or fork slowing in transcribed chromatin (Fig. 8B; Helmrich et al. 2011; Aguilera and Garcia-Muse 2012). In this view, transcription and replication inhibition would be additive changes to N_s_ within genes. However, replication inhibition and transcription are not independent, since each process must use the same DNA template. Transcription and replication timing are known to be linked (Chakalova et al. 2005; Schwaiger and Schubeler 2006), but this study provides an especially powerful comparison by using Bru-seq to monitor transcription itself, not mature mRNAs, with minimal reliance on gene annotations. Measurements confirmed that the abundant small to moderately sized TUs replicated early in S (Fig. 6). Notably, some regions of this type, especially 9p21.3, did accrue multiple CNVs.

### Transcription-dependent double-fork failure (TrDoFF) at CNV hotspots and CFSs

Beyond the general transcription effect, large TUs displayed disproportionately increased frequencies of spontaneous and replication-stress-induced CNVs and CFSs (Figs. 2–5). A model to explain these results invokes Transcription-dependent Double-Fork Failure (TrDoFF) (Fig. 8). A double-fork failure represents concurrent stalling of two converging replication forks, leading to unreplicated DNA between them. Resolution of unreplicated regions in S/G2 by error-prone replication restart would again lead to CNV formation via FoSTeS/MMBIR.

The TrDoFF model stems from findings that large TUs correspond to late-replicating domains in which forks proceed inward from the boundaries (Figs. 6, 7). Although it is known that CFSs and human nonhomologous CNVs often replicate late (Le Beau et al. 1998; Letessier et al. 2011; Koren et al. 2012), it is the act of transcribing late-replicating DNA that is especially dangerous (Fig. 6). Associated features of the TrDoFF model are, first, that large TUs organize the locations of fork failures in a manner consistent with CNV formation, an idea strongly supported by evidence in Figure 7 and predicted by the MMBIR model, in which forward jumps over unreplicated DNA result in deletions and backward jumps result in duplications (Fig. 8B; Hastings et al. 2009). Further, the nonlinear increase in CNV risk in active large TUs (Fig. 4) follows from models that the probability of double-fork failure increases as the square of the N/N_s_ ratio (Newman et al. 2013), where large TUs have both a large interorigin distance (N) and likely a reduced median stalling distance (N_s_).

Many observations indicate that active large TUs are the causative factor in the TrDoFF model. First, loci switched from unstable to stable in different cell lines expressing or not expressing long gene isoforms, respectively (Table 1; Fig. 5). Moreover, TU length correlated with replication timing across all genes and data sets (Fig. 6), with the replication delay most striking in the middle of large TUs (Fig. 7; Supplemental Fig. S11). The inferred paucity of usable origins within large TUs is consistent with observations using DNA combing in selected CFSs of reduced firing of late origins that typically remain dormant but fire under replication stress (Fig. 8A; Letessier et al. 2011; Ozeri-Galai et al. 2011). Importantly, pre-replication complexes (pre-RCs) are only licensed in G1 and must remain bound for firing to occur in S-phase. Movement of RNA polymerase through an origin can sweep away a pre-RC, as demonstrated in yeast (Snyder et al. 1988; Looke et al. 2010). We thus suggest that dormant origins do not rescue TrDoFFs at CNV hotspots/CFSs because expansive locus transcription has persisted into S and removed those pre-RCs before they can be utilized (Fig. 8B).

### CNV hotspots and CFSs are the same cell-type-specific loci

Our data show that CNV hotspots and CFSs are different manifestations of the same mechanistic process driven by large TUs (Figs. 2, 5, 8). Many CFSs are known to lie in large genes (Smith et al. 2006), but there are many possible reasons for this association, including that large introns have high levels of A/T rich sequences (Zlotorynski et al. 2003). Our finding that transcription of large genes can predict CFS localization is consistent with a proposal that instability at three CFSs was caused by replication-transcription conflicts (Helmrich et al. 2011). It disagrees with a conclusion arrived at using ENCODE data that transcription does not dictate CFS fragility (Le Tallec et al. 2013), emphasizing the importance of measuring transcript isoforms in the specific cells under study. Even two primary human fibroblast lines had transcription differences that corresponded to the locations of CNV hotspots and CFSs (Table 1; Fig. 5), demonstrating the exquisite cell-type specificity of the phenomenon. Thus, differences in the transcription of large genes can explain the cell type differences in CFS expression patterns.

### Implications for human genomic disorders and cancer

In total, transcription of large genes in dividing cells appears to set up a “perfect storm” of instability risk by subjecting them to a high risk for TrDoFFs, yet preventing them from resolving these critical lesions. Expression of such genes might in part be an artifact of cells coerced to divide repeatedly in vitro. However, many of the large genes at our CNV hotpots and CFSs are expressed in dividing cells in vivo, including neuronal stem cell layers in the brain (Ayoub et al. 2011), where a combination of large TUs and replication might contribute to the reported high frequency of brain somatic CNVs (McConnell et al. 2013). The resulting mosaicism for gene copy number could have important consequences for developmental and age-related (dys)function.

Finally, CNV hotspots in cultured cells correspond well to a subclass of clinically relevant nonrecurrent human CNVs that similarly lie within large genes (Supplemental Table S9 and references therein). For example, constitutional intragenic deletions within *AUTS2, IMMP2L,* and *NRXN1* have been associated with autism spectrum disorder, intellectual disability, and psychiatric disorders. Deletions in many of our hotspot genes (e.g., *NRXN1*, *PDE4D*, *WWOX*, *LSAMP*, and *NEGR1*) are also found in numerous cancers where they likely represent the effects of perturbed replication and transcription. Other large genes with CNVs in human disorders were not expressed in the cell types used here, such as *CNTNAP2*, in which intragenic CNVs are found in several neurodevelopmental disorders. However, the TrDoFF model predicts that large genes will be CNV hotspots in the replicating cell types in which they are expressed, which could include neuronal cells, germ cell precursors, and early post zygotic cells.

## Methods

### Cell types and CNVs

Human 090 fibroblast and mES cell lines and CNVs have been described previously (Arlt et al. 2009, 2011, 2012, 2014). CNVs in 090 cells were detected using Illumina HumanOmni1 and HumanOmni2.5 BeadChip SNP microarrays and NimbleGen 12 × 270k array comparative genome hybridization (aCGH). CNVs in mES cells were detected using NimbleGen 3 × 720k aCGH. The UMHF1 (HF1) human foreskin fibroblast cell line is the same as used in descriptions of Bru-seq (Paulsen et al. 2013b). HF1 was treated with 0.4 uM APH for 72 h, cell clones made, and de novo CNVs detected using the HumanOmni2.5 BeadChip.

### Common fragile site assessment and FISH

CFS breakage was induced by exposure of 090 or HF1 fibroblasts to 0.4 µM APH for 24–36 h prior to cell harvest for metaphase chromosome preparations. Cells were fixed onto slides for Giemsa banding or FISH. Chromosome breaks and gaps were analyzed in 100 metaphases from each cell line. At selected loci, the locations of fragile site breaks were refined using BAC FISH probes obtained from BlueGnome (RP5-837C9 and RP11-479M23) hybridized to metaphase spreads. For replication timing, BlueGnome probes RP11-151P8 and RP11-324H4 were hybridized to interphase nuclei and compared to the RP11-489O1 control probe by scoring the percentage of chromosomes with replicated probes with two adjacent FISH signals (Le Beau et al. 1998).

### SNP PCR detection of de novo CNVs

To detect HF1 CNVs overlapping specific loci, we designed PCR primers flanking informative SNPs that conferred a restriction fragment length polymorphism (RFLP). The presence of only restriction-enzyme cleavable or uncleavable products indicated LOH and the presence of a deletion CNV, subsequently confirmed by sequencing. The number of 090 CNV alleles detected by microarray and HF1 CNV alleles detected by microarray or SNP PCR were compared using Fisher’s exact test relative to the total number of alleles tested.

### Bioinformatics

Data analysis pipelines are described in detail in Supplemental Methods or, in the case of Bru-seq, were previously published (Paulsen et al. 2013a). Custom scripts in Perl, R and other languages were wrapped into workflows using the q pipeline manager (http://sourceforge.net/projects/q-ppln-mngr/). Many analyses used the BEDTools (Quinlan and Hall 2010) or q-associated bedutil utilities. Steps included creating a union of overlapping CNVs, as well as intervening gaps <750 kb, for conversion into CNV regions, randomized placement of CNV regions throughout the genome to create simulations, identification of overlaps between the entire span of the resulting CNV regions, and various classes of genomic features, and characterizing the overlaps by Boolean, fractional overlap, or length-weighted average scores. The hg19 and mm9 reference genomes were used for human and mouse data, respectively, and the corresponding Ensembl transcript annotations updated January 1, 2014 (Flicek et al. 2014).

### Bru-seq nascent RNA sequencing

Bru-seq methods were previously described (Paulsen et al. 2013a). Human 090 fibroblasts were processed by growing cells in the absence or presence of 0.4 μM APH for 24 h, exposing them to Bru for 30 min in the same media, immunoprecipitating labeled RNAs, constructing strand-specific RNA-seq libraries, sequencing using Illumina HiSeq, and mapping reads to the reference genome. mES cells were first passed three times on gelatin-coated plates to remove the metabolically active feeder cells that would confound Bru-seq analysis while maintaining the undifferentiated state. Only then were cells grown with or without 0.6 uM APH for 24 h and Bru-seq performed as above. TUs were identified as contiguous genome spans where the RPKM signal intensity exceeded a threshold empirically determined to correspond to active transcription (Supplemental Figure S4).

### Replication timing

IMR-90 and BJ fibroblast as well as GM12878 lymphoblastoid Repli-seq data were obtained from ENCODE Project Consortium repositories (Hansen et al. 2010), with minor modifications and extensions to data analysis as described in Supplemental Methods. mES cell Repli-chip data were obtained from the Gilbert laboratory (http://www.replicationdomain.com/; Hiratani et al. 2008) and used as provided with additional projection of replication timing ratios to the genomic space between microarray probes.

## Data access

The Bru-seq data from this study have been submitted to the NCBI Gene Expression Omnibus (GEO; http://www.ncbi.nlm.nih.gov/geo/) under accession number GSE55862.

## Supplementary Material

Supplemental Material
