# Supplementary material for: Large transcription units unify copy number variants and common fragile sites arising under replication stress
Source: Genome Res. 2015 Feb;25(2):189–200. doi: 10.1101/gr.177121.114 (PMC4315293; doi:10.1101/gr.177121.114)
Supplement: Supplemental Material [file supp_25_2_189__index.html]

Large transcription units unify copy number variants and common fragile sites arising under replication stress — Large transcription units unify copy number variants and common fragile sites arising under replication stress — Supplemental Material 

# Large transcription units unify copy number variants and common fragile sites arising under replication stress

## Supplemental Material

**Files in this Data Supplement:**

- FigureS1G.pdf
- FigureS1H.pdf
- Supplemental Material.pdf
- FigureS8H.gif
- FigureS8I.gif
- TableS3A.xlsx
- TableS3B.xlsx
- TableS3C.xlsx
- TableS3D.xlsx
- TableS4.xlsx
- TableS5.xlsx
