# Supplementary figures and images for: Large transcription units unify copy number variants and common fragile sites arising under replication stress
Source: Genome Res. 2015 Feb;25(2):189–200. doi: 10.1101/gr.177121.114 (PMC4315293; doi:10.1101/gr.177121.114)

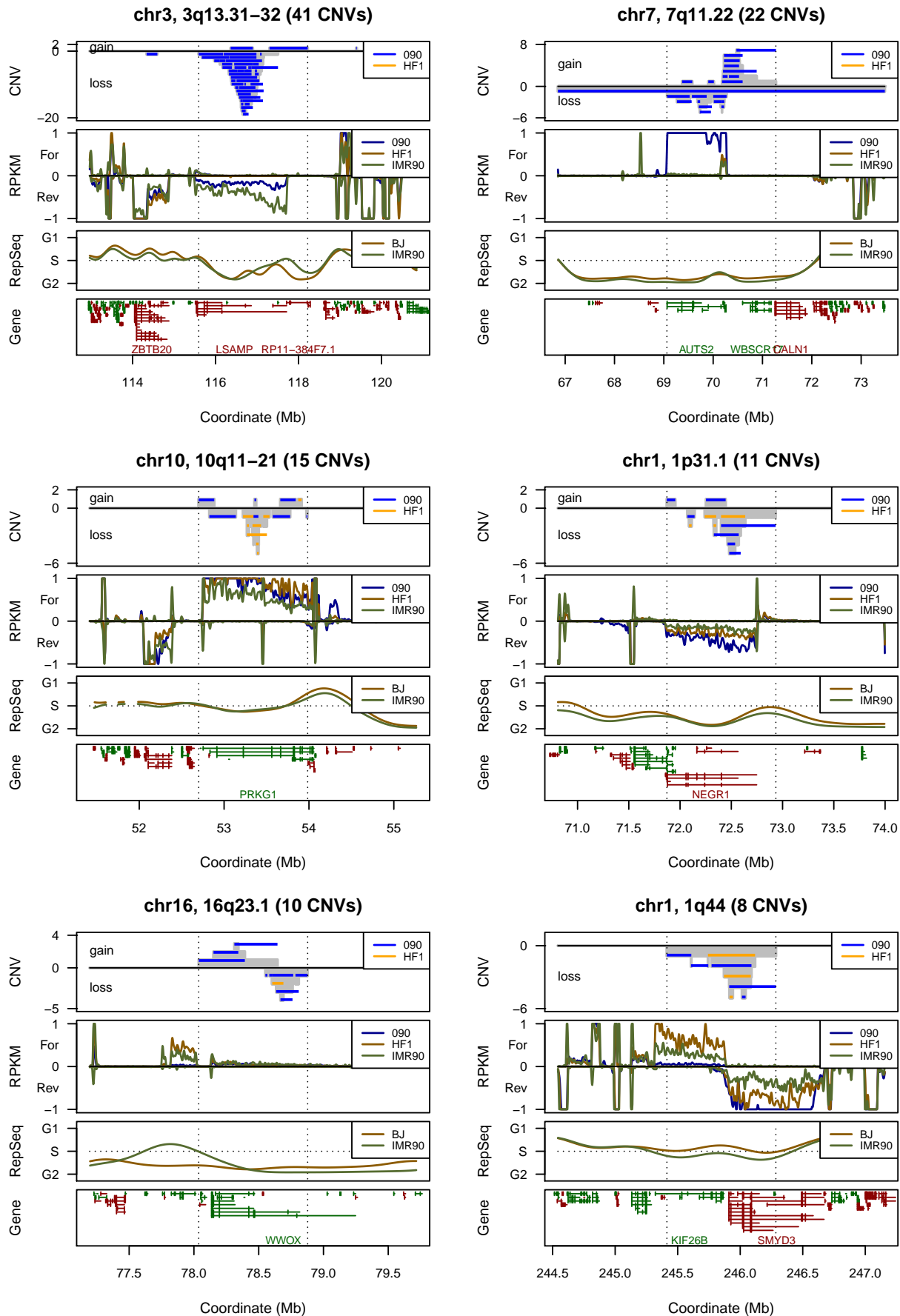

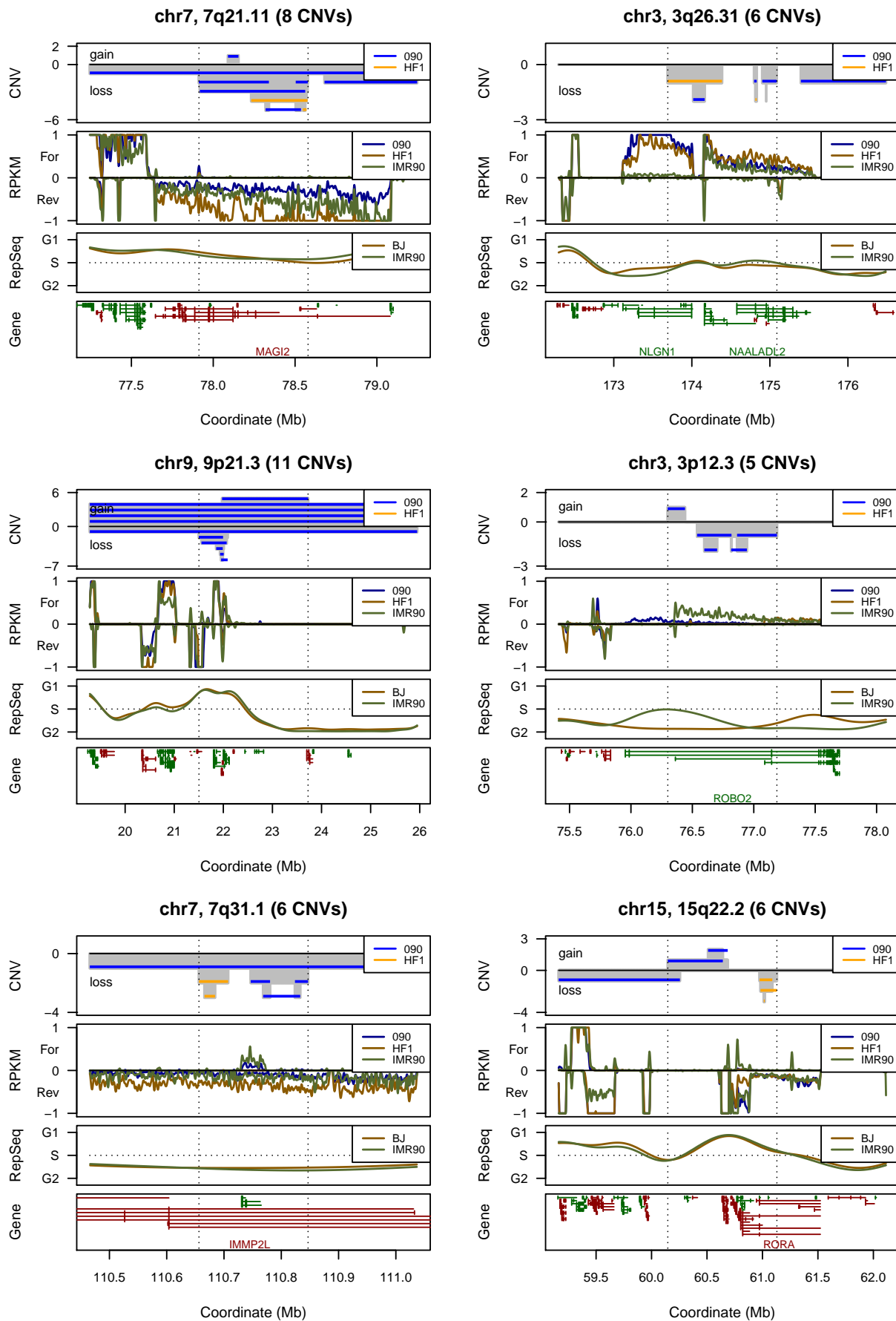

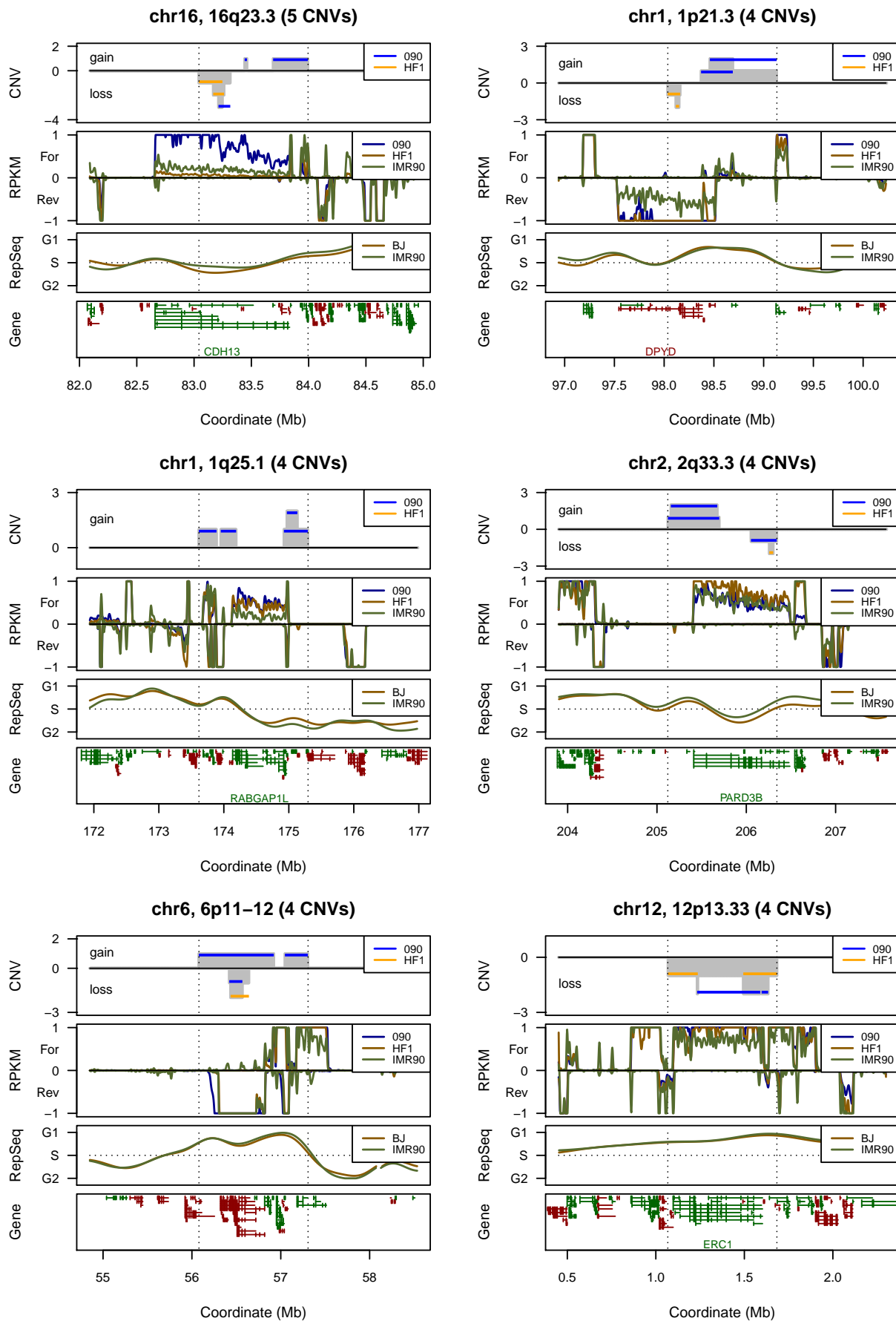

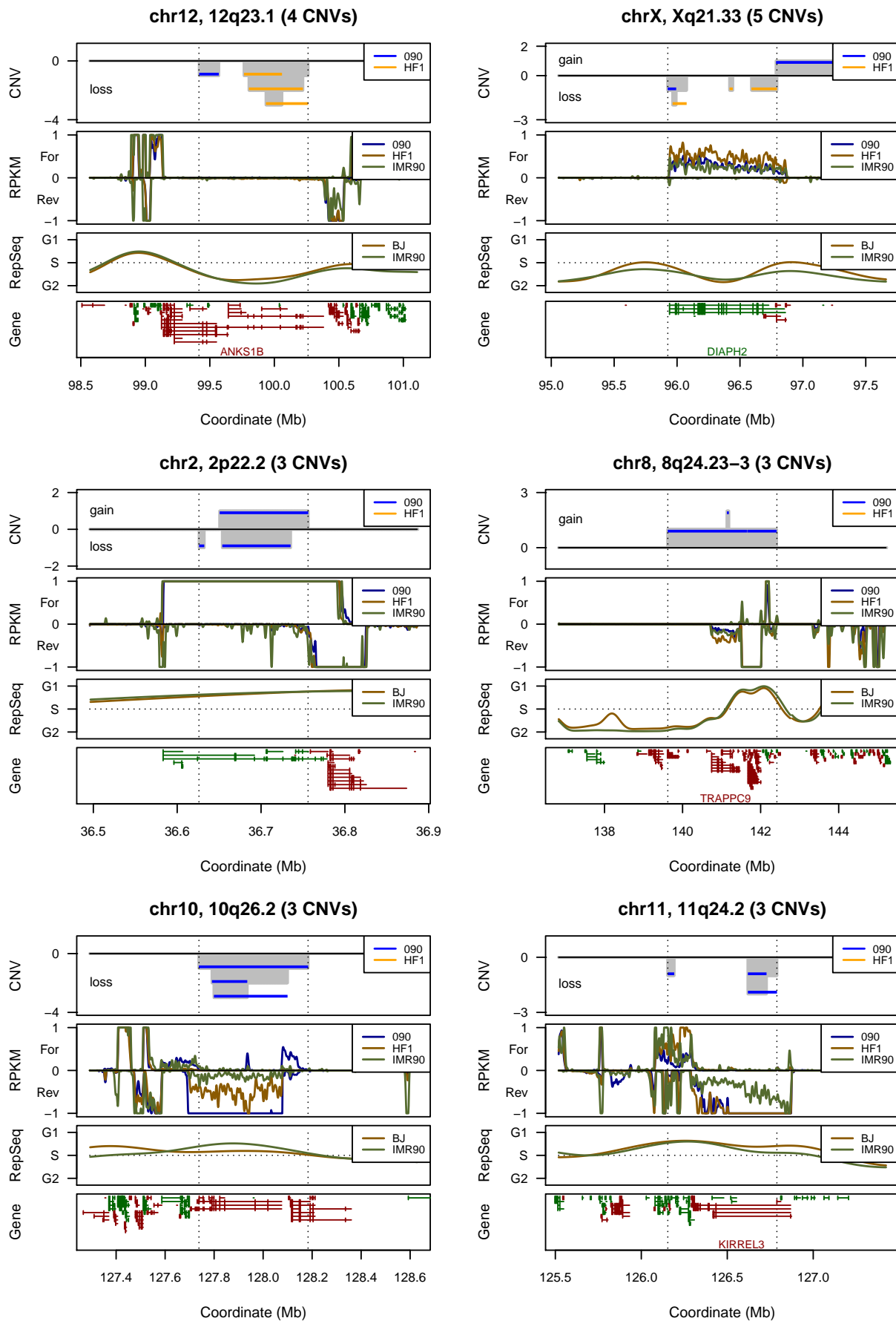

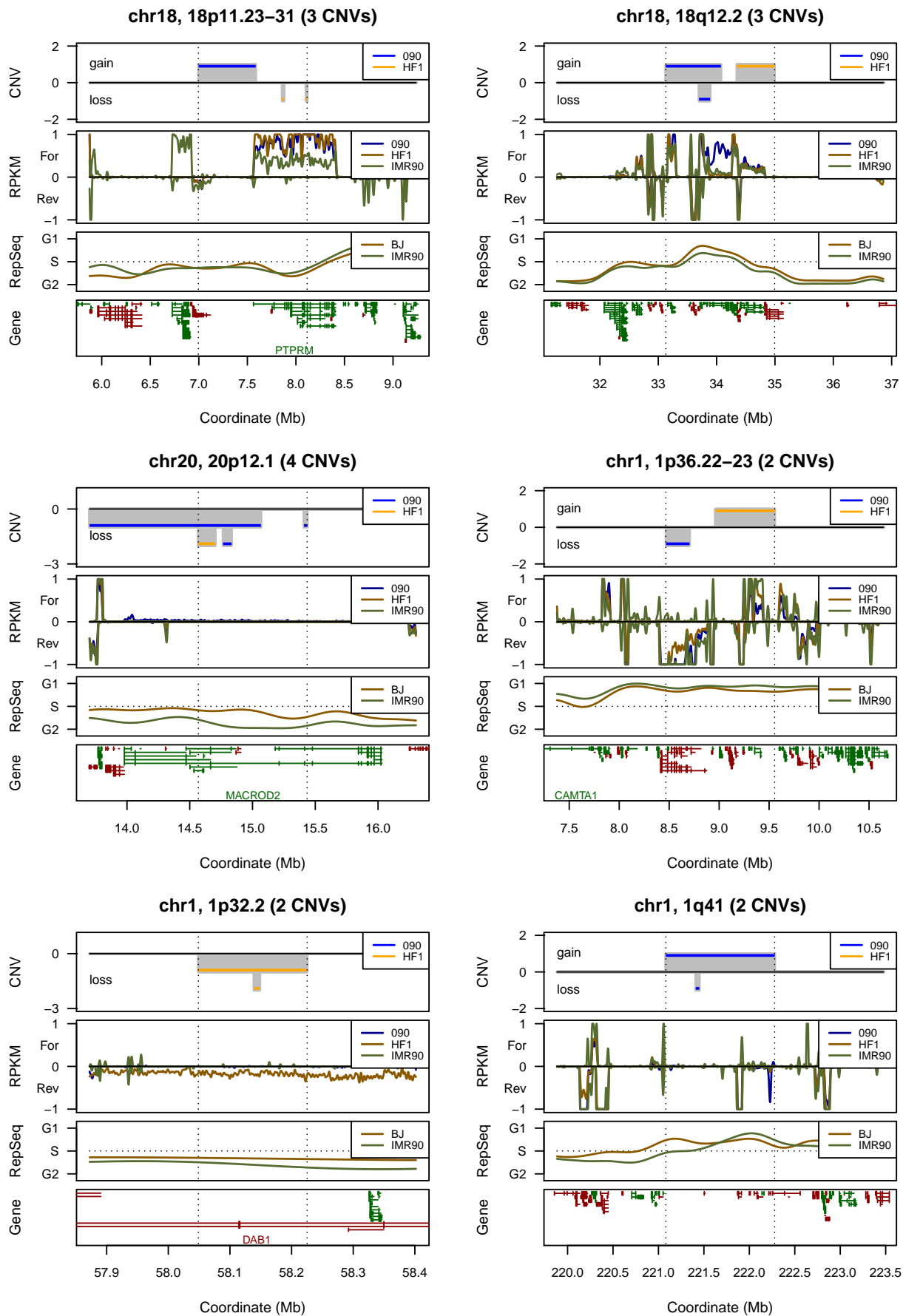

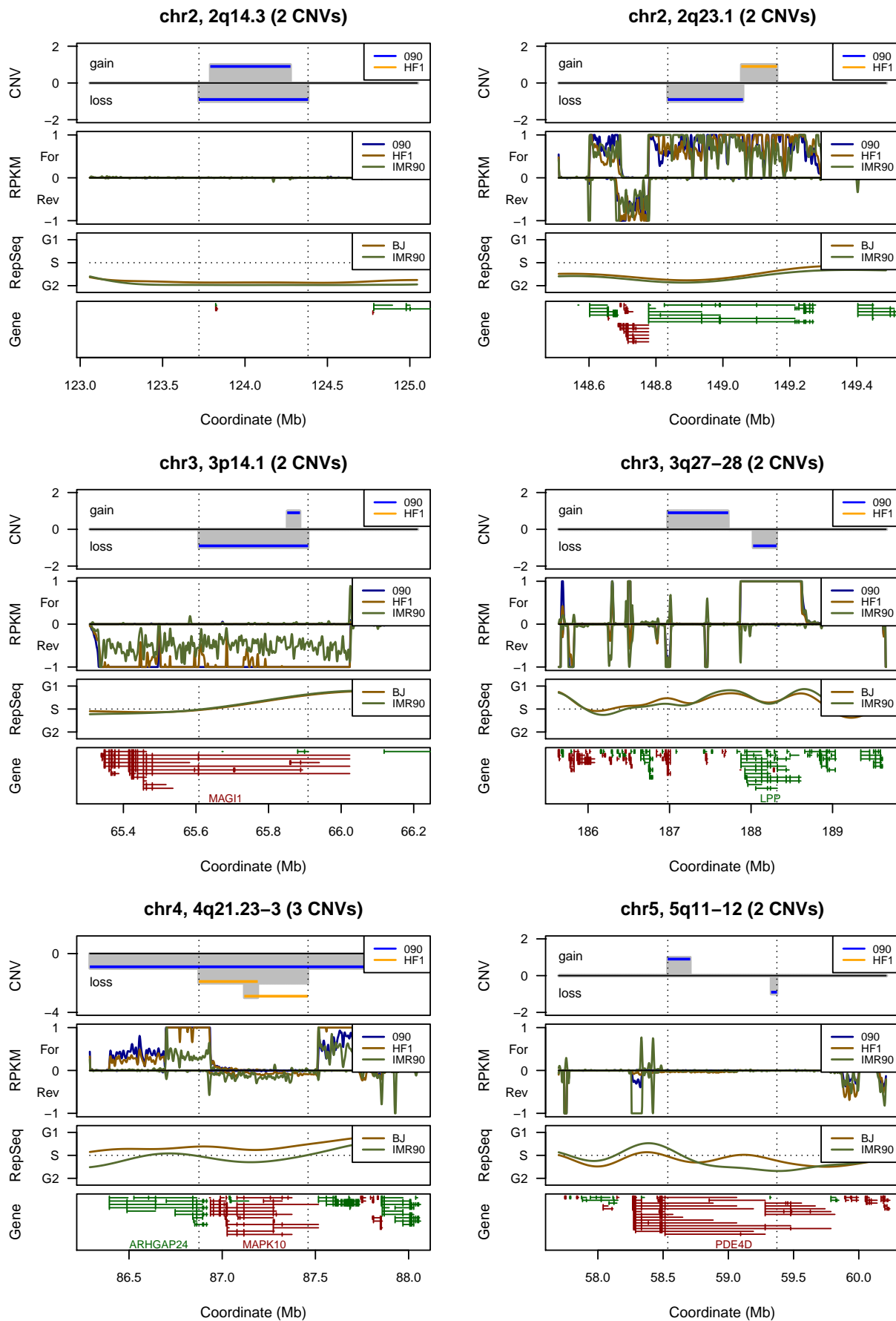

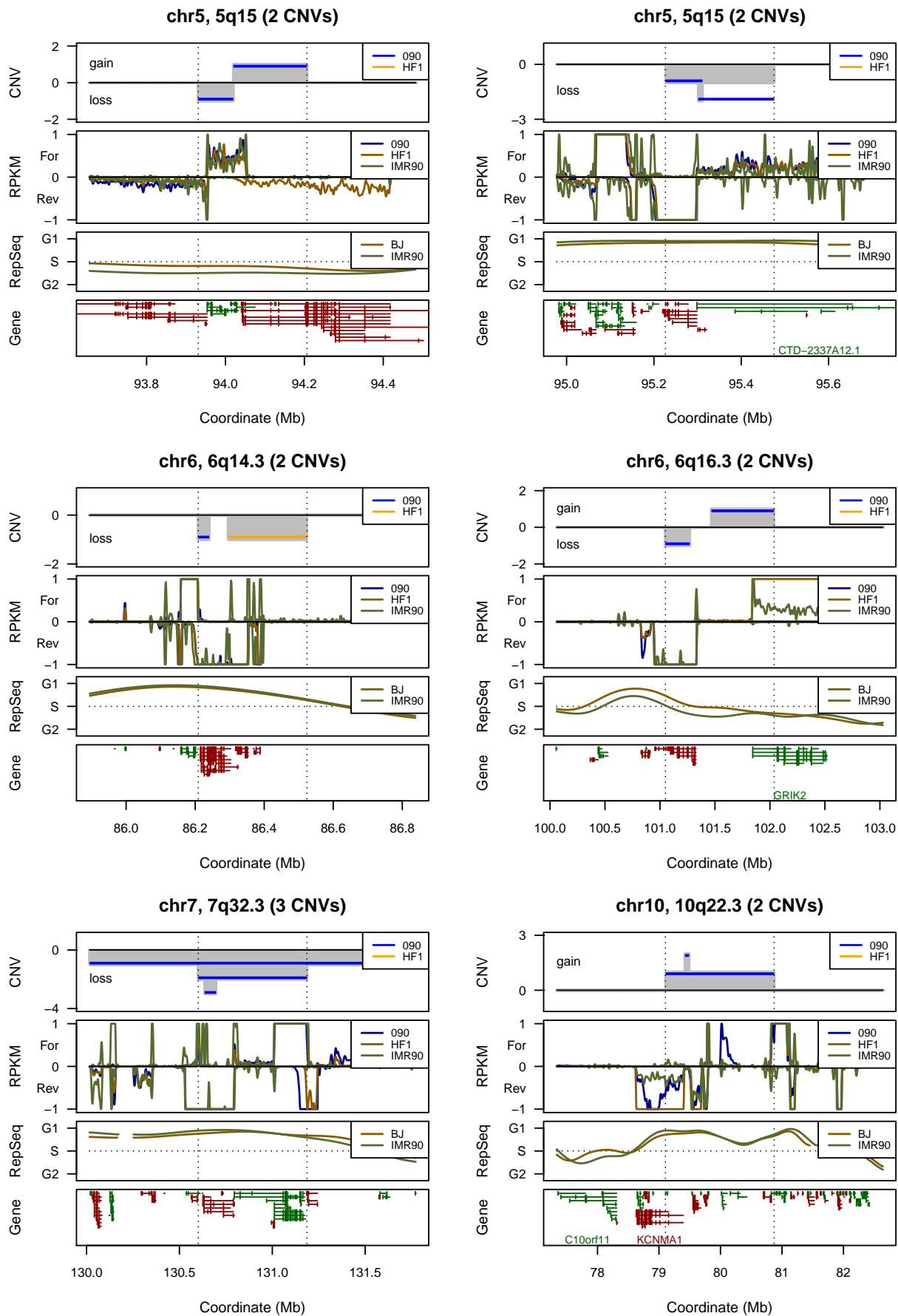

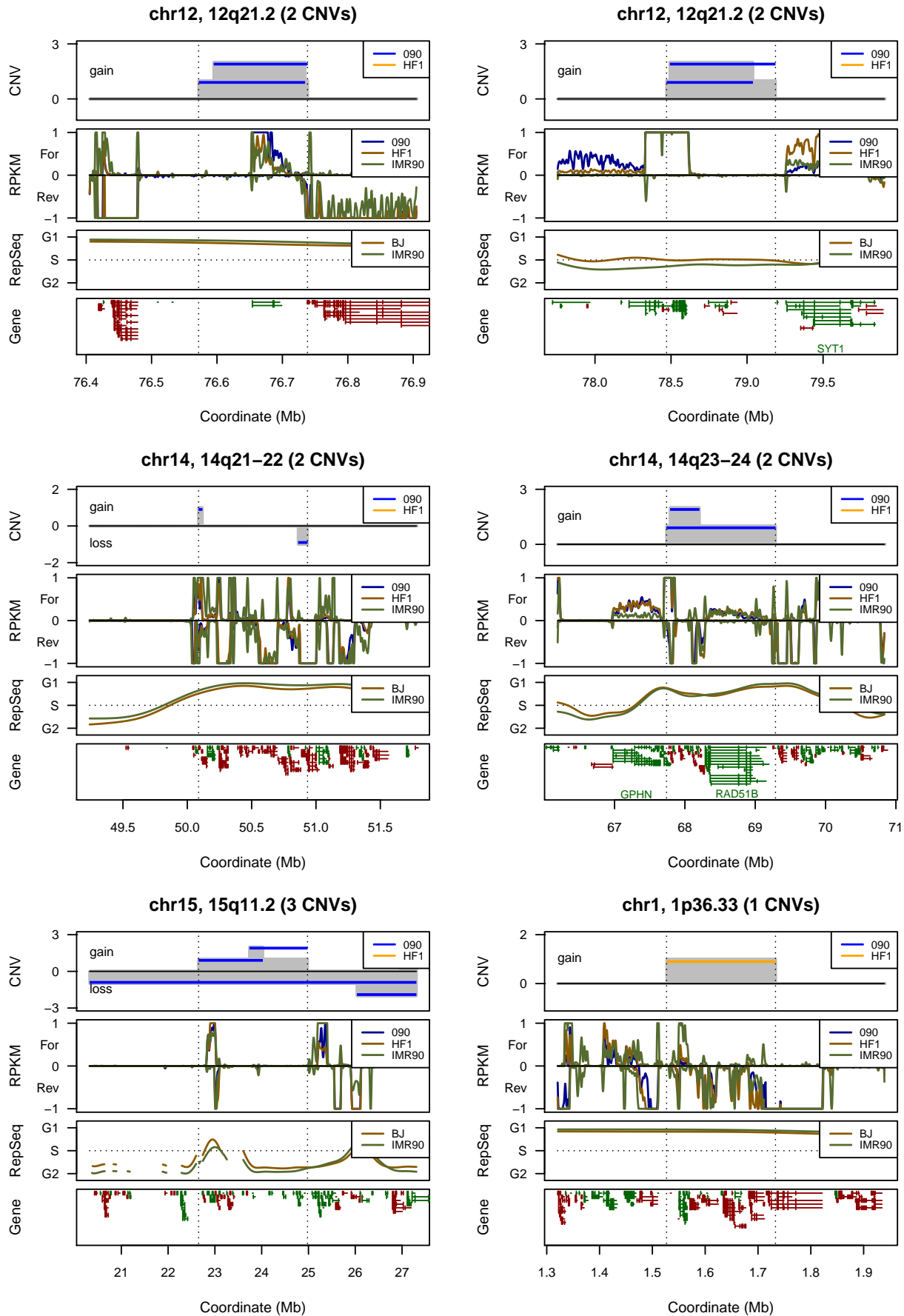

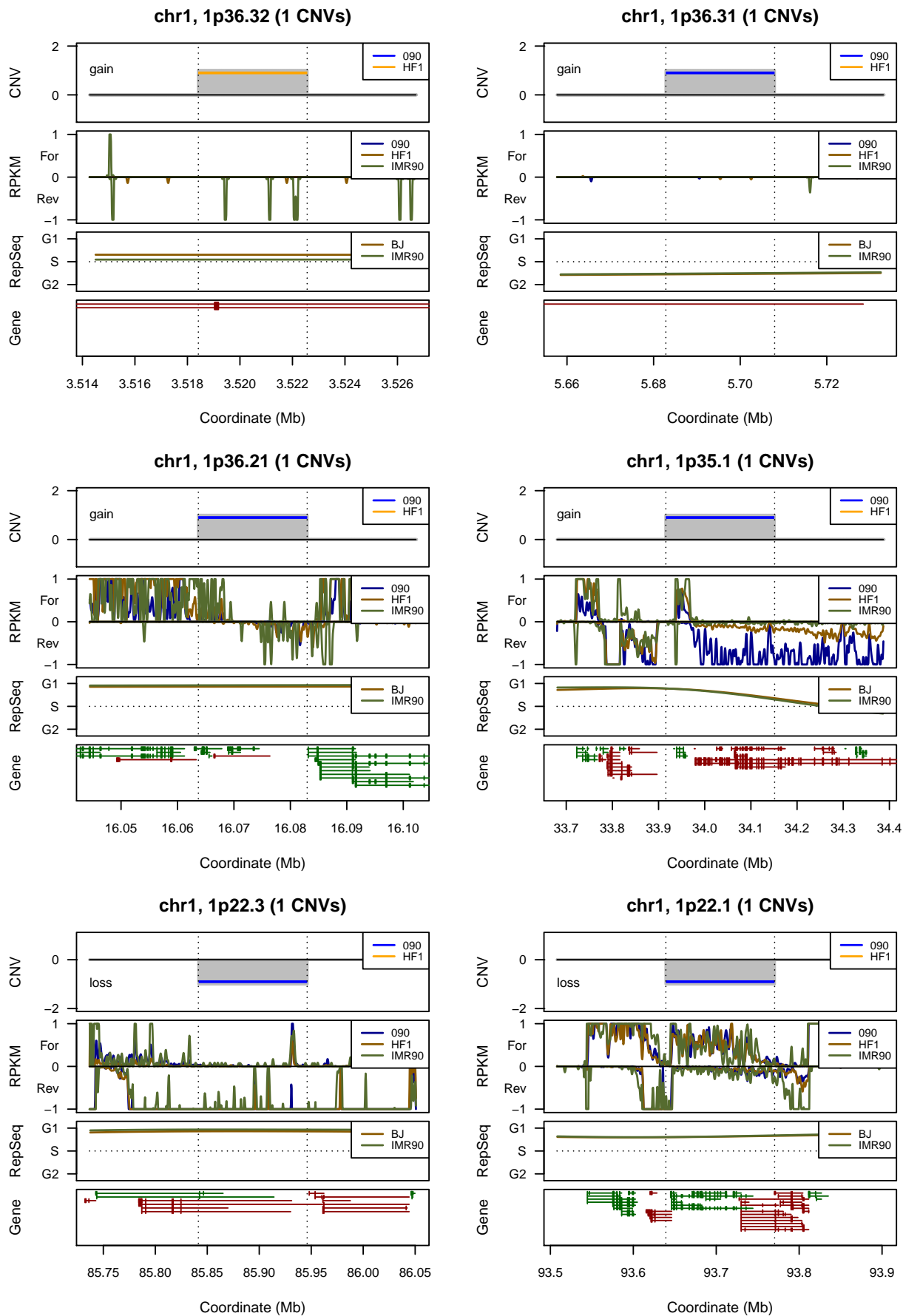

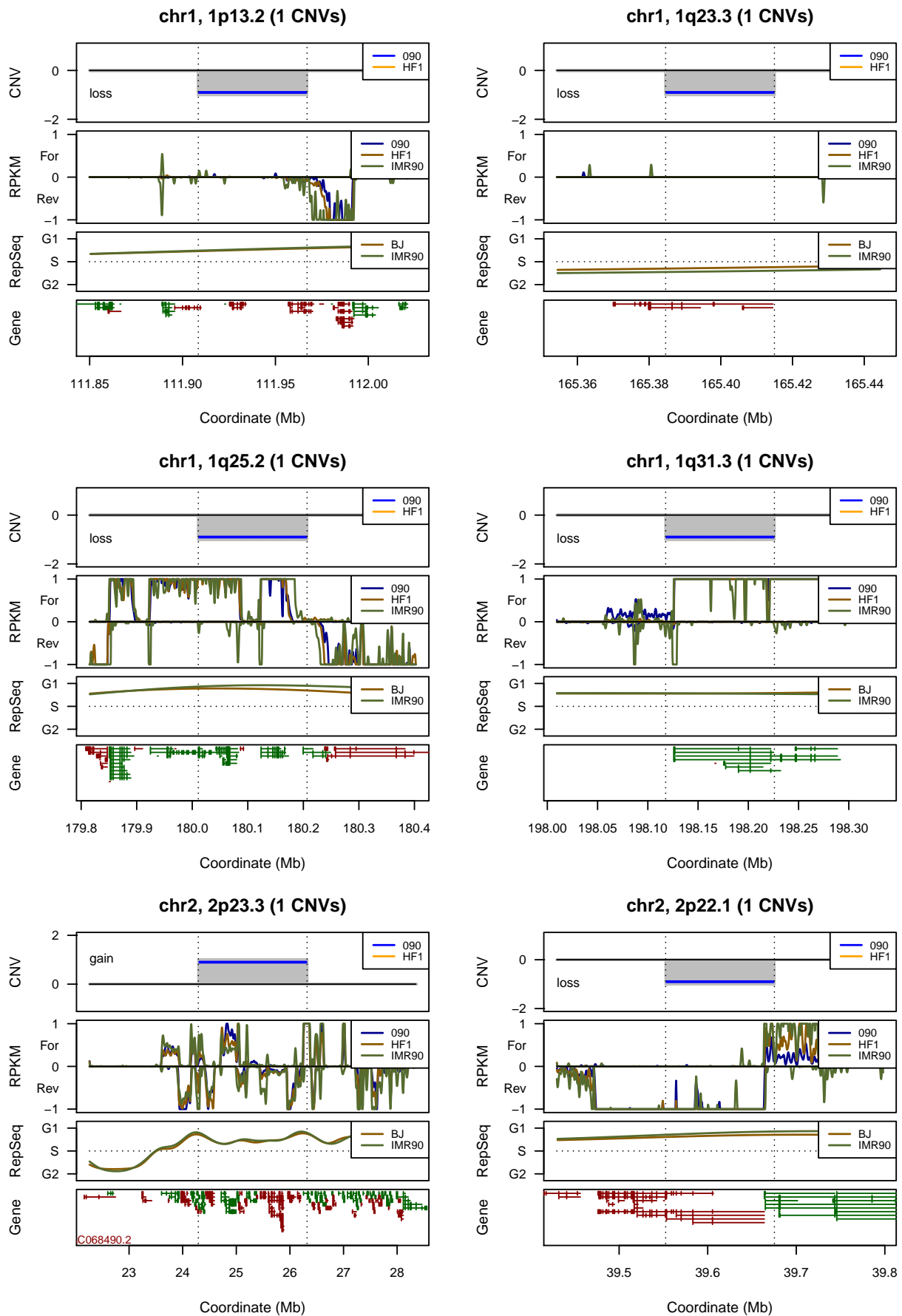

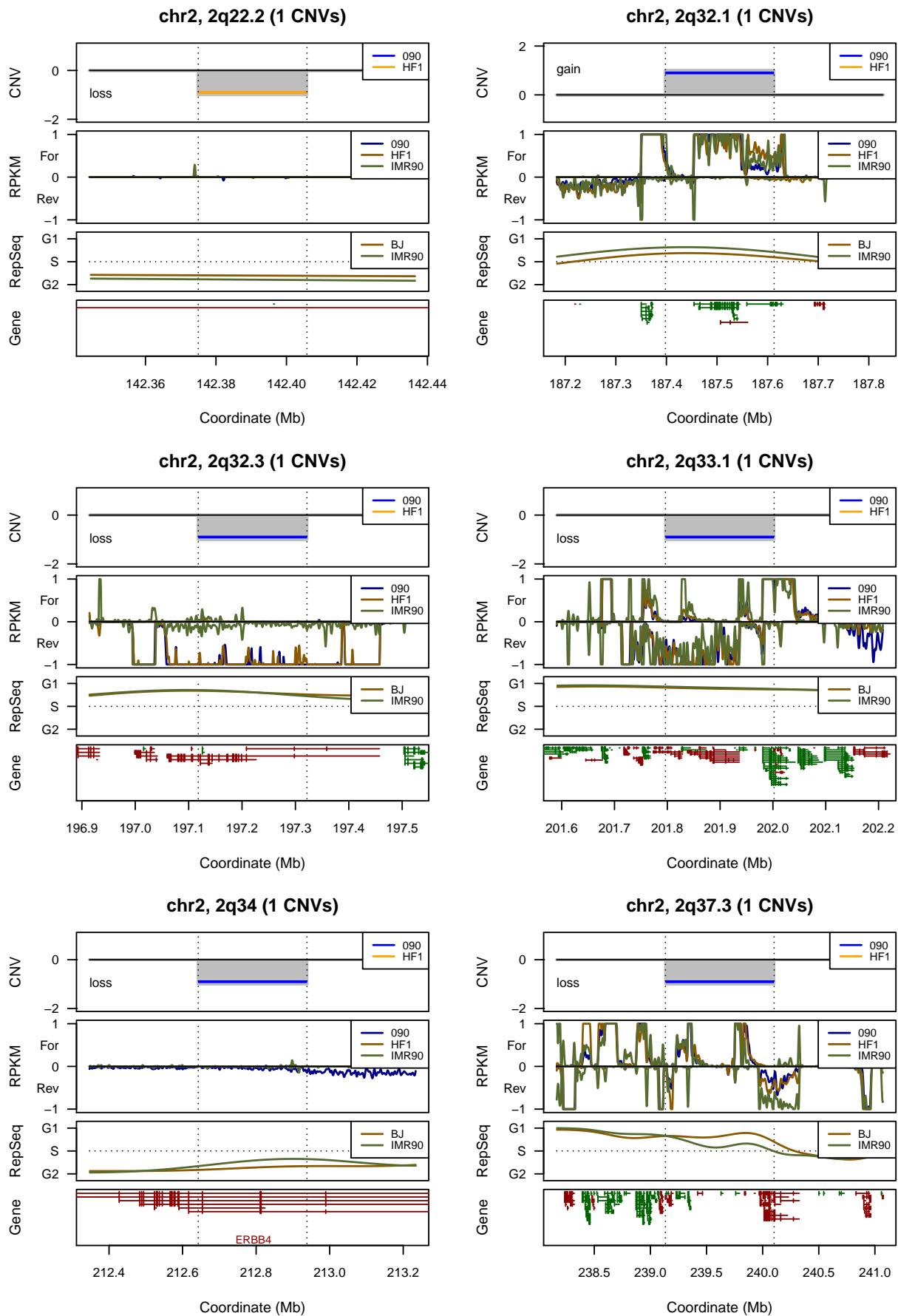

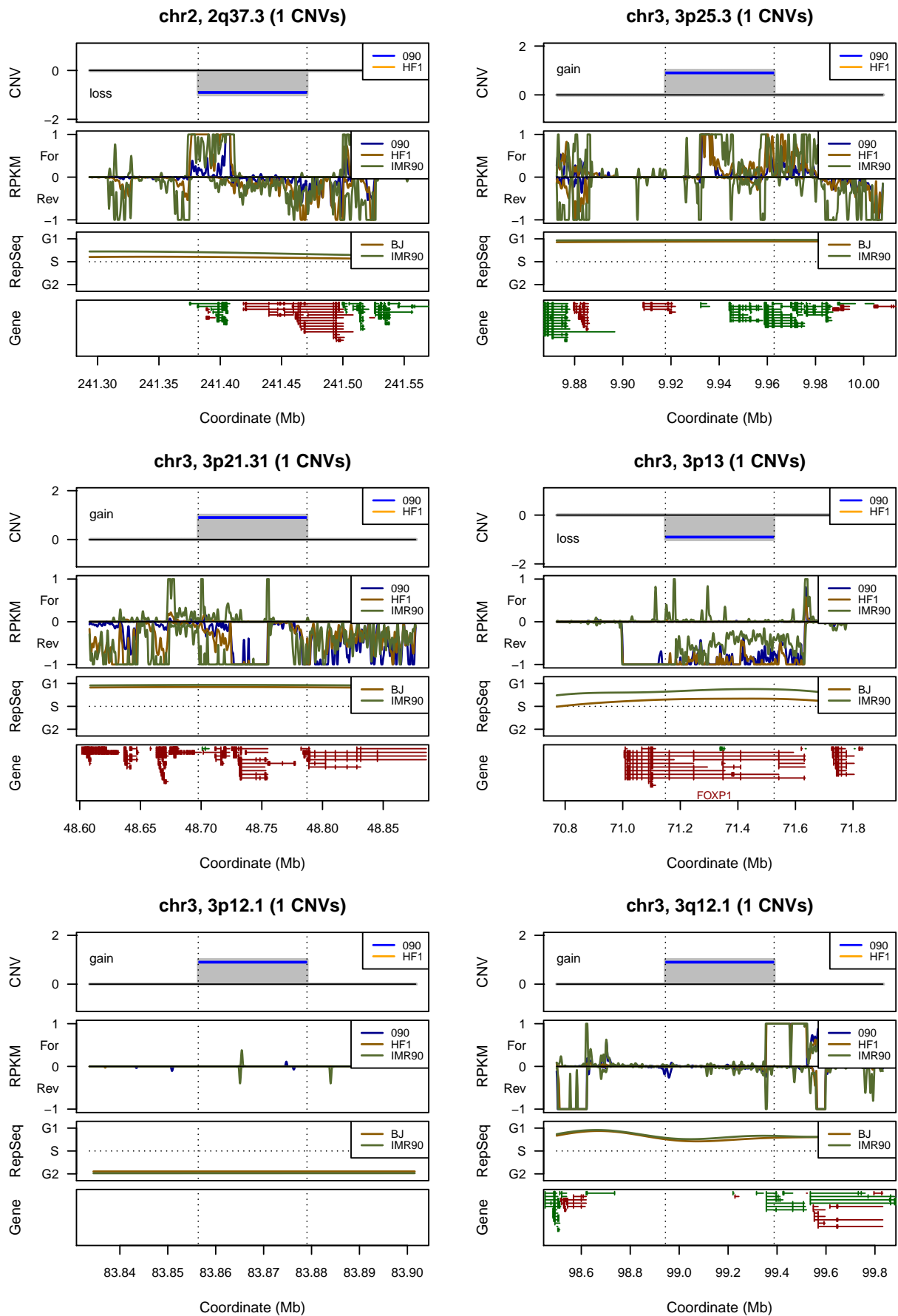

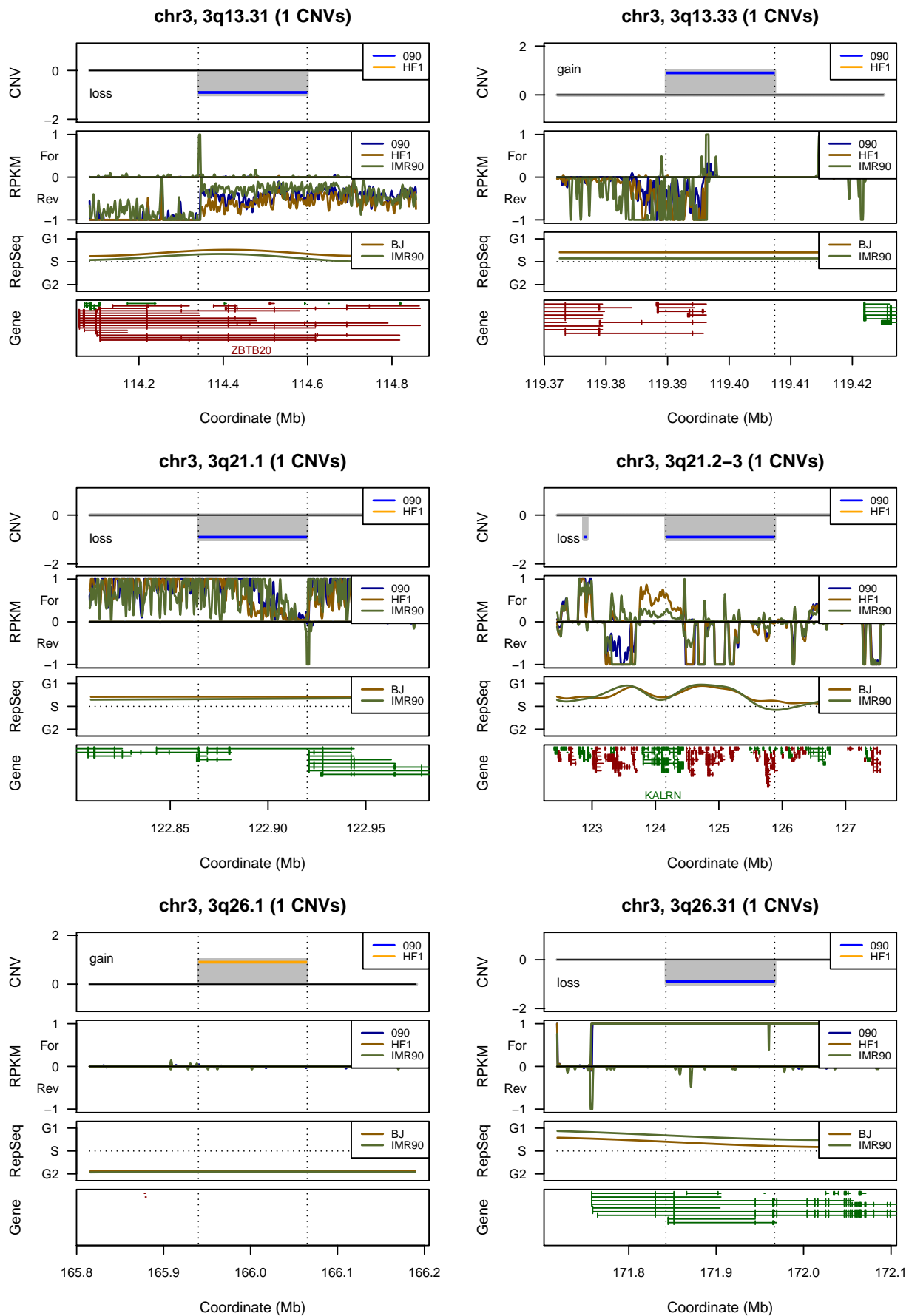

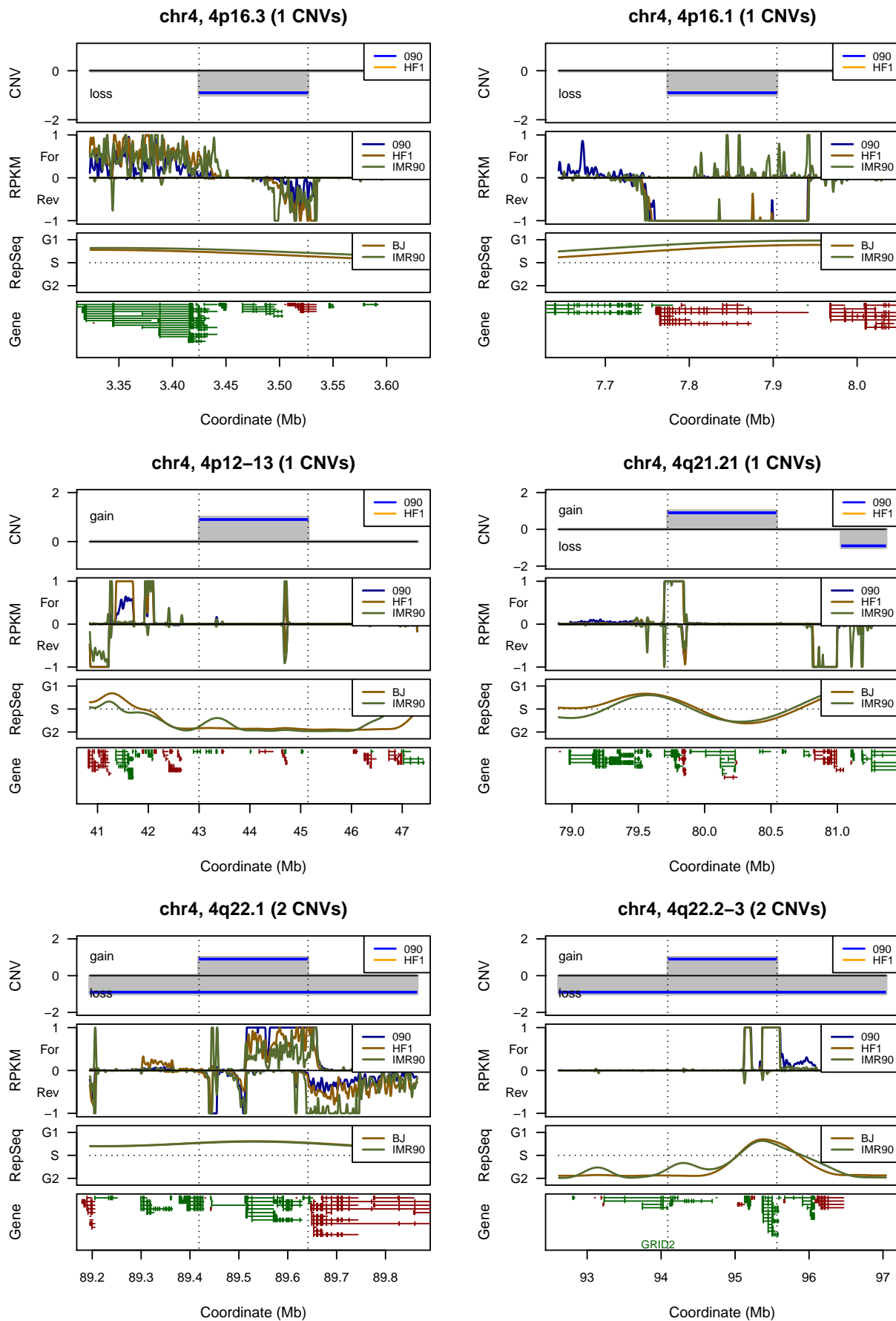

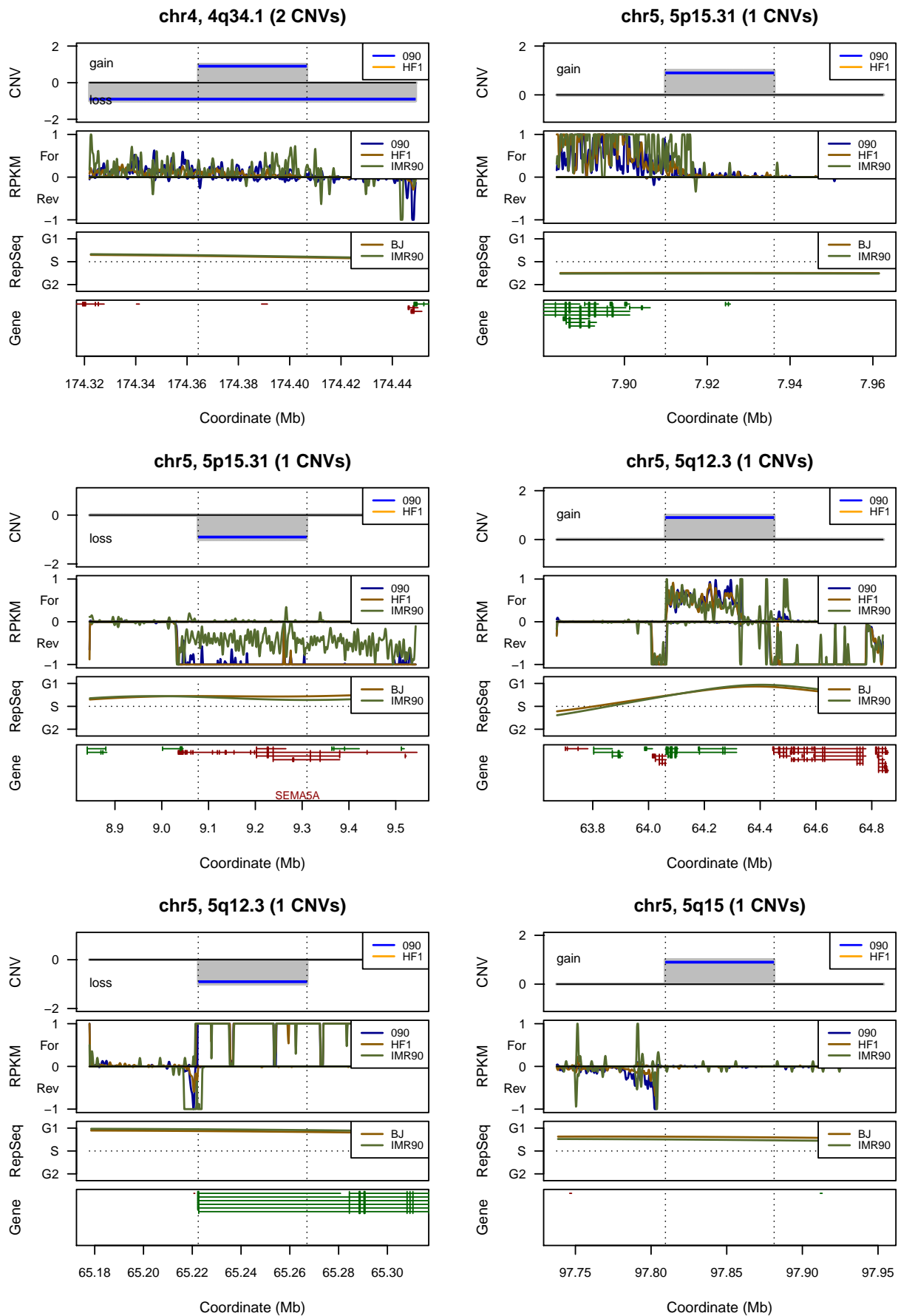

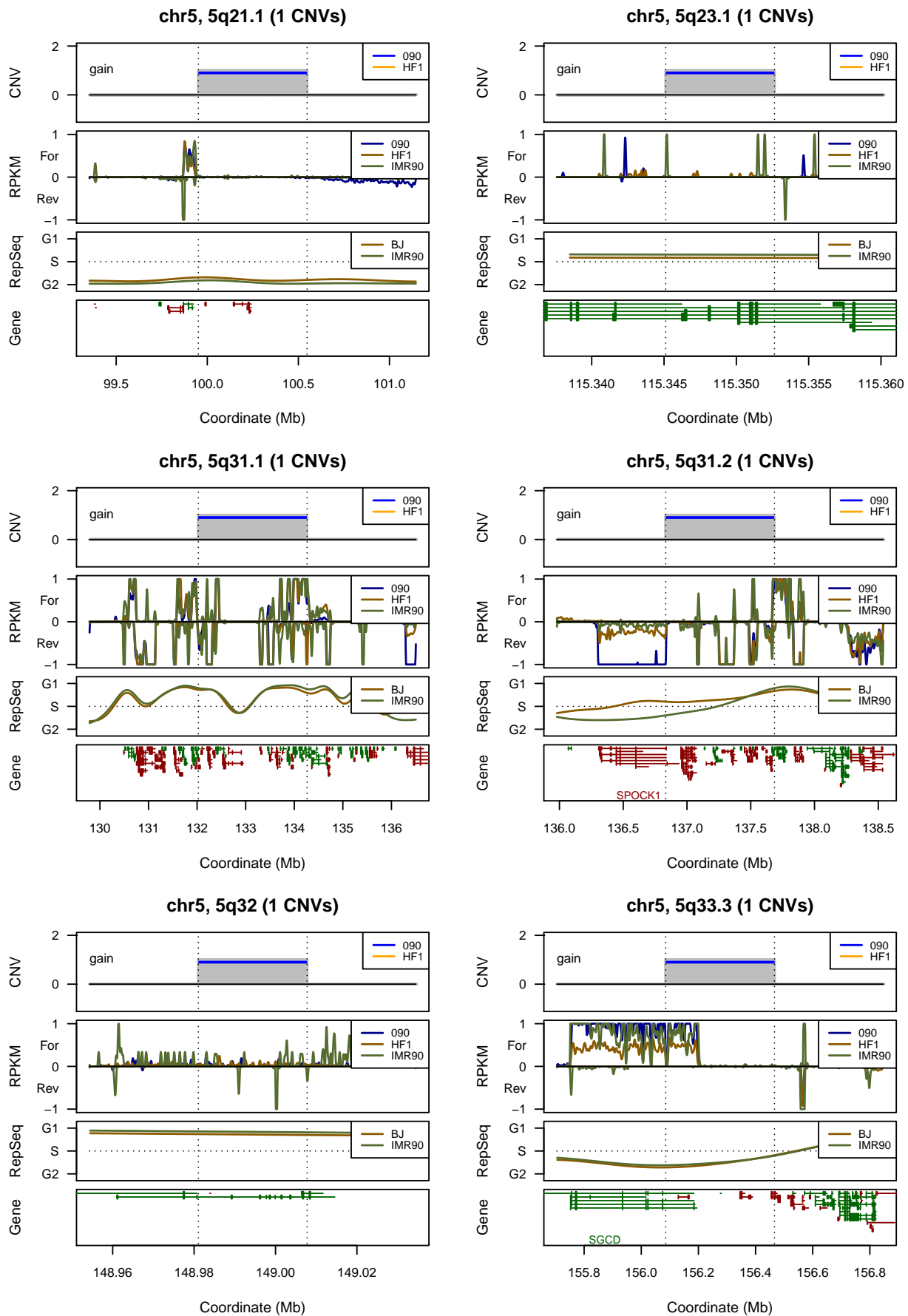

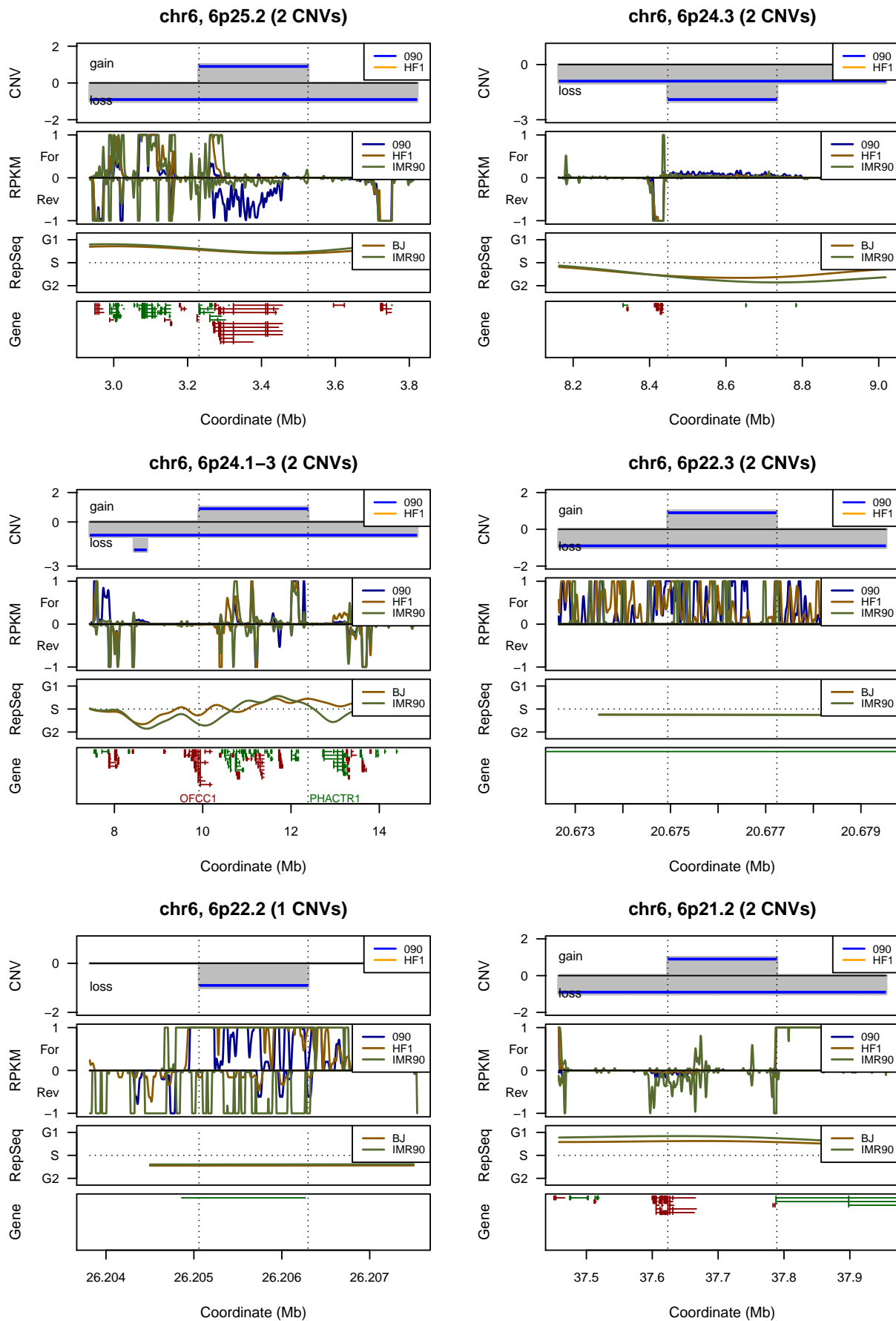

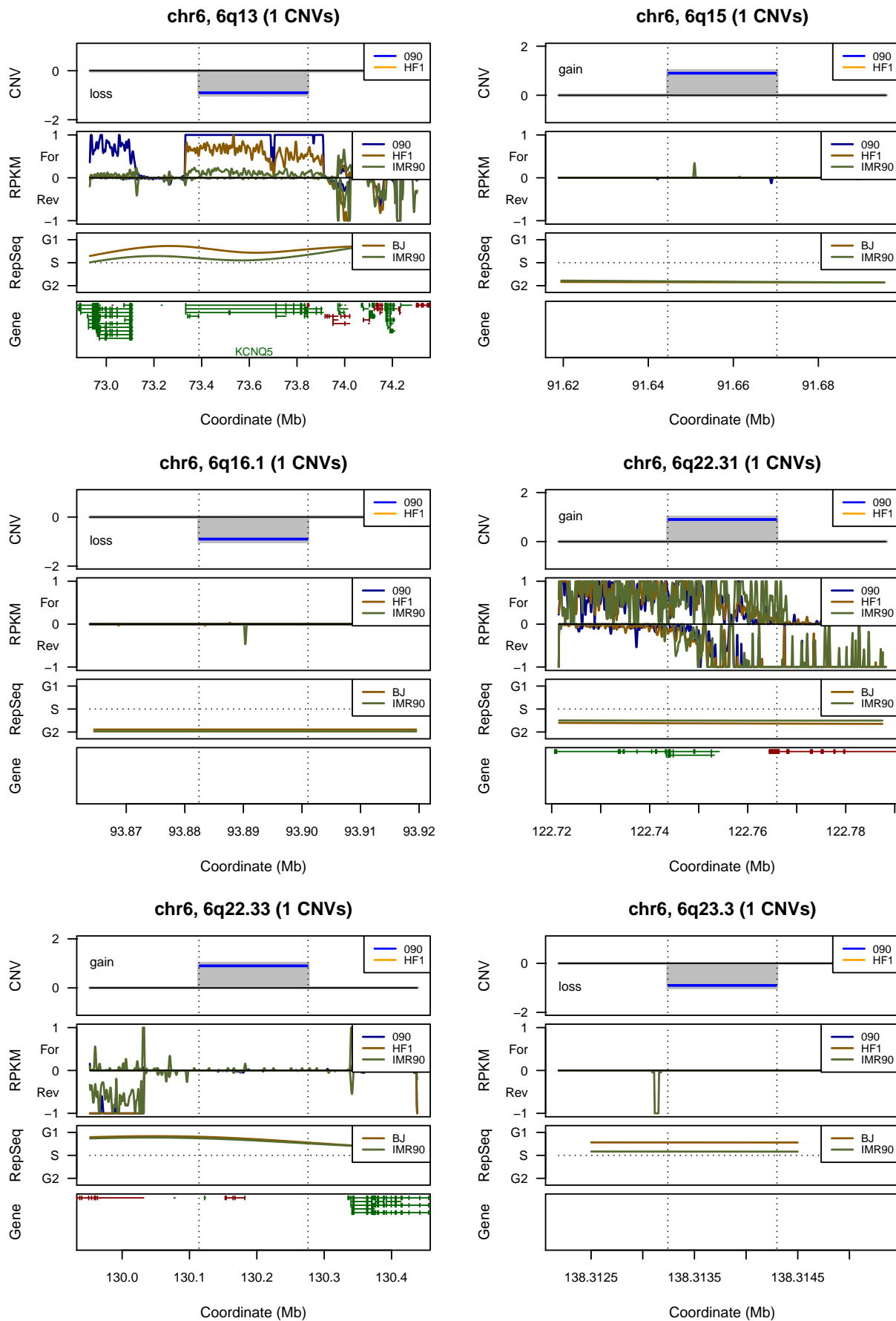

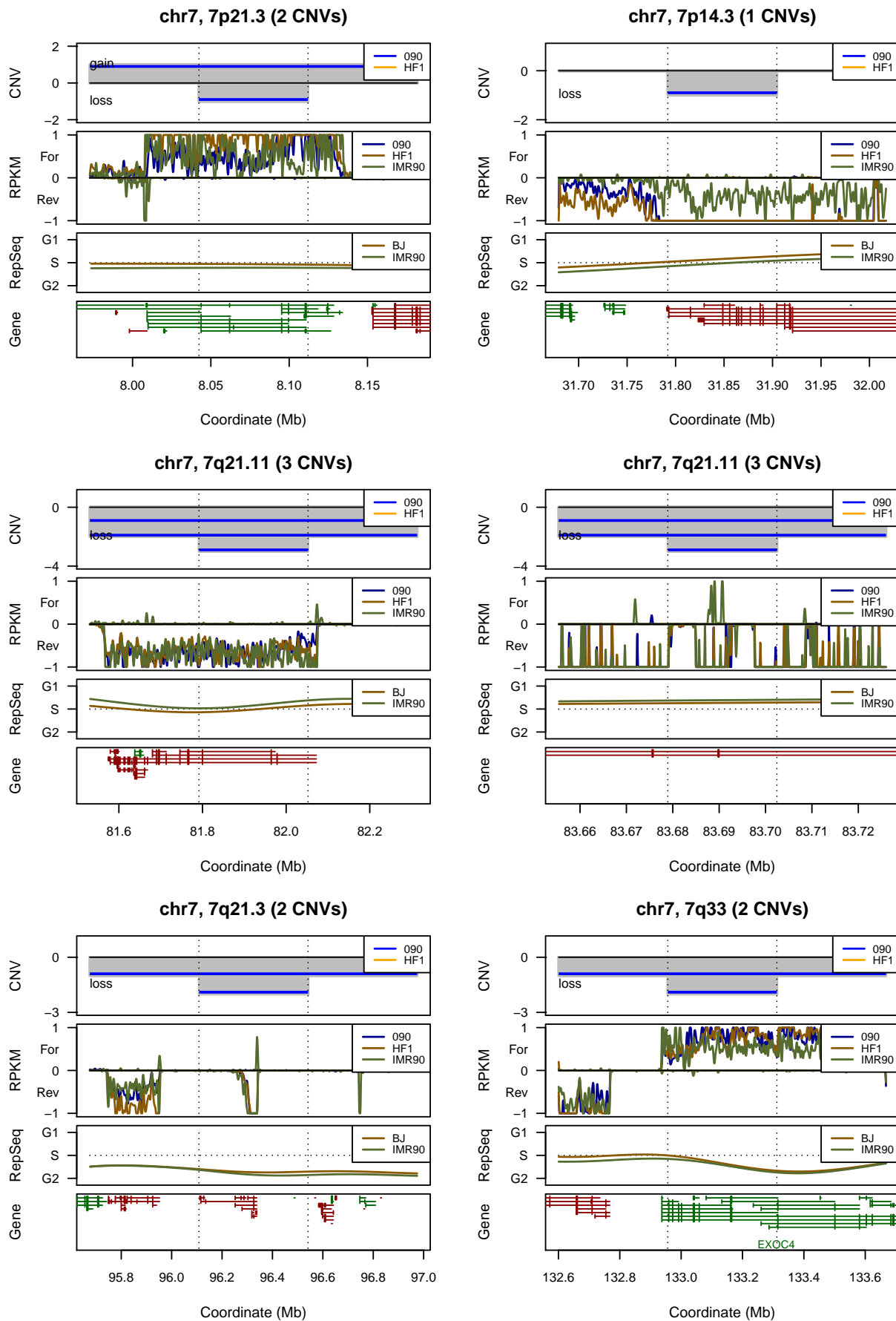

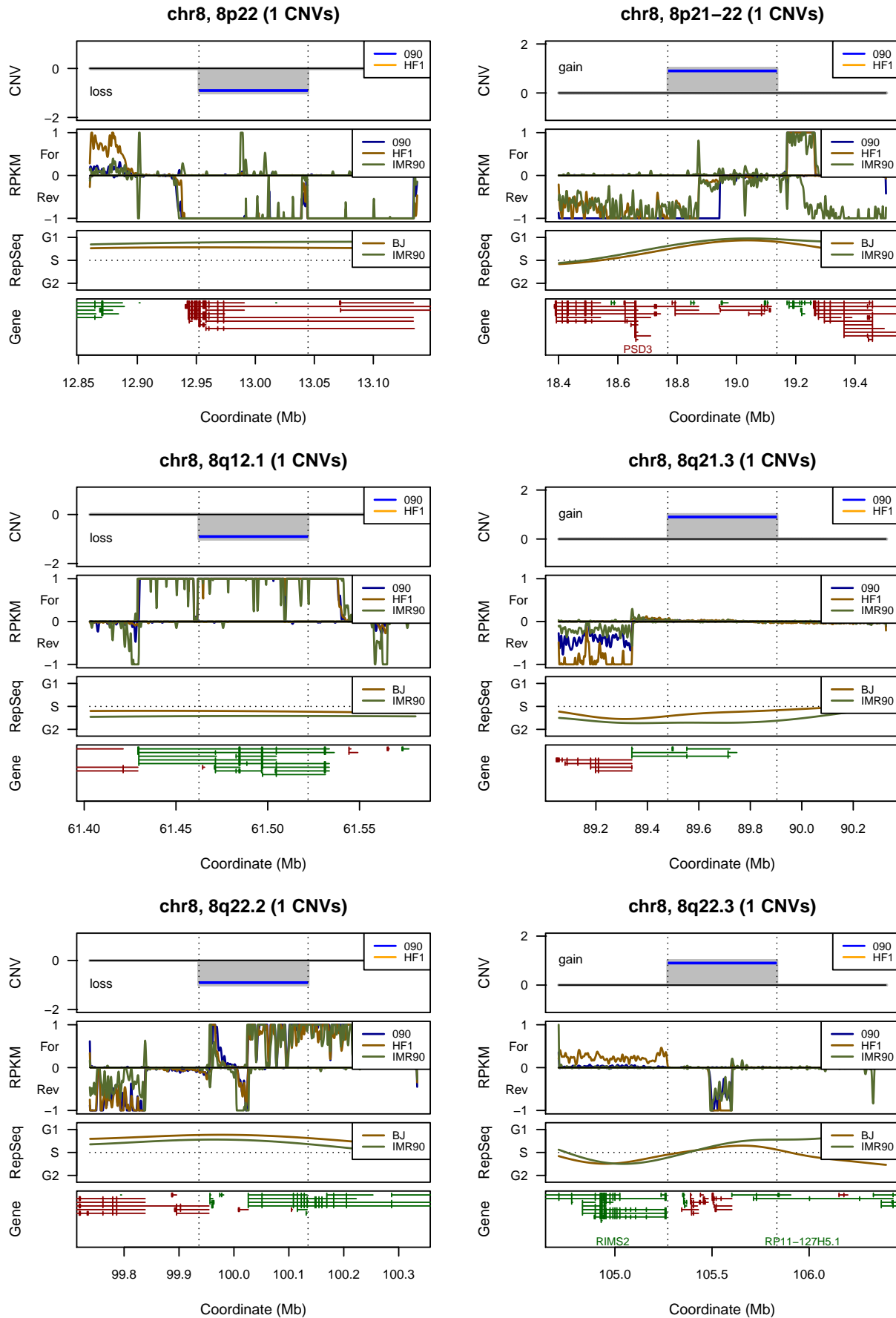

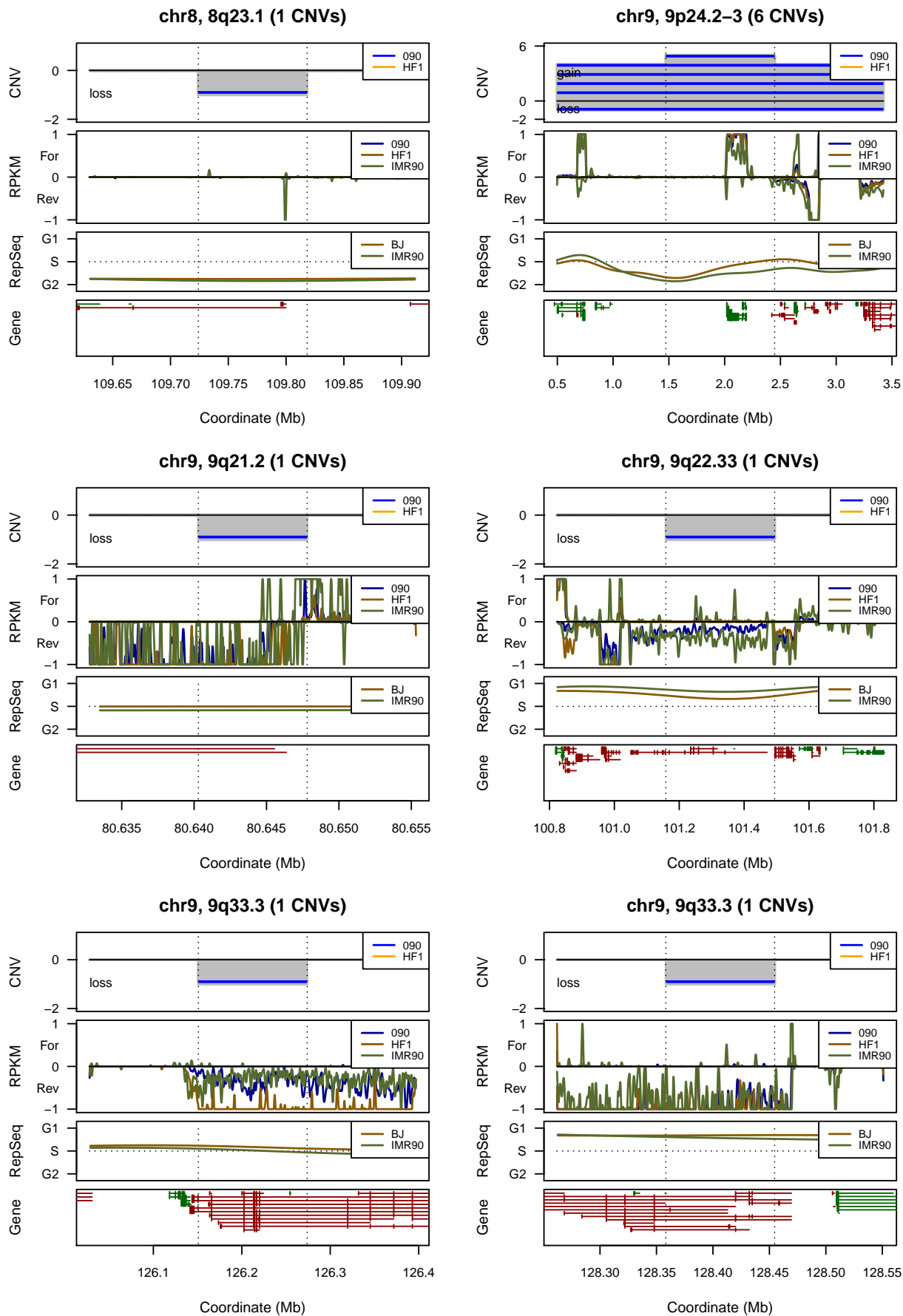

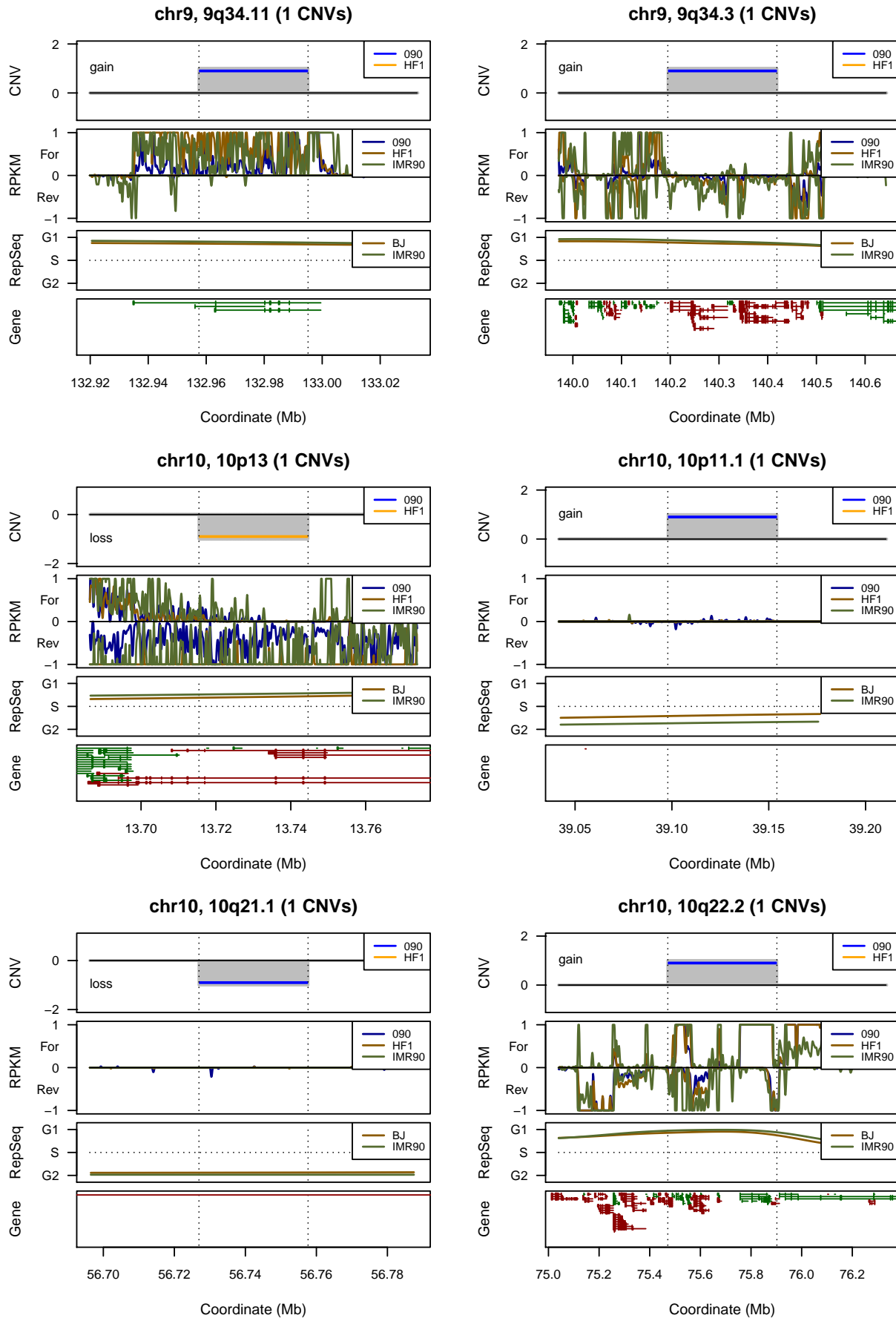

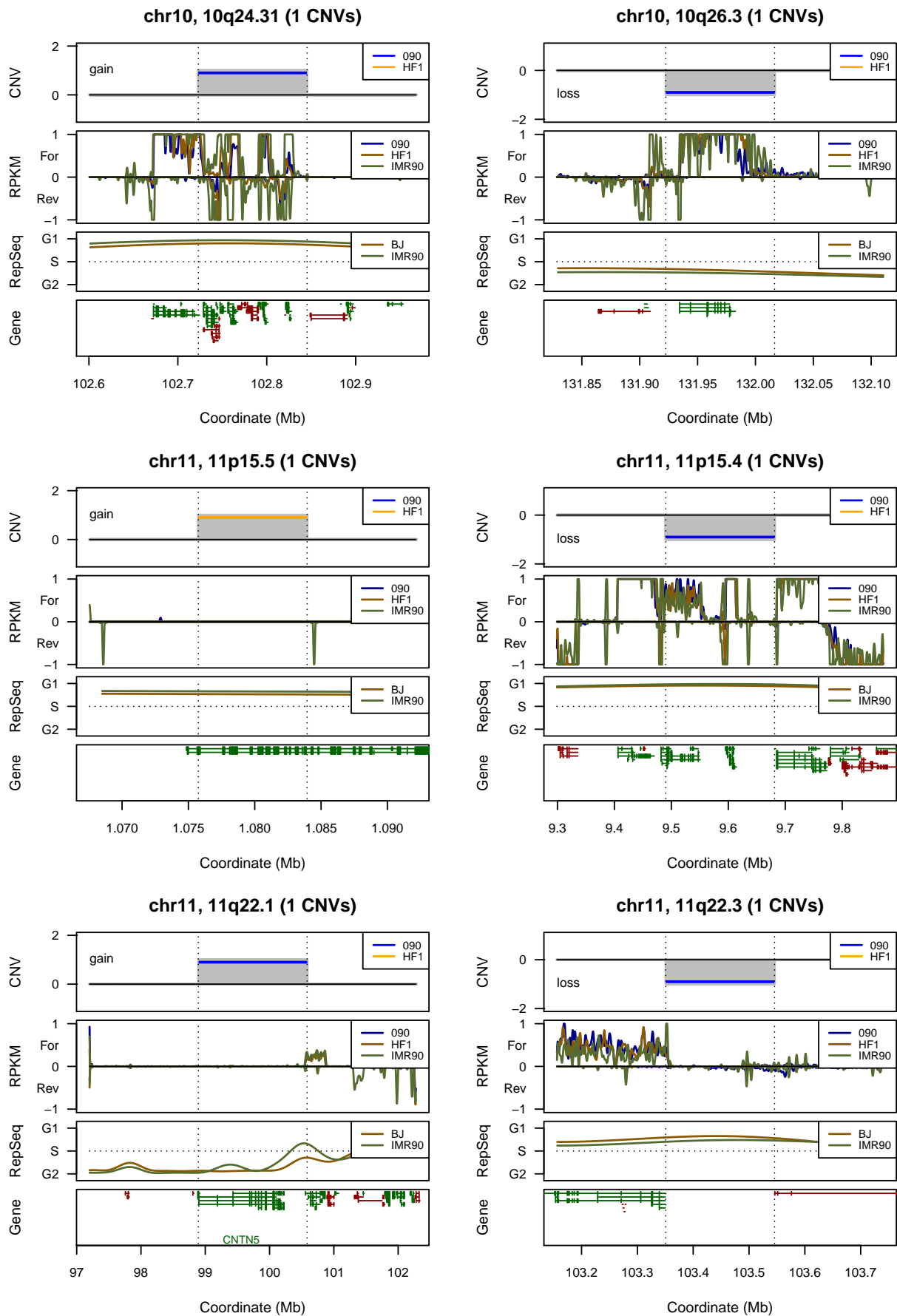

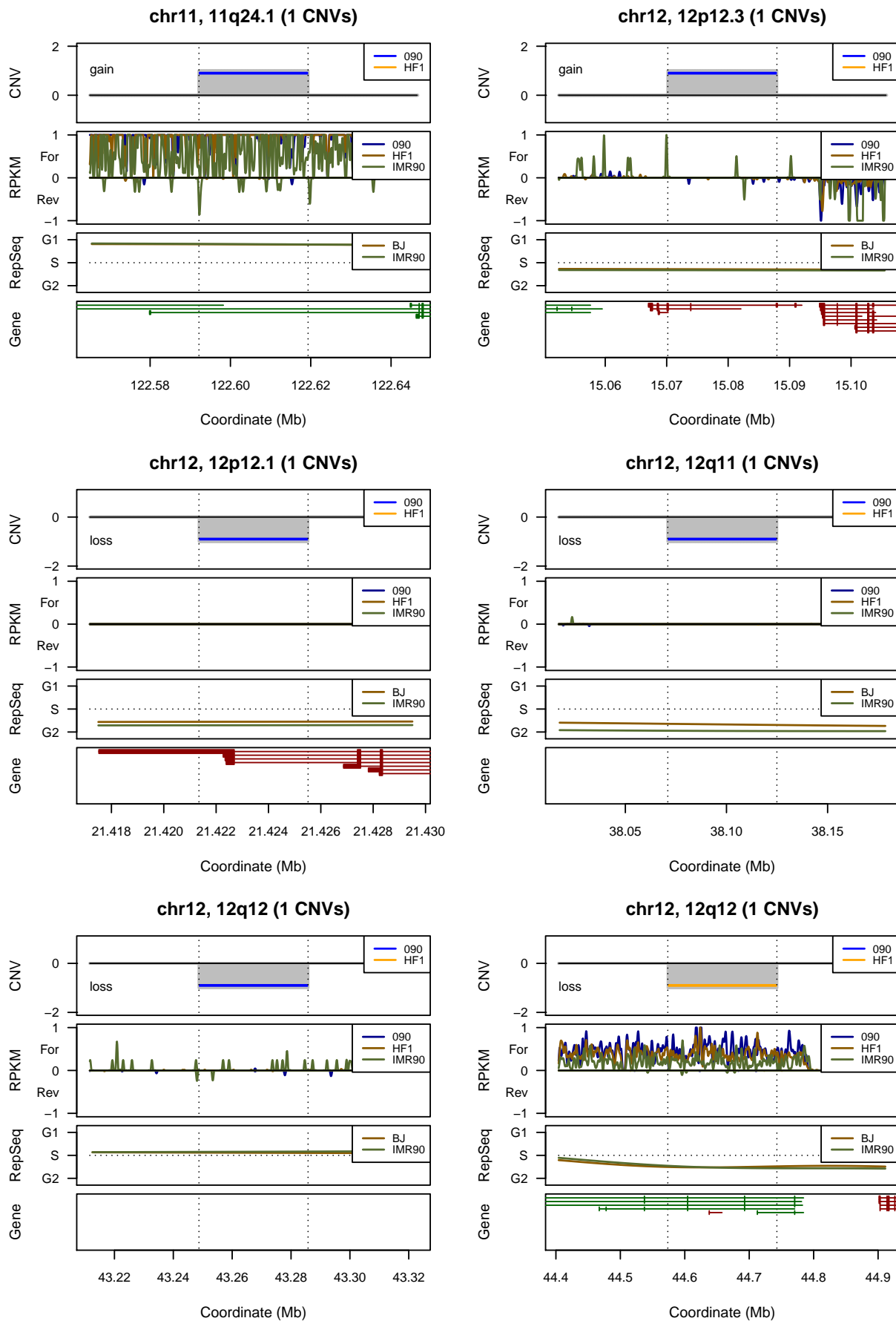

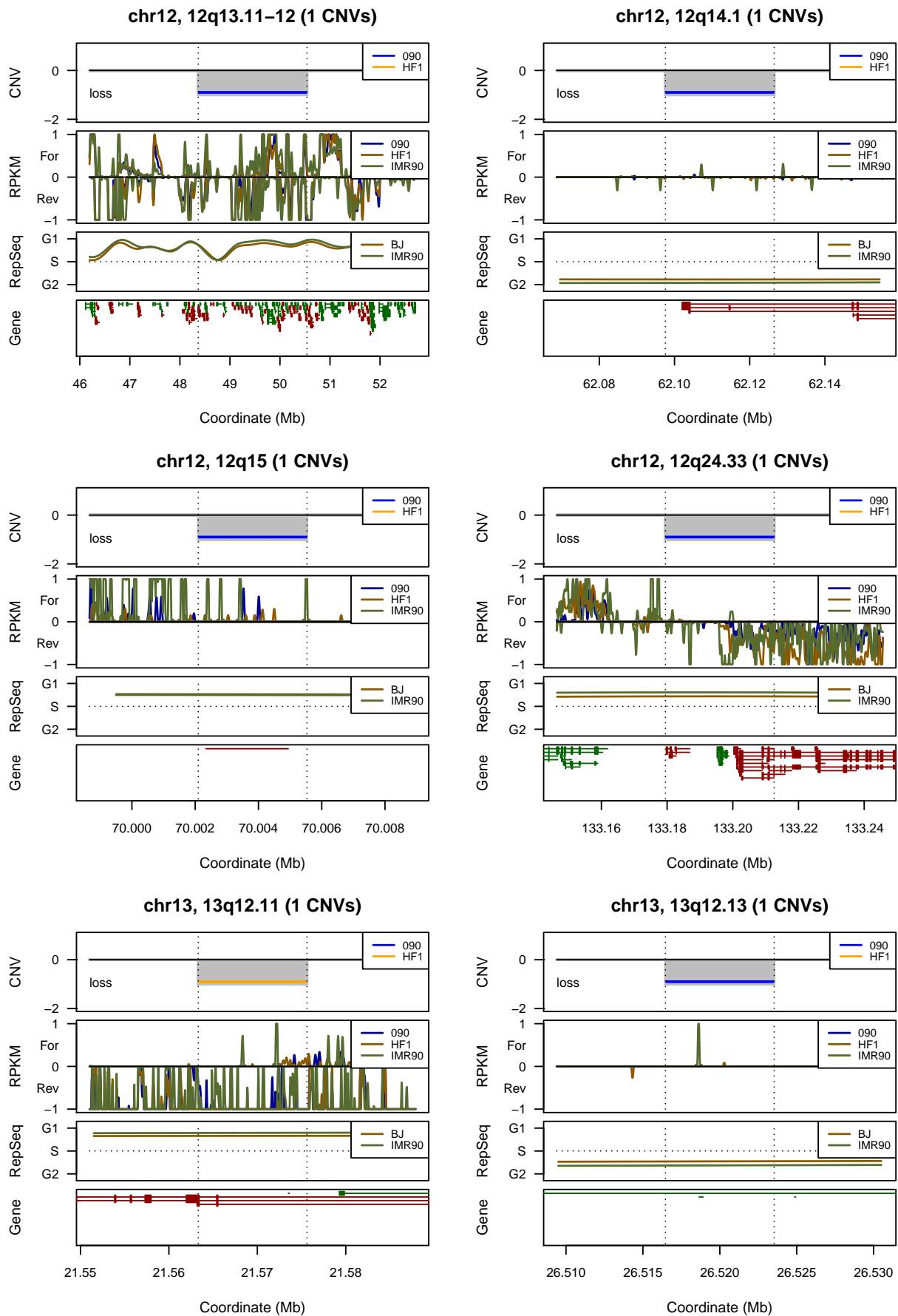

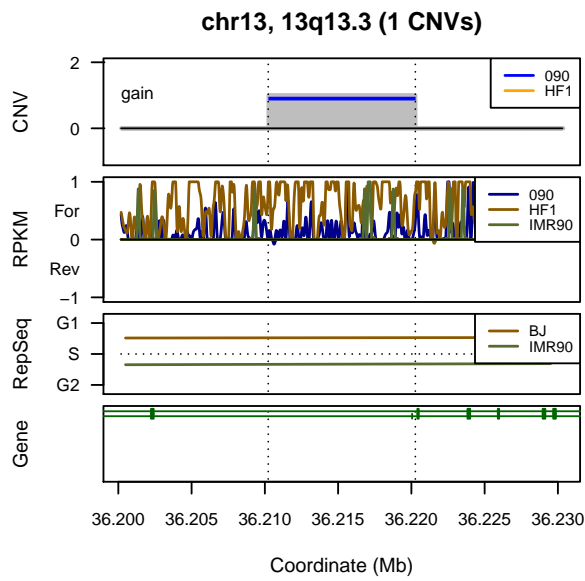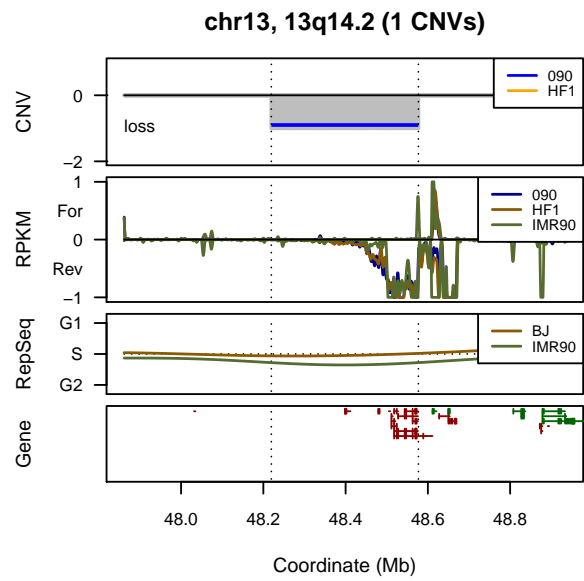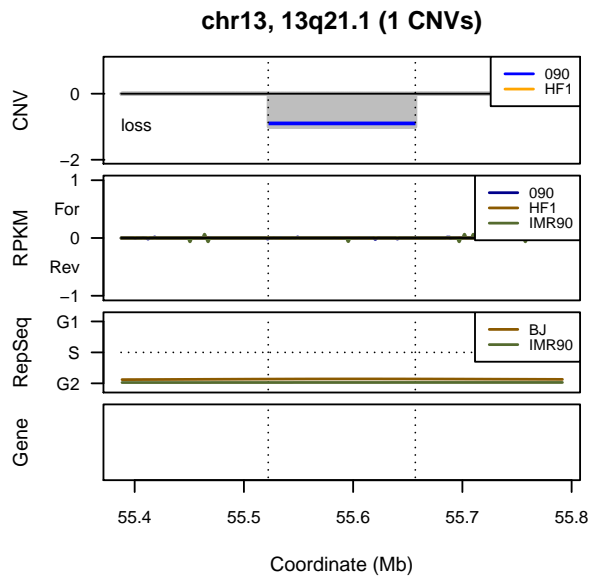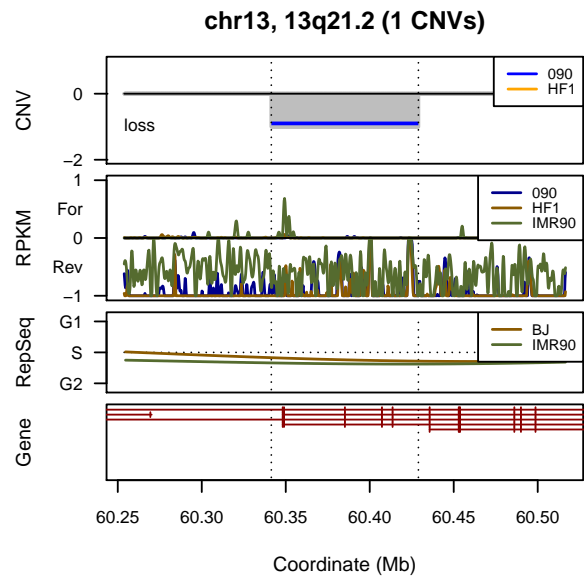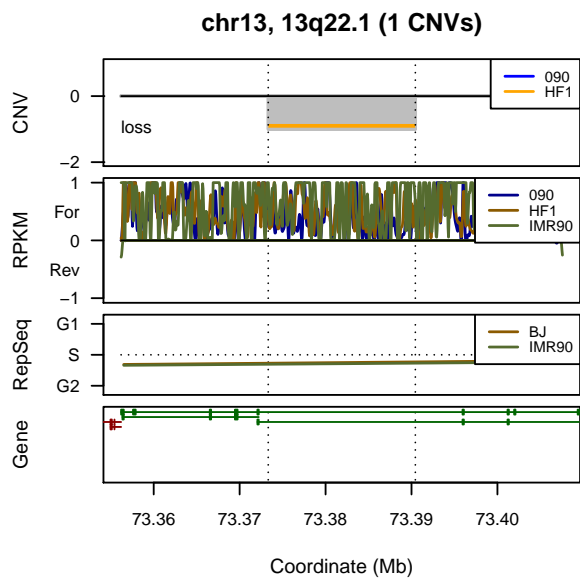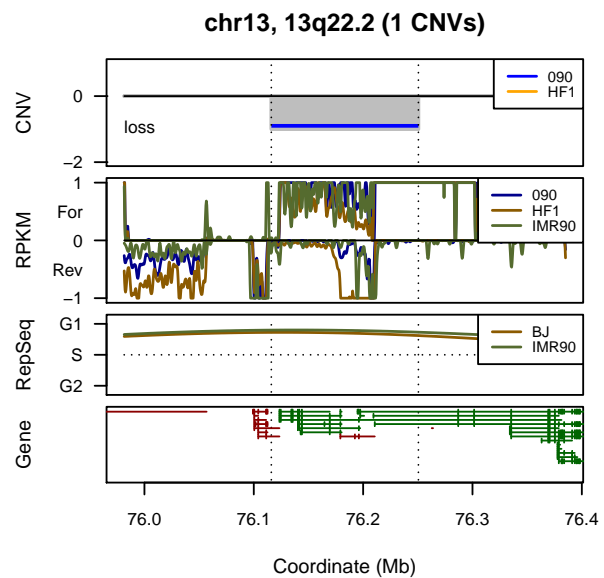

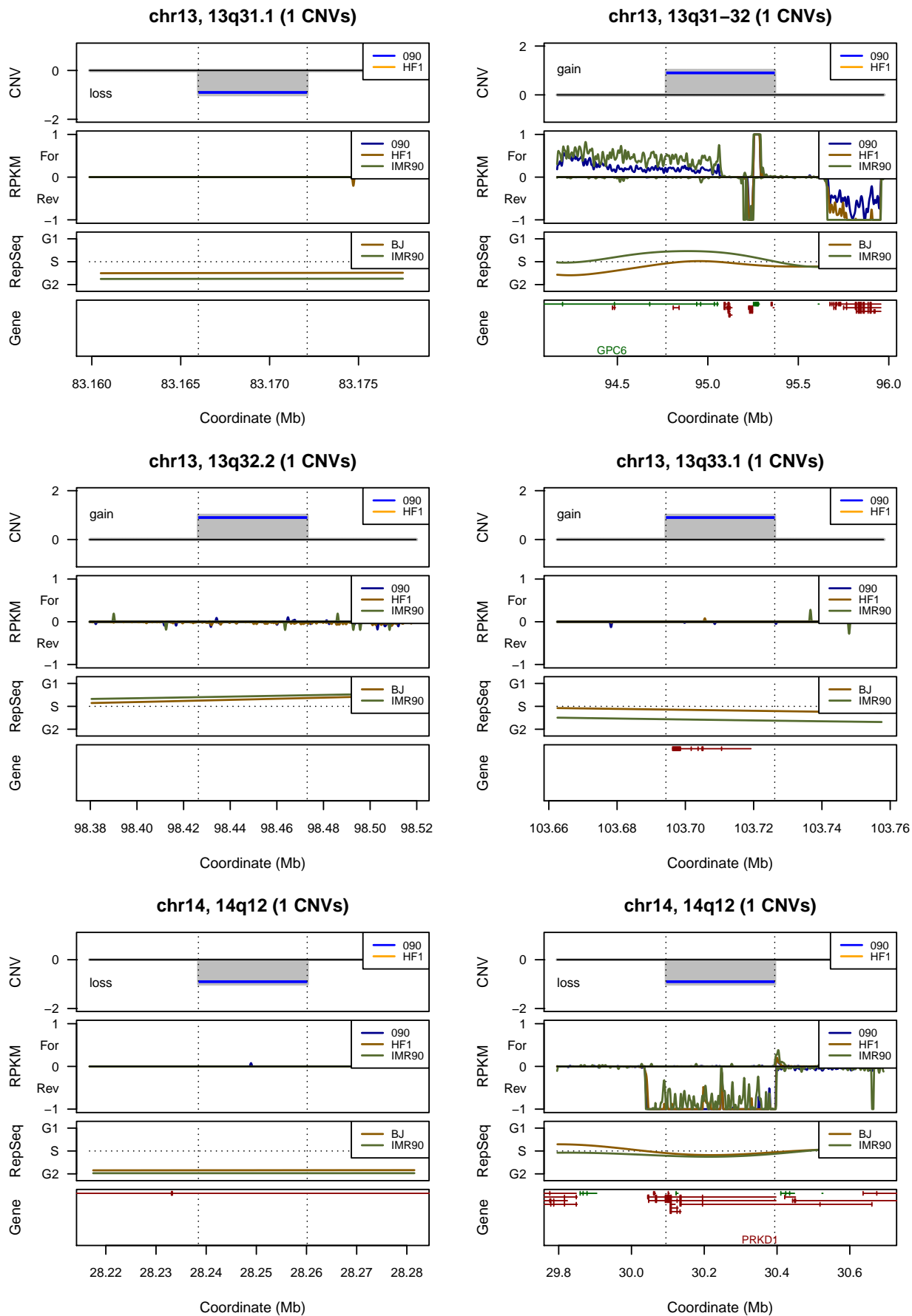

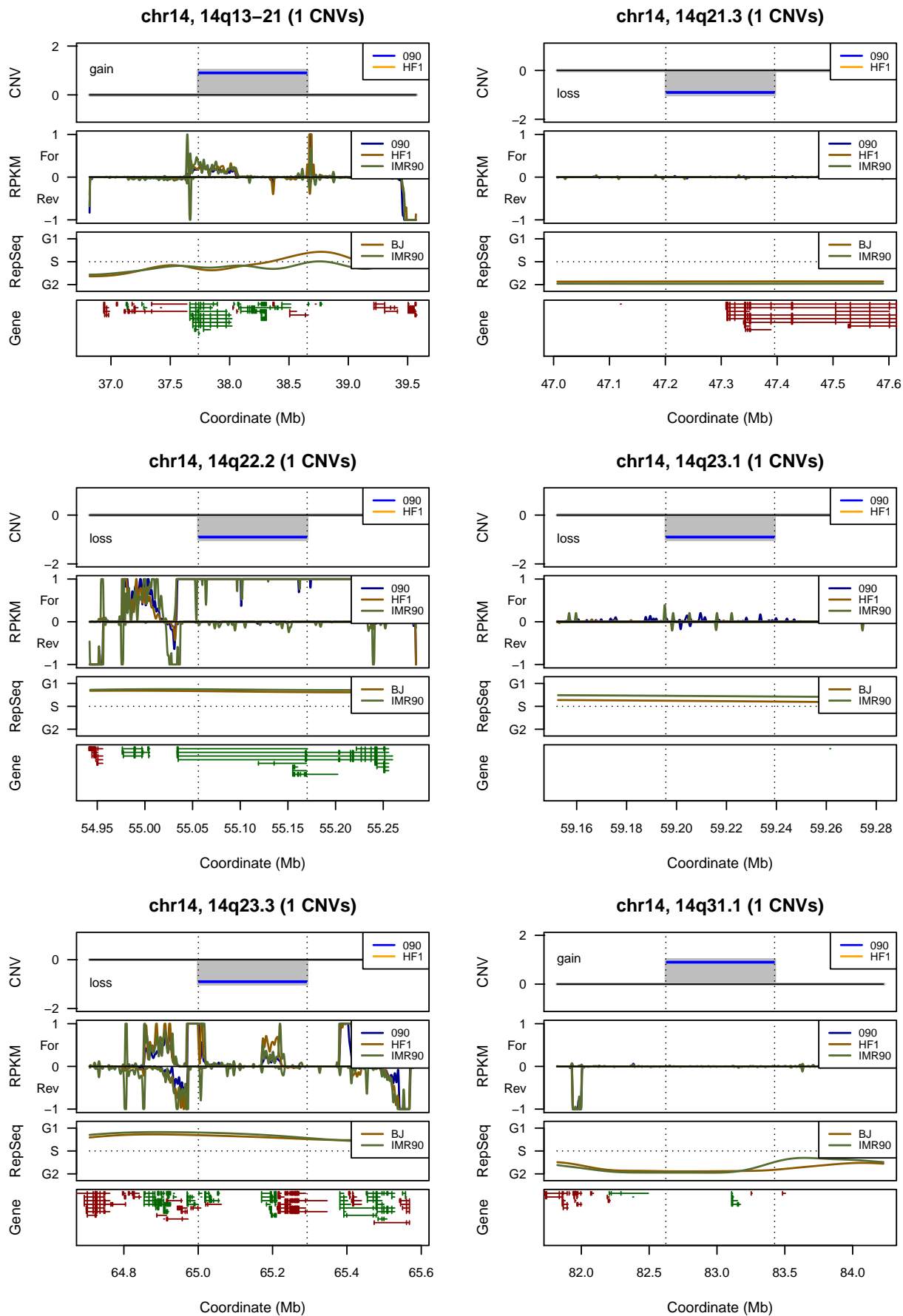

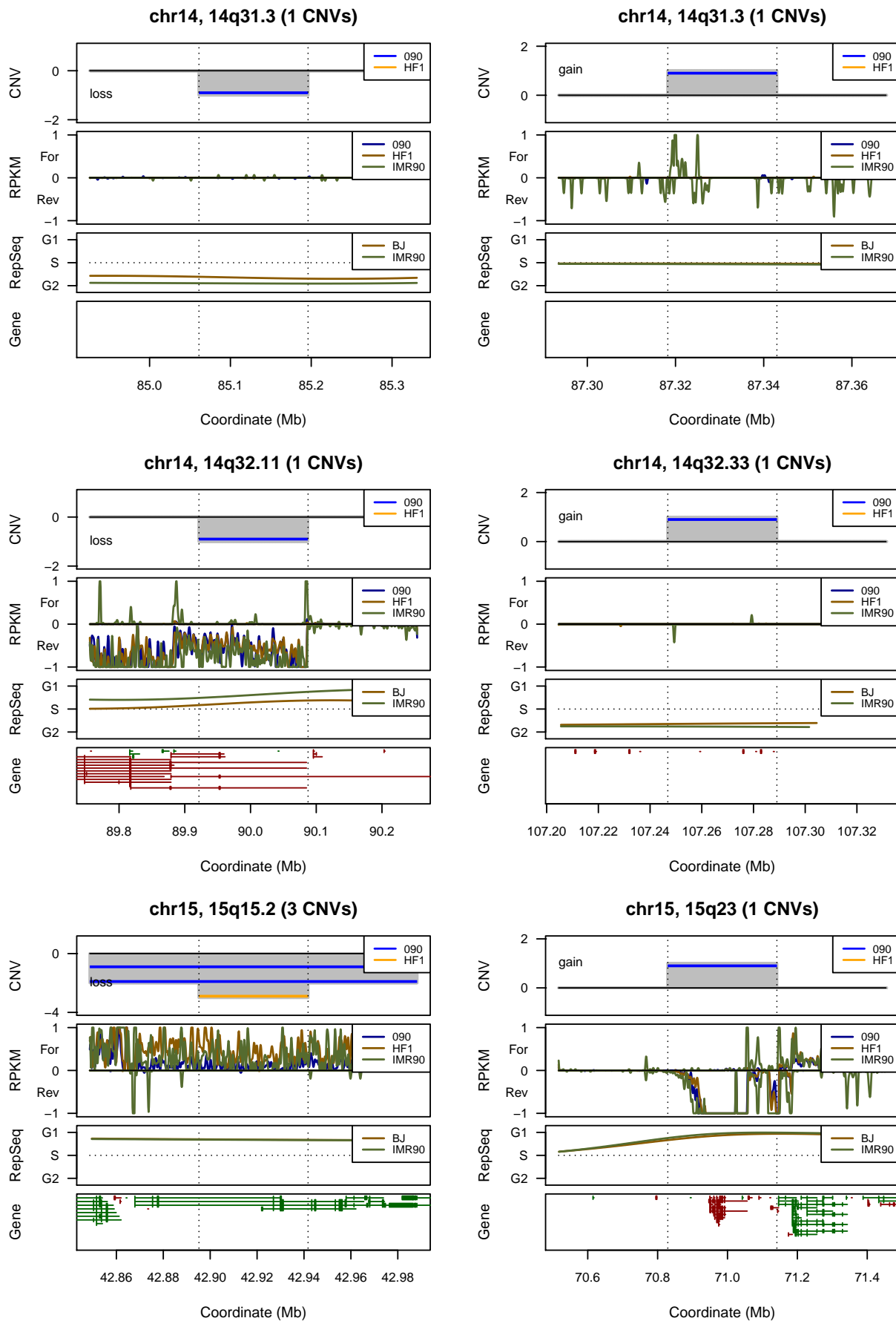

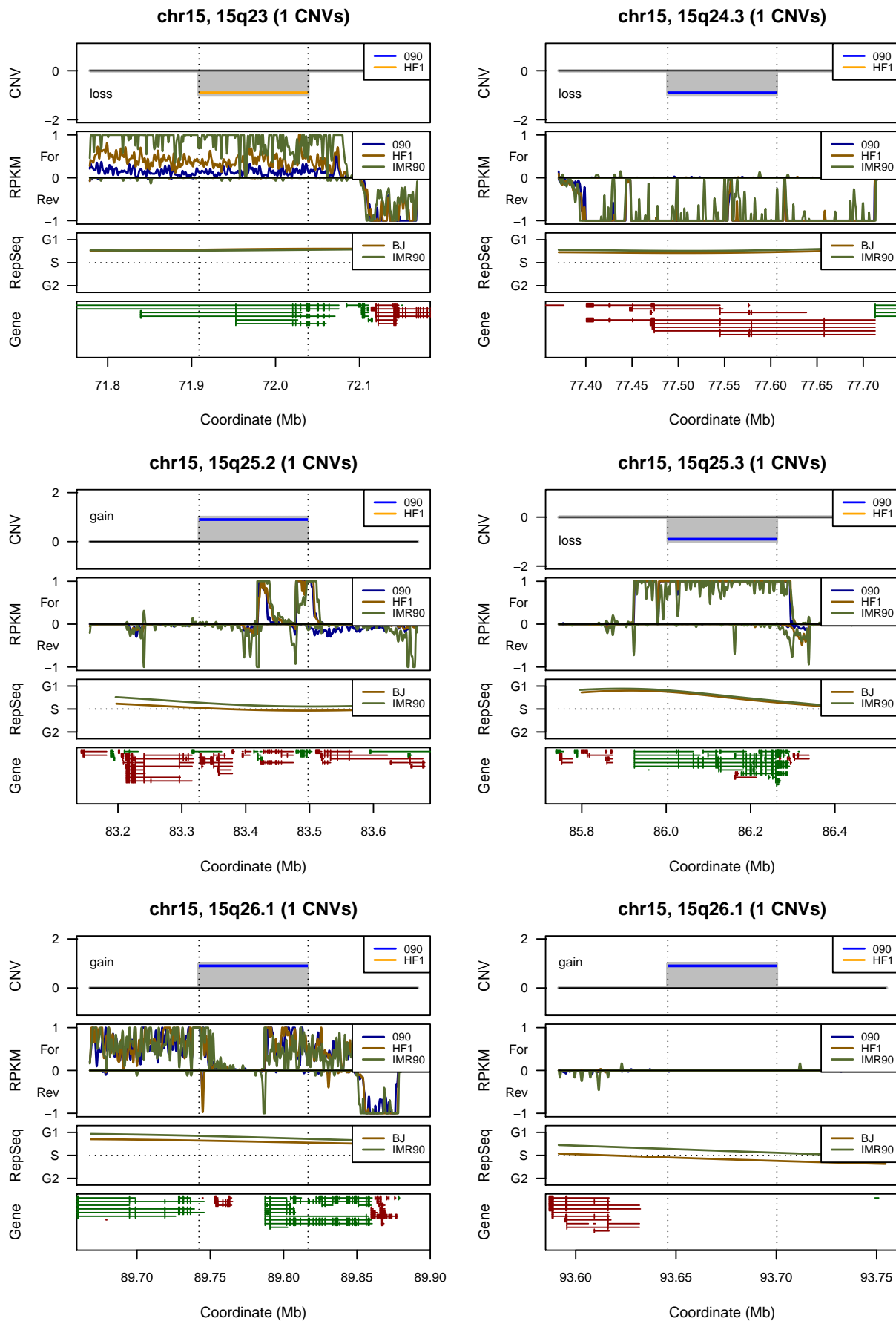

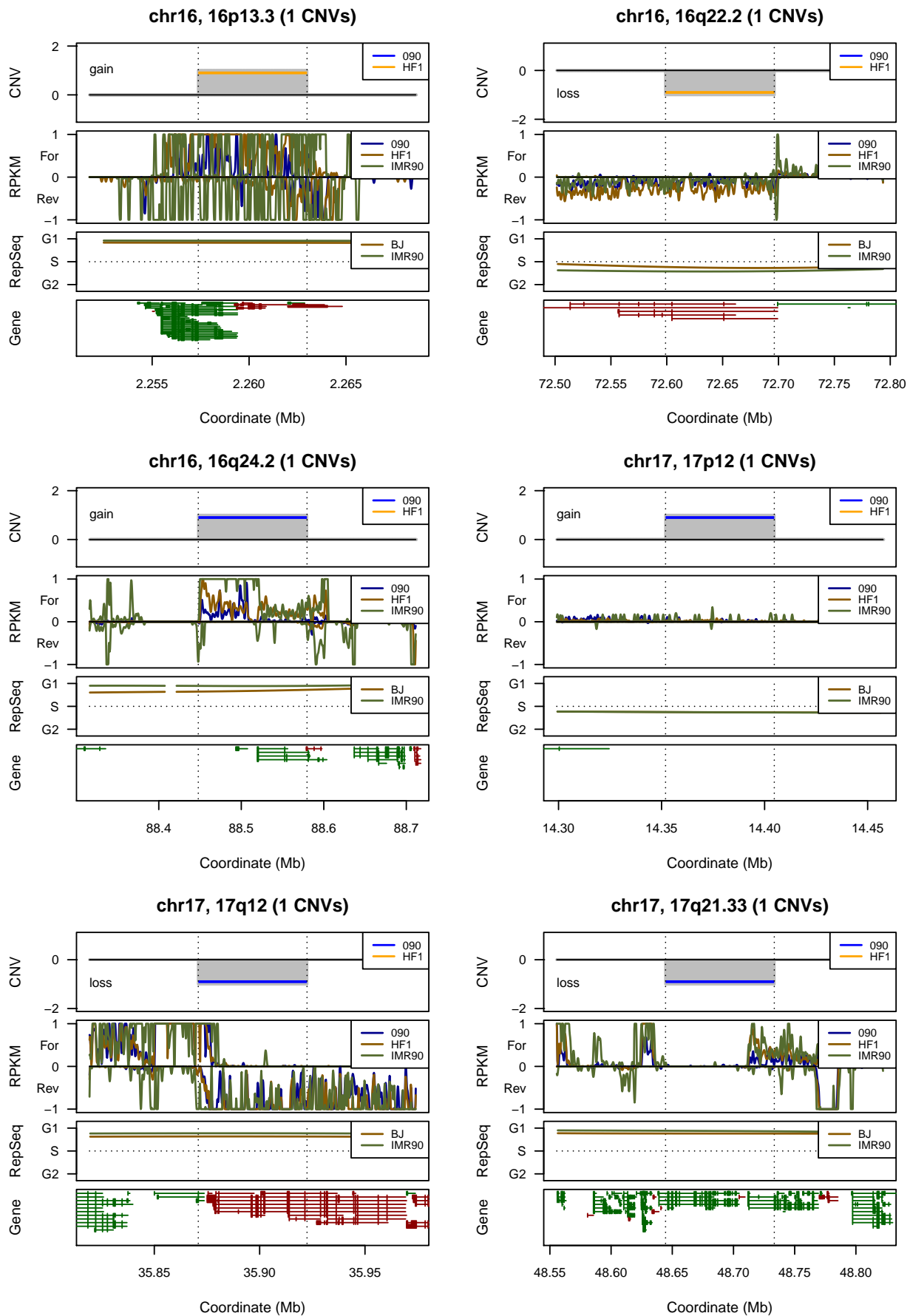

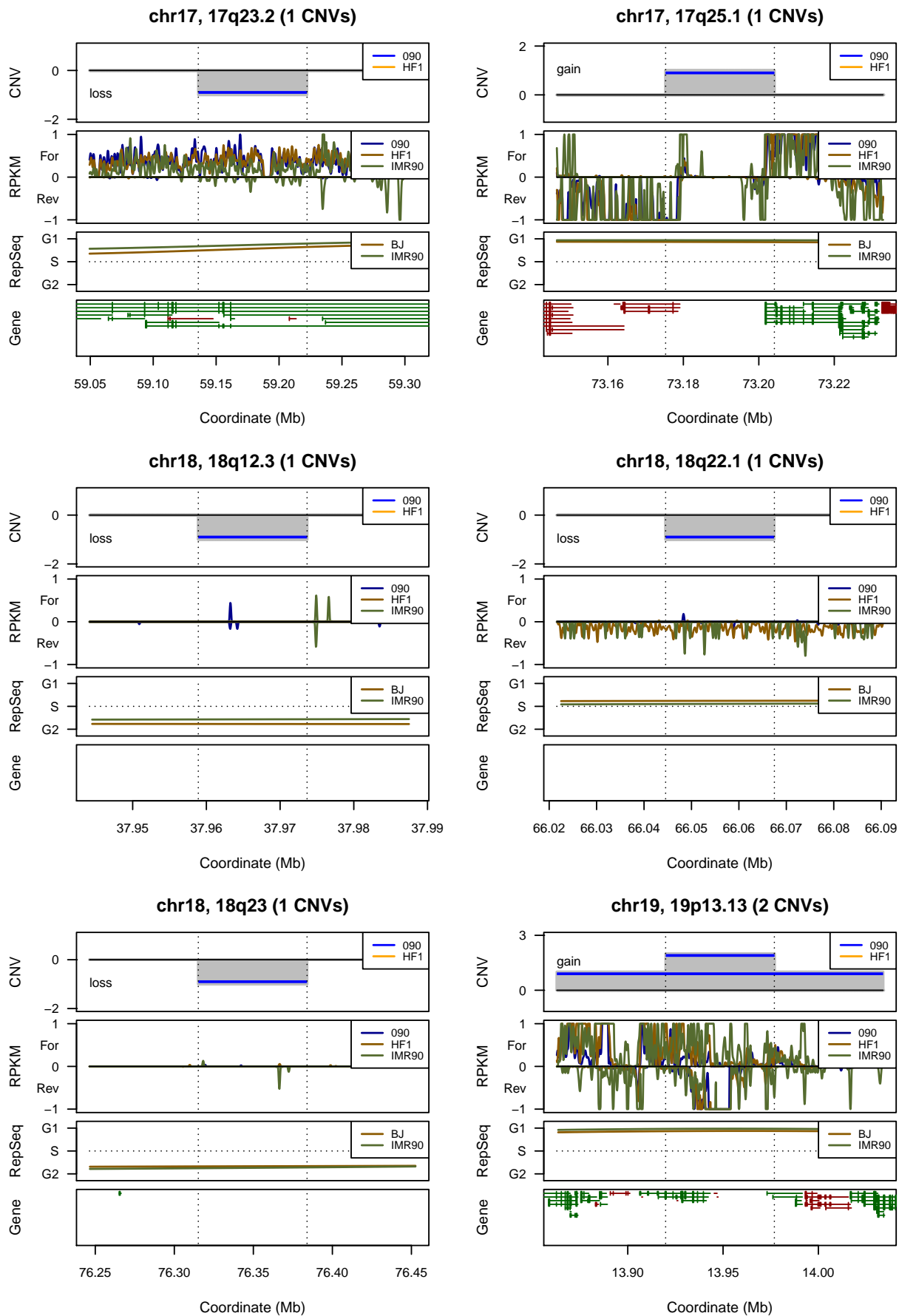

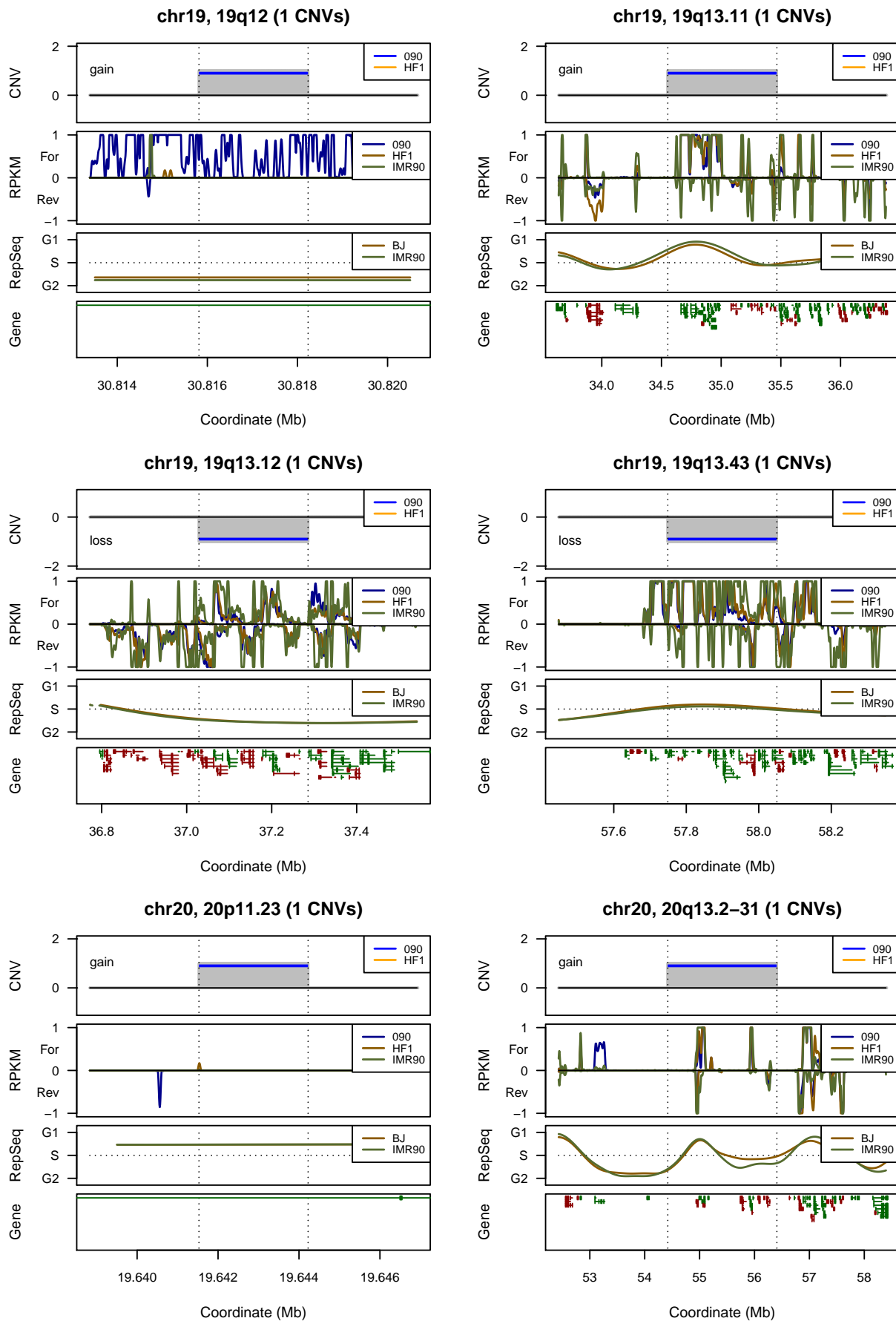

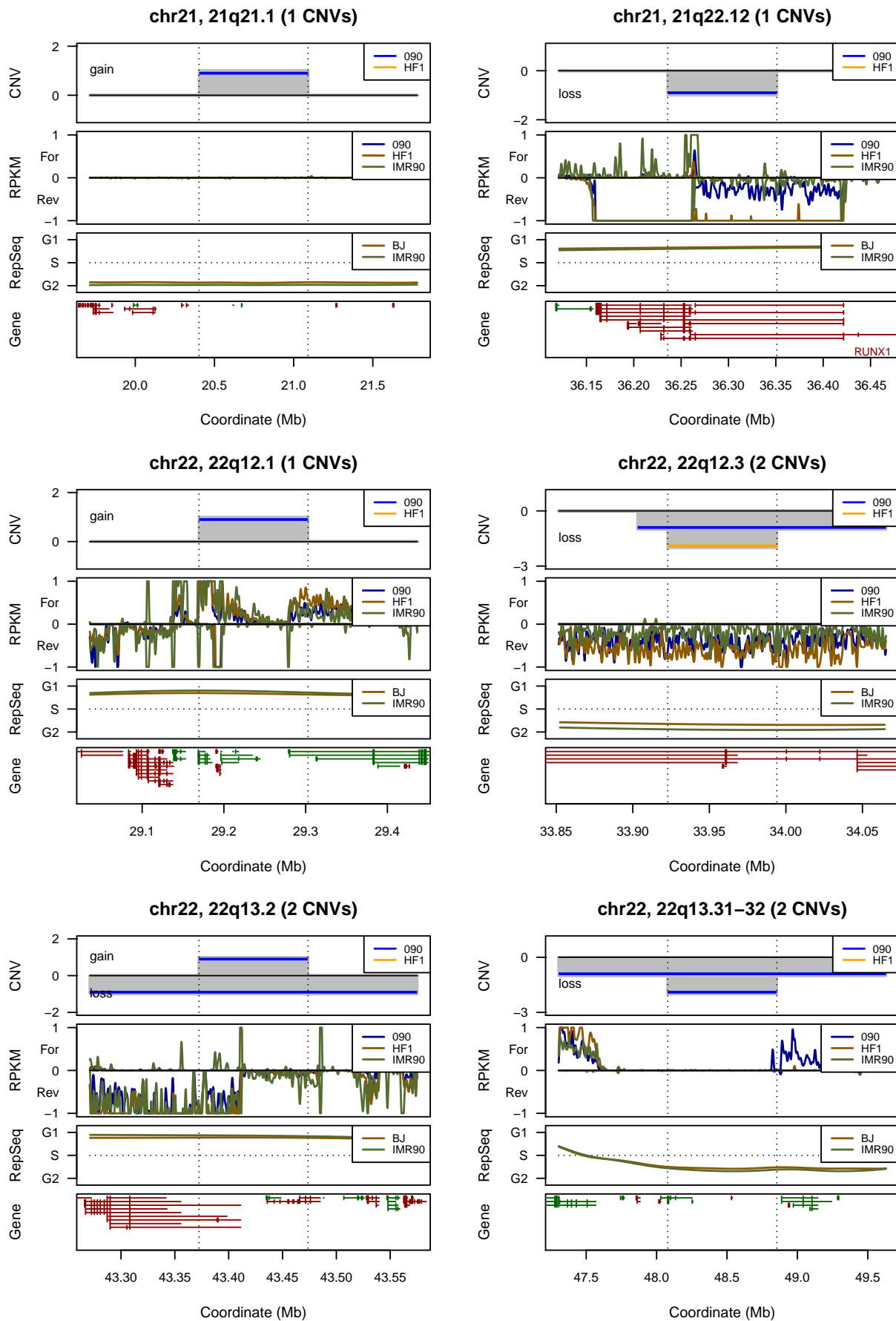

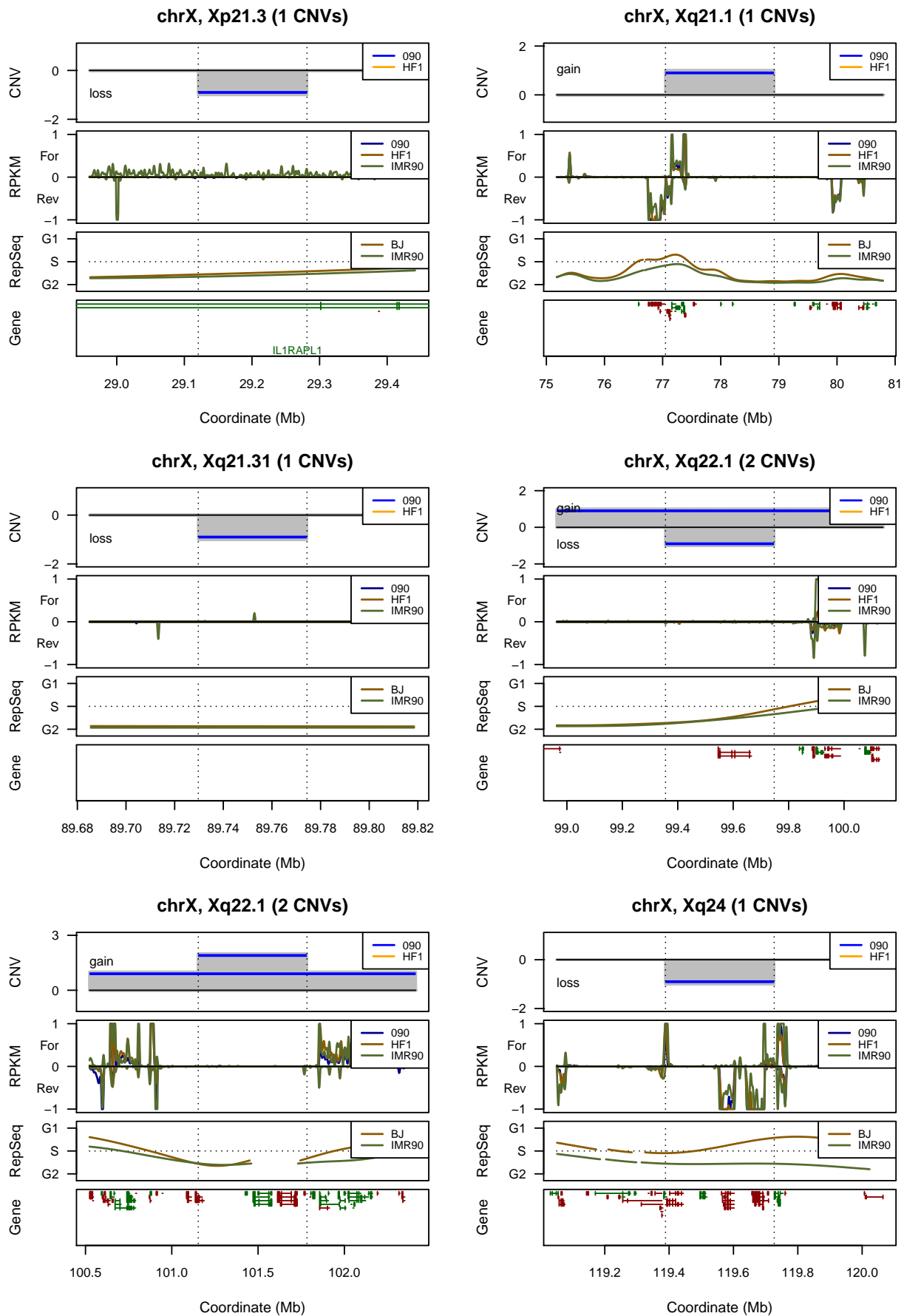

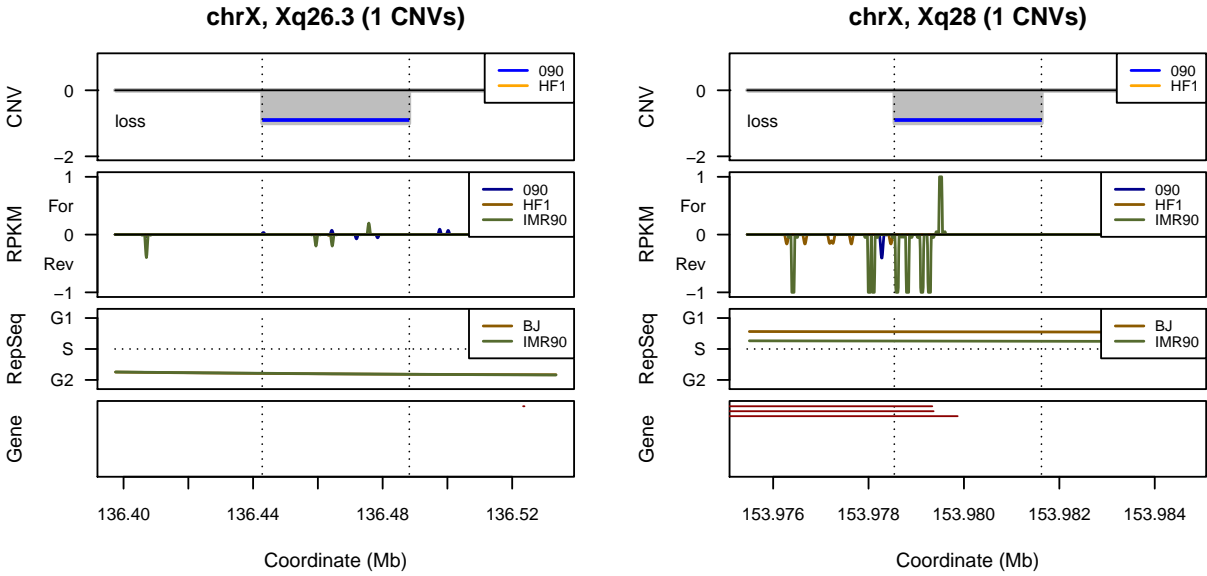

Supplement: Supplemental Material [file supp_gr.177121.114_Figure_S1G.pdf]

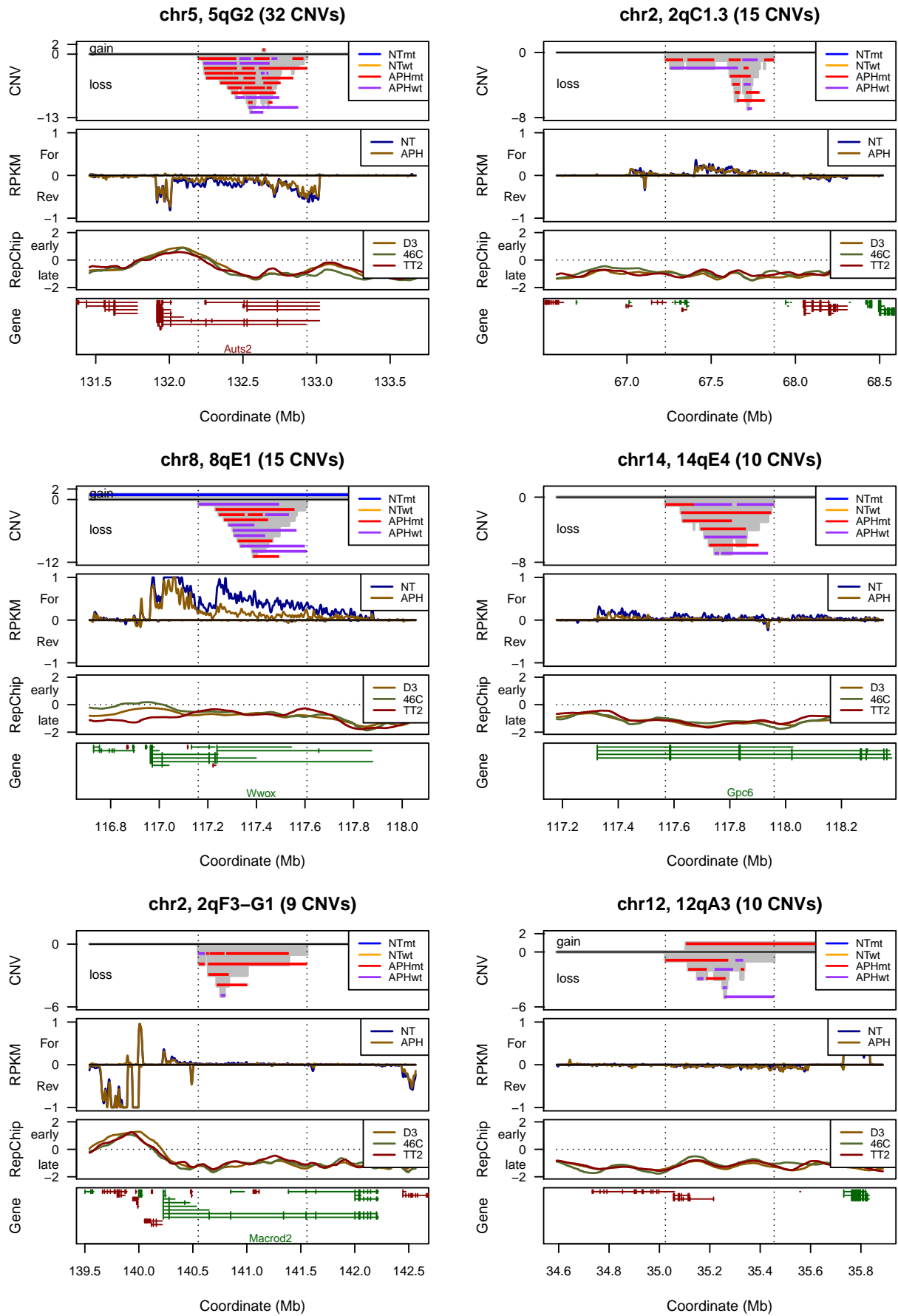

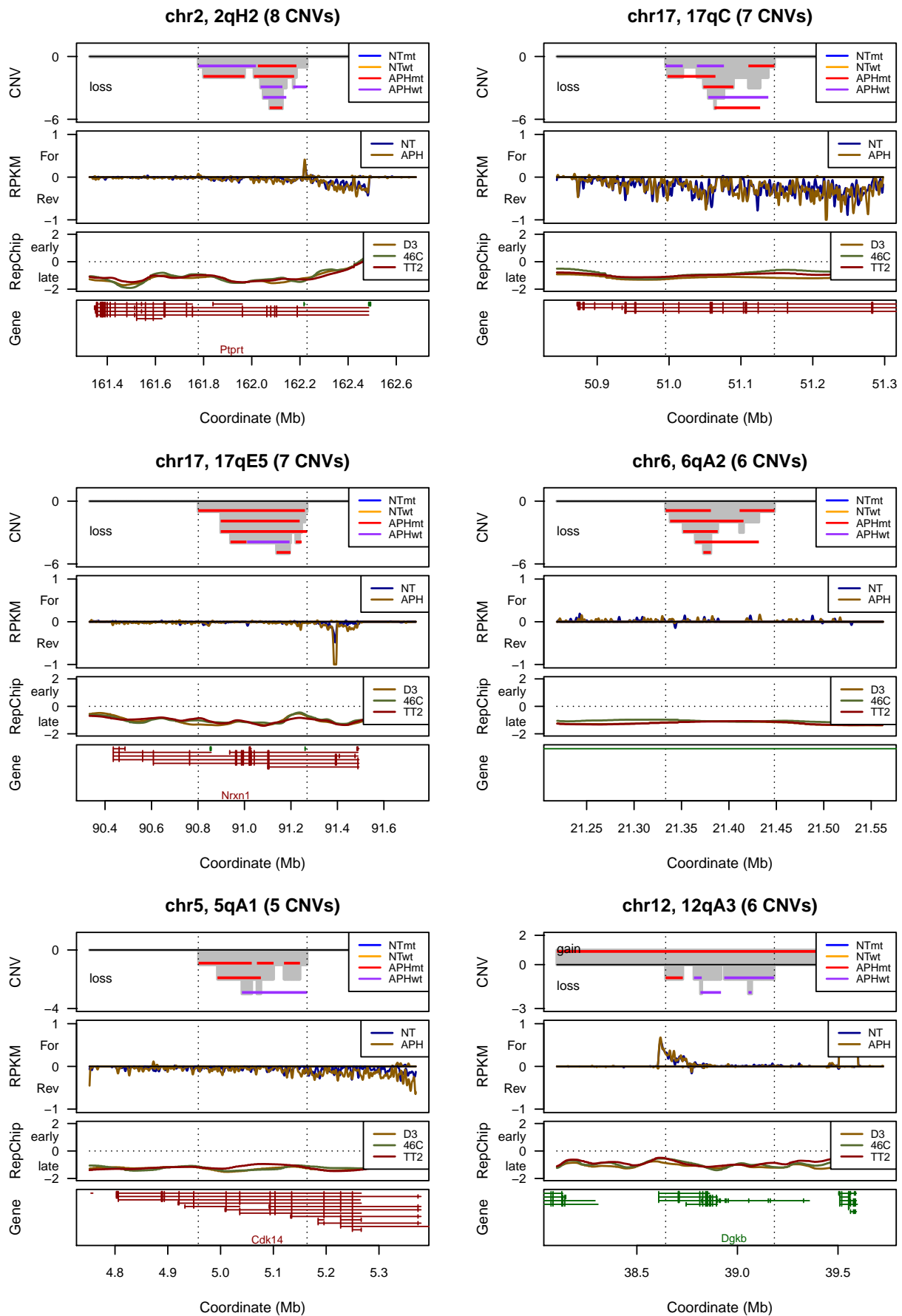

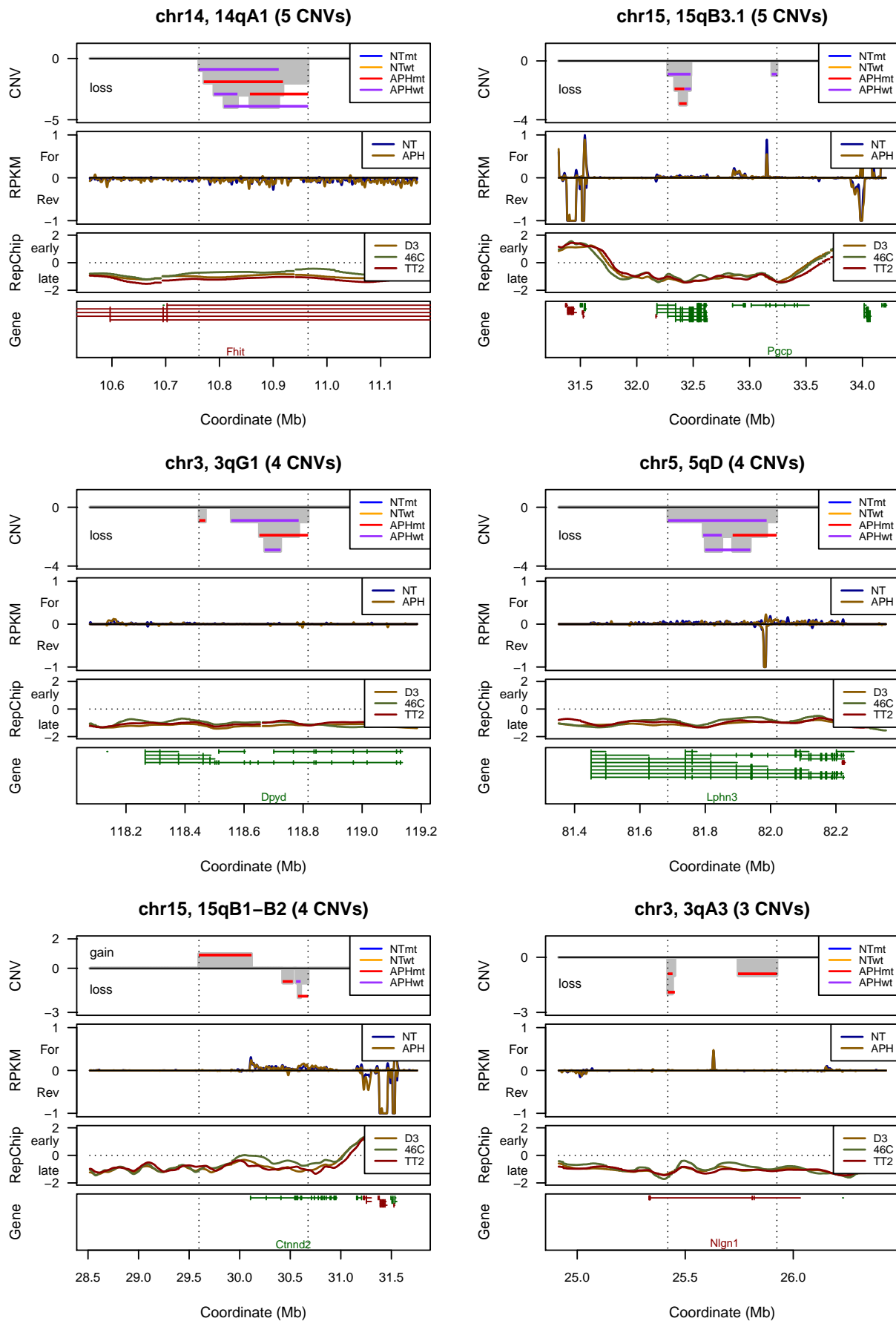

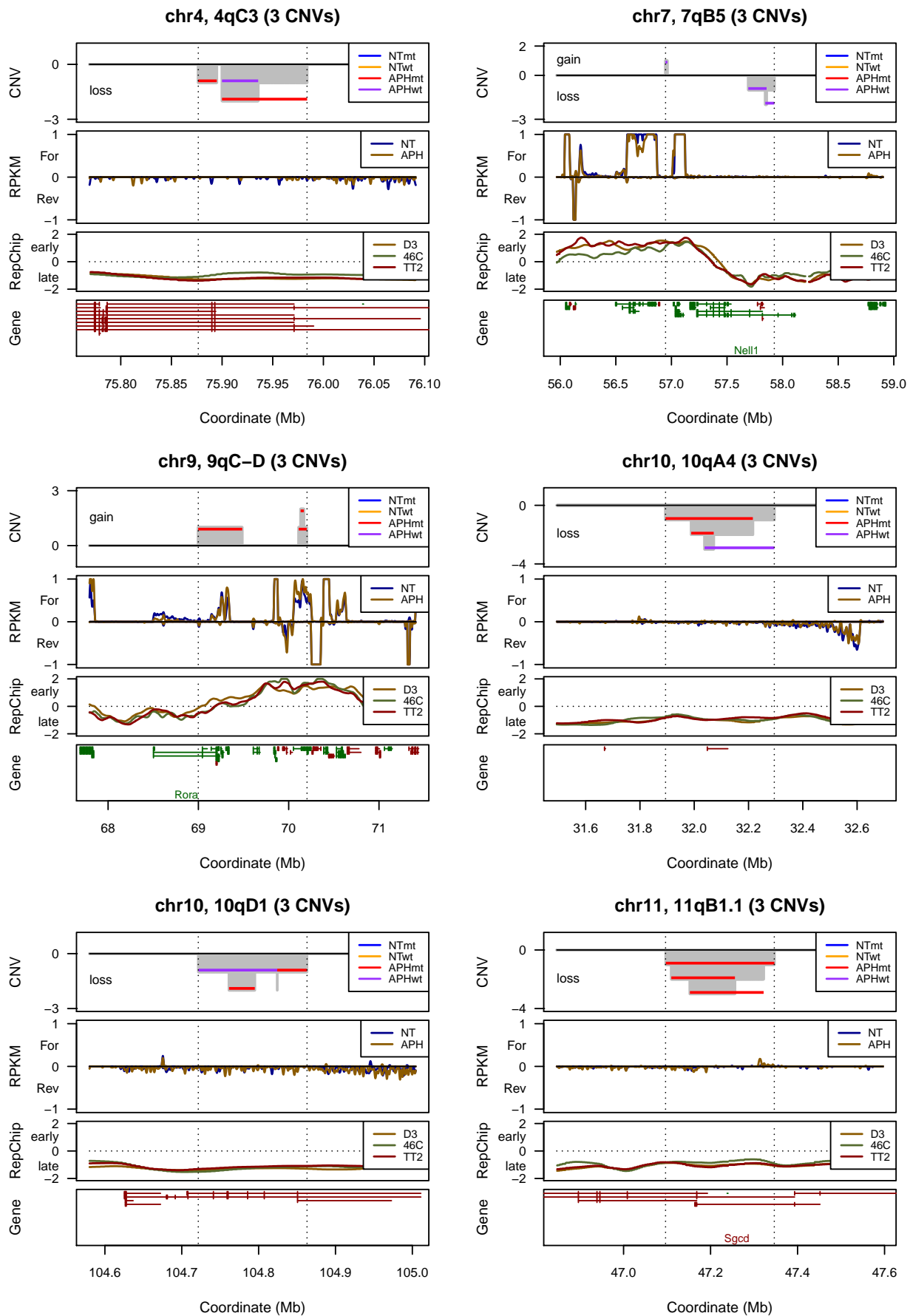

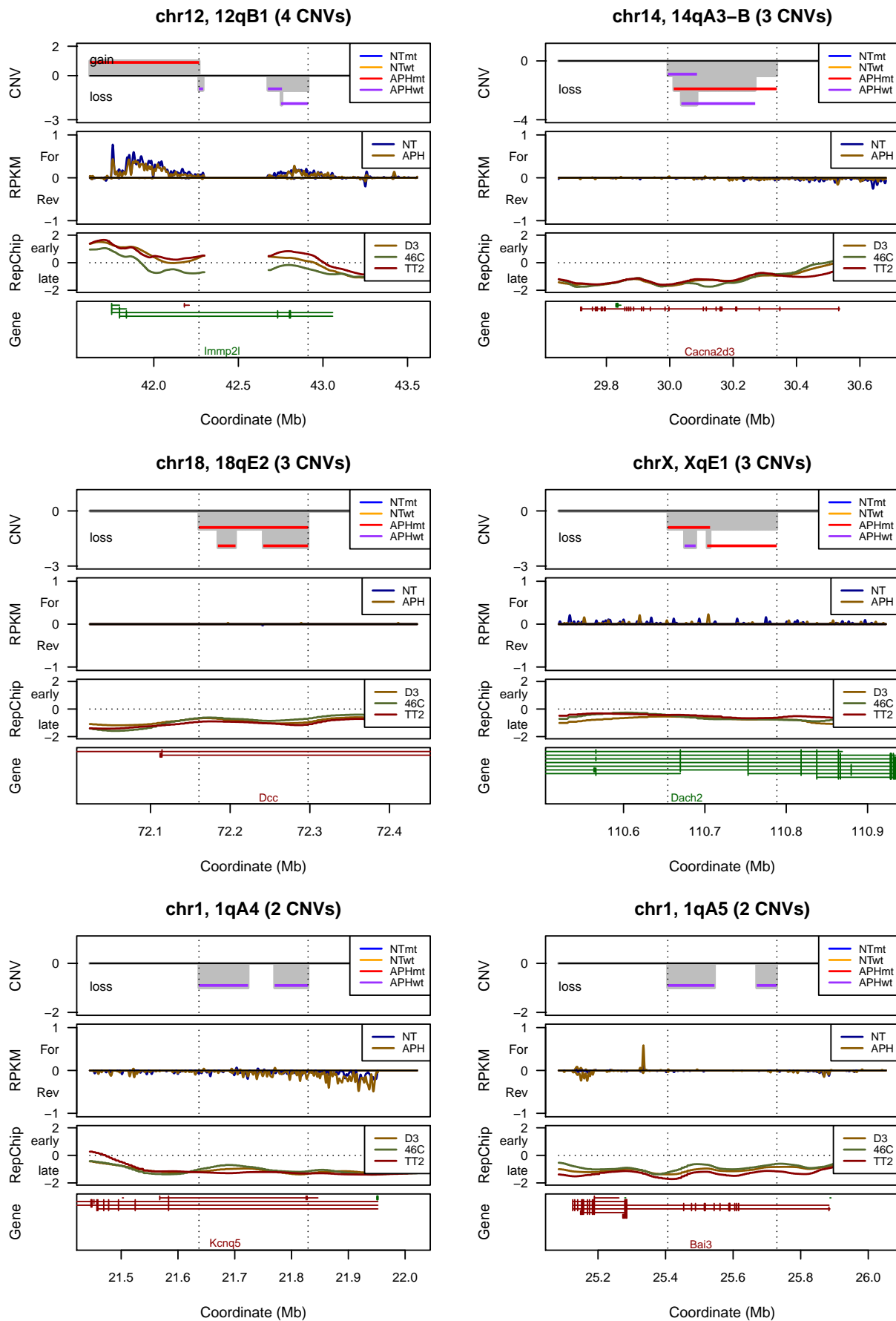

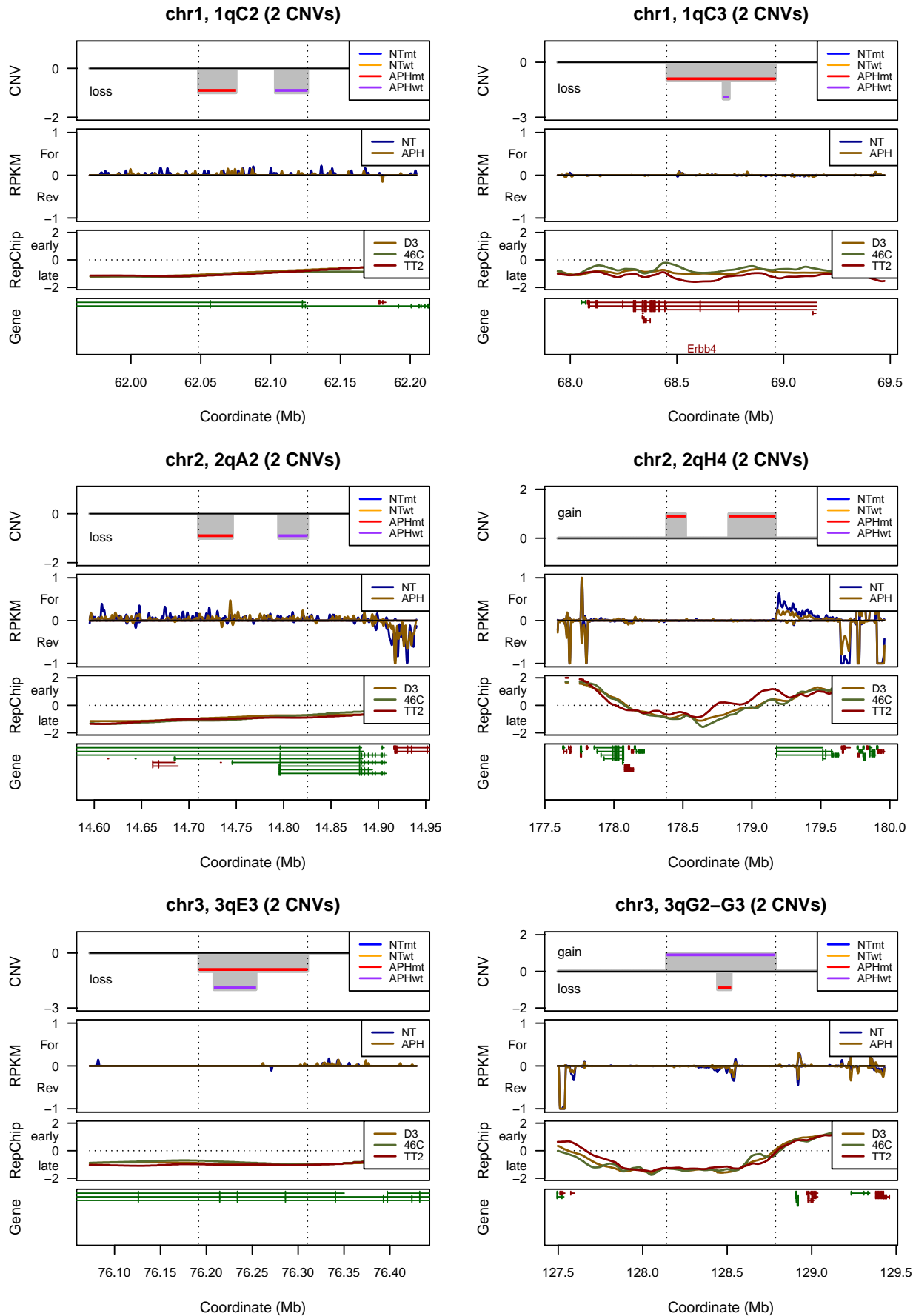

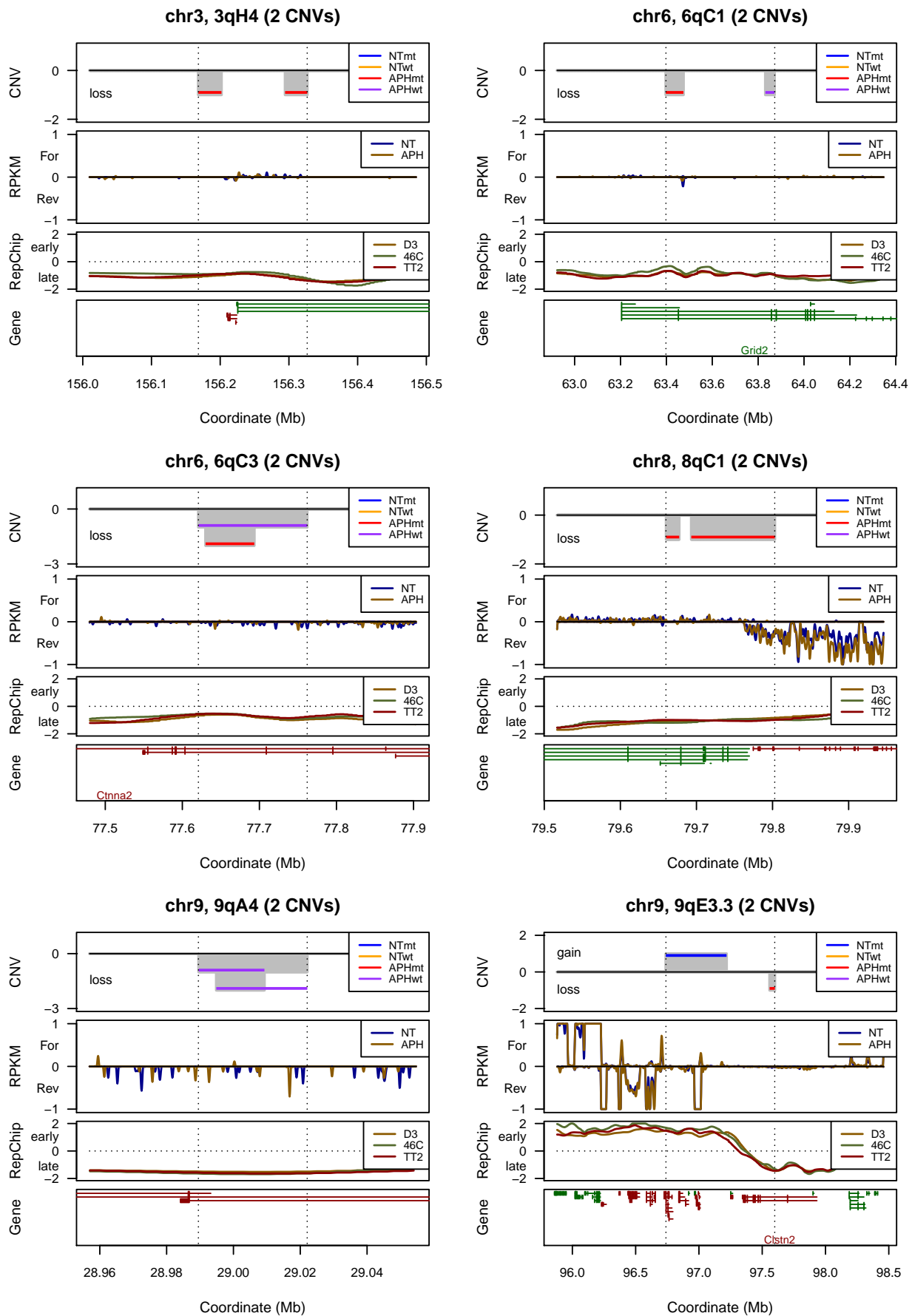

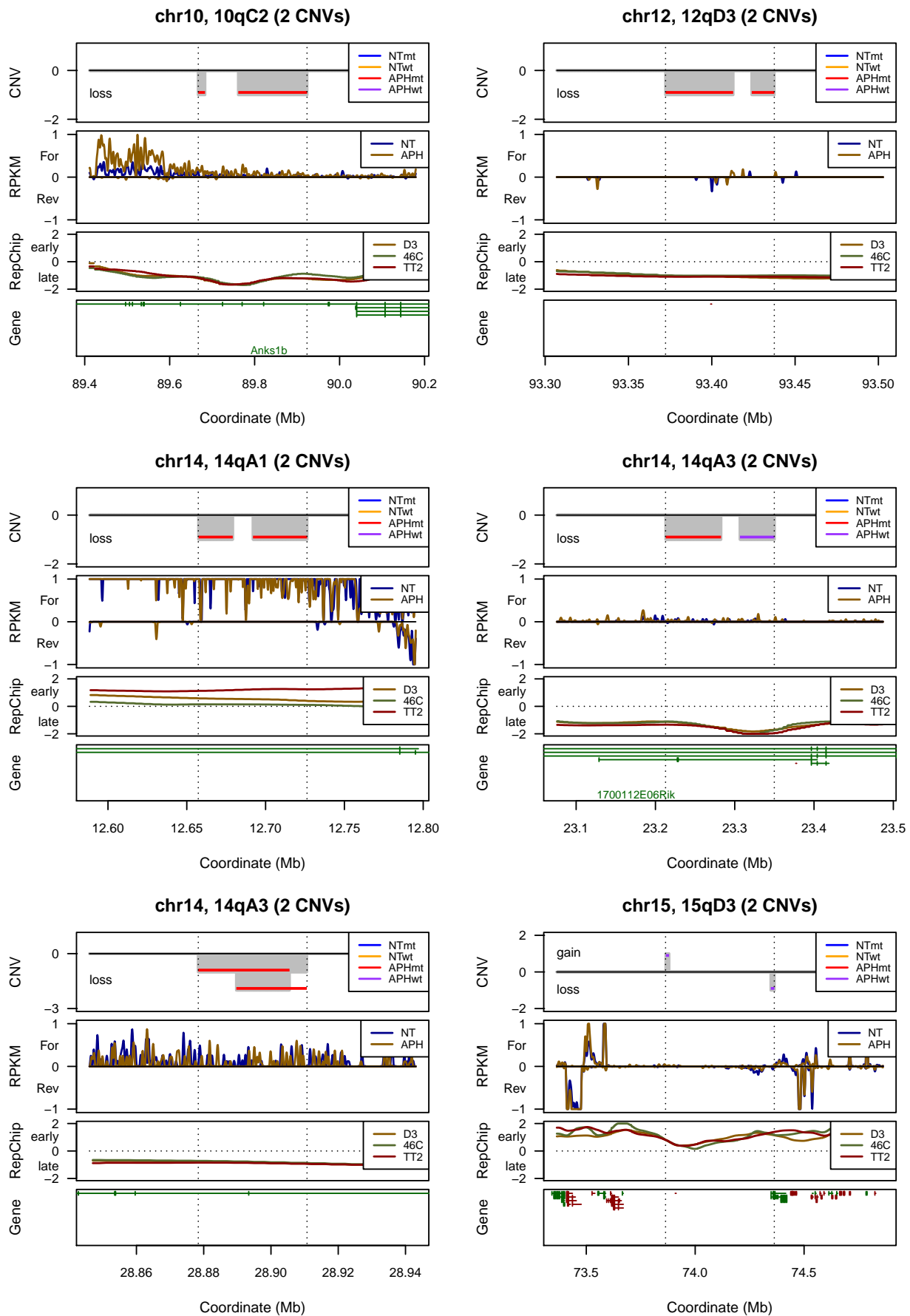

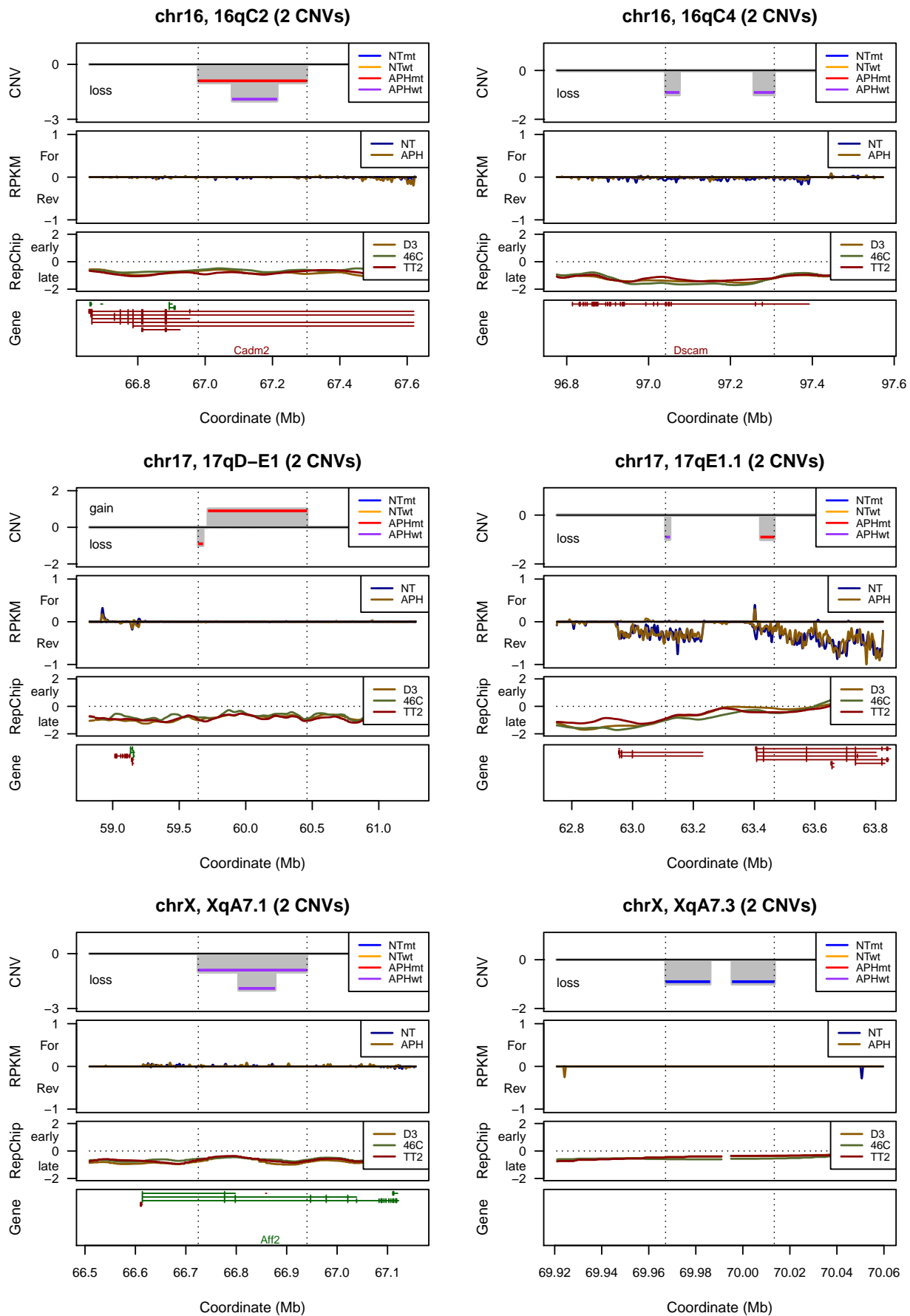

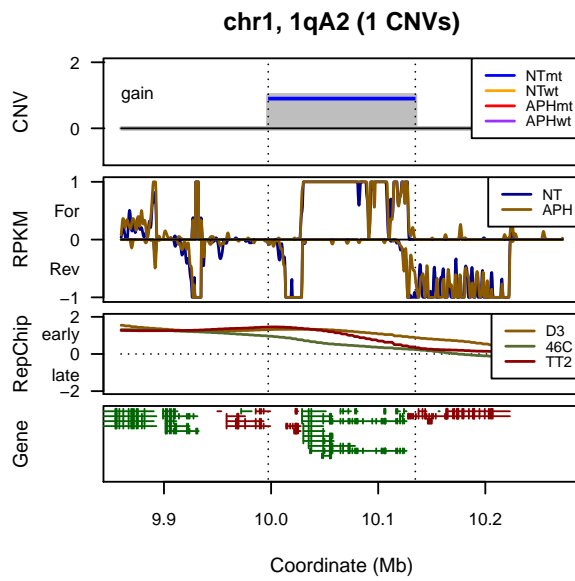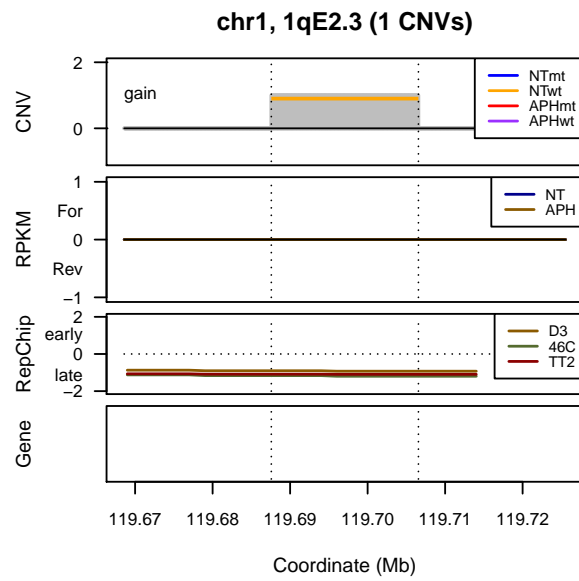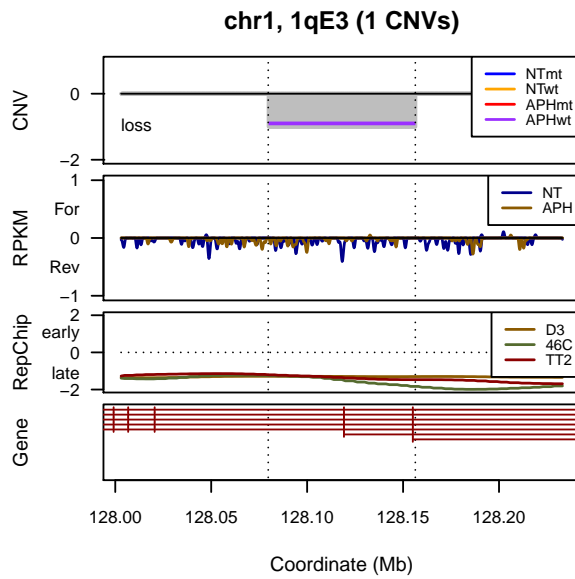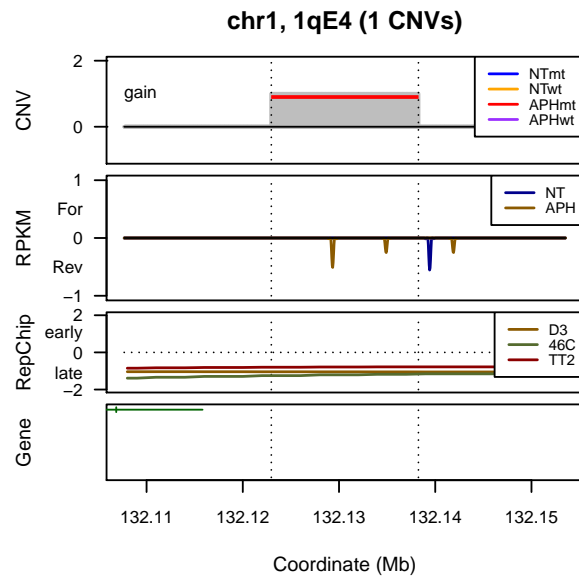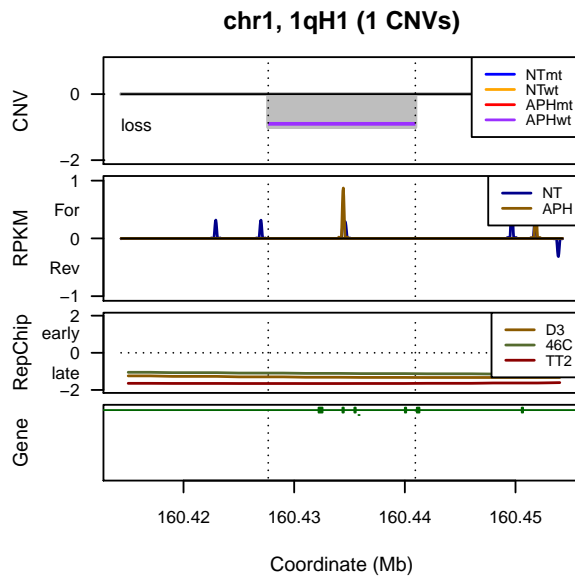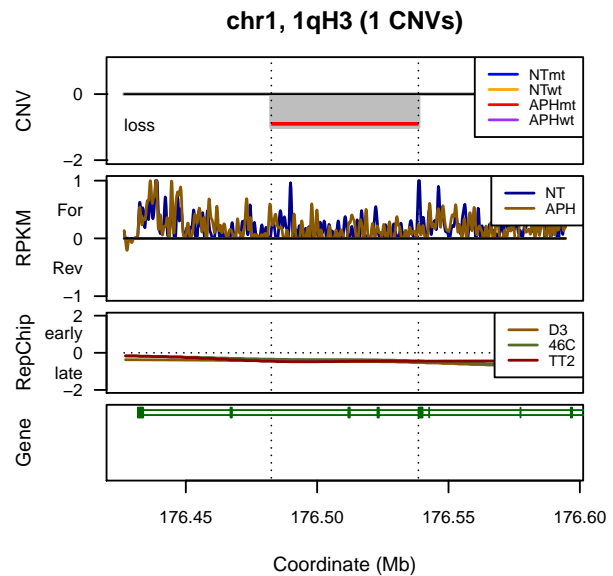

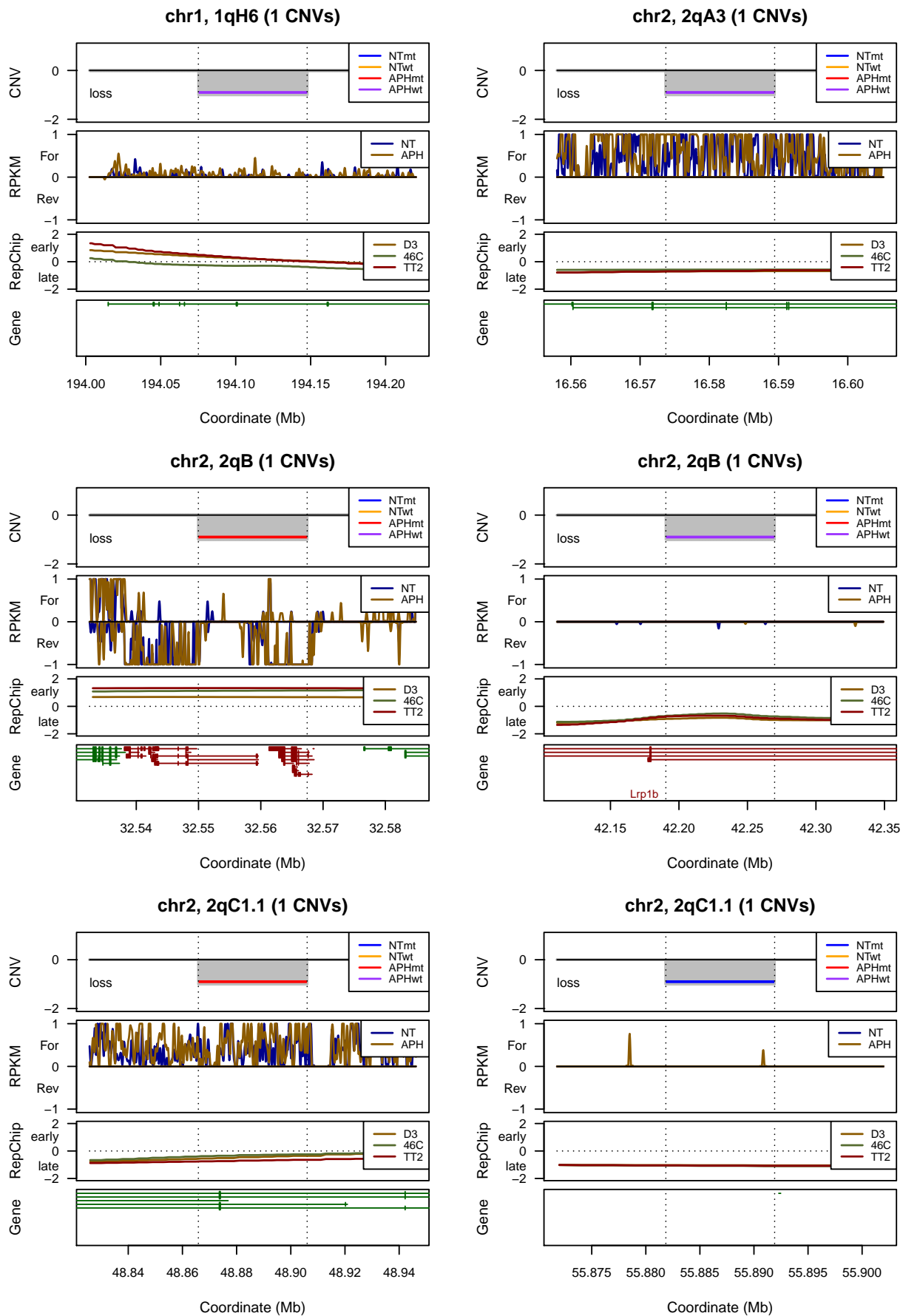

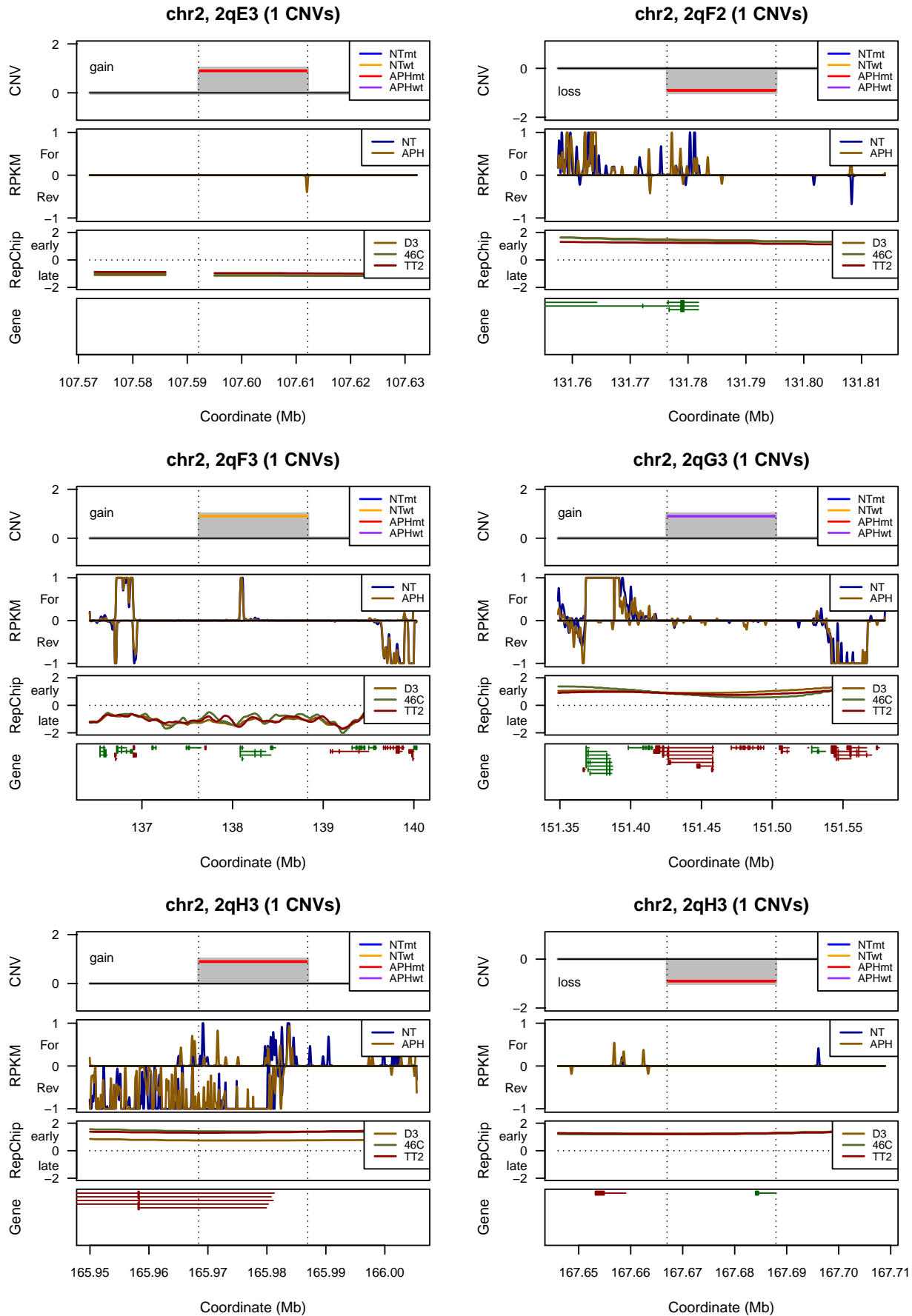

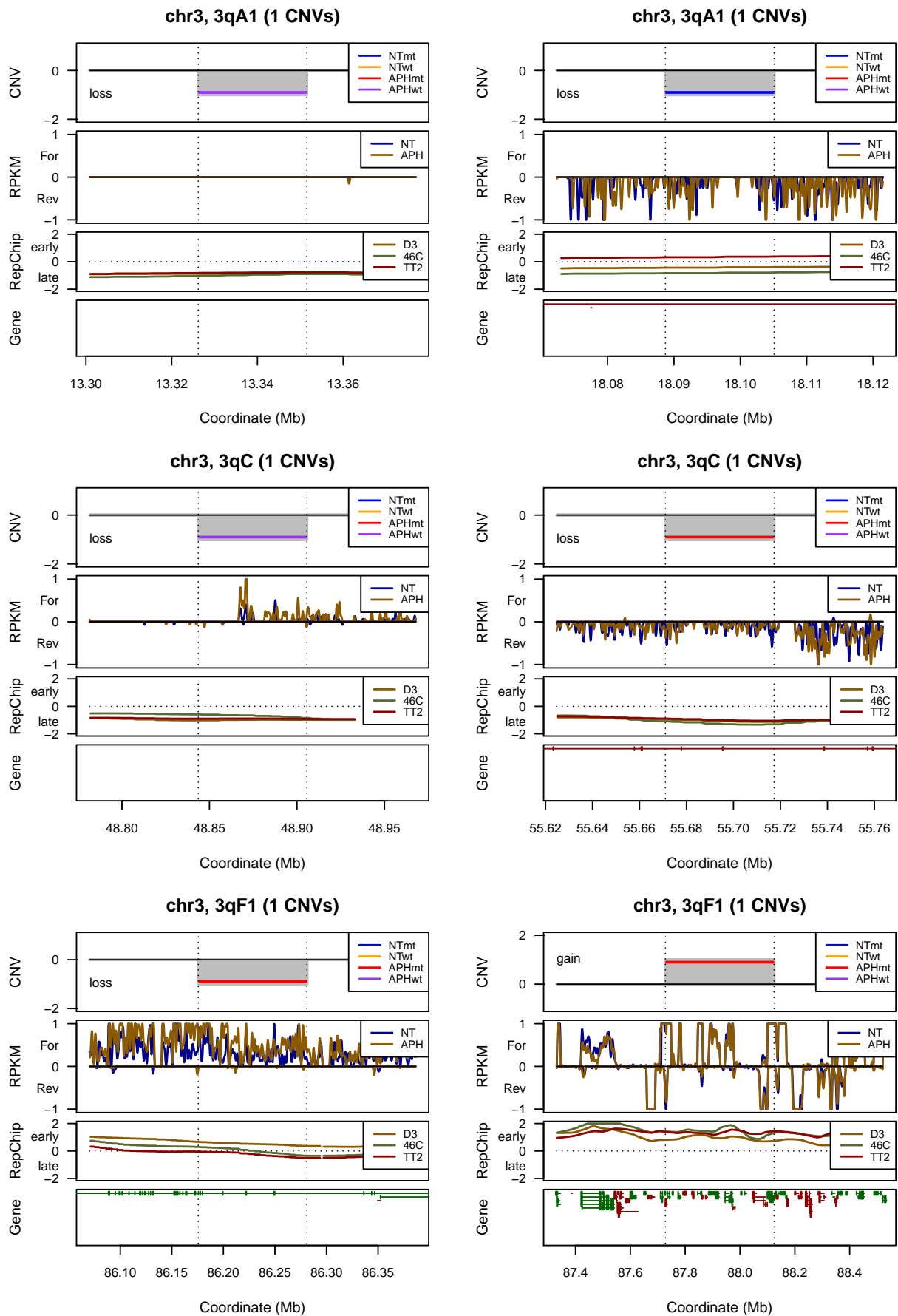

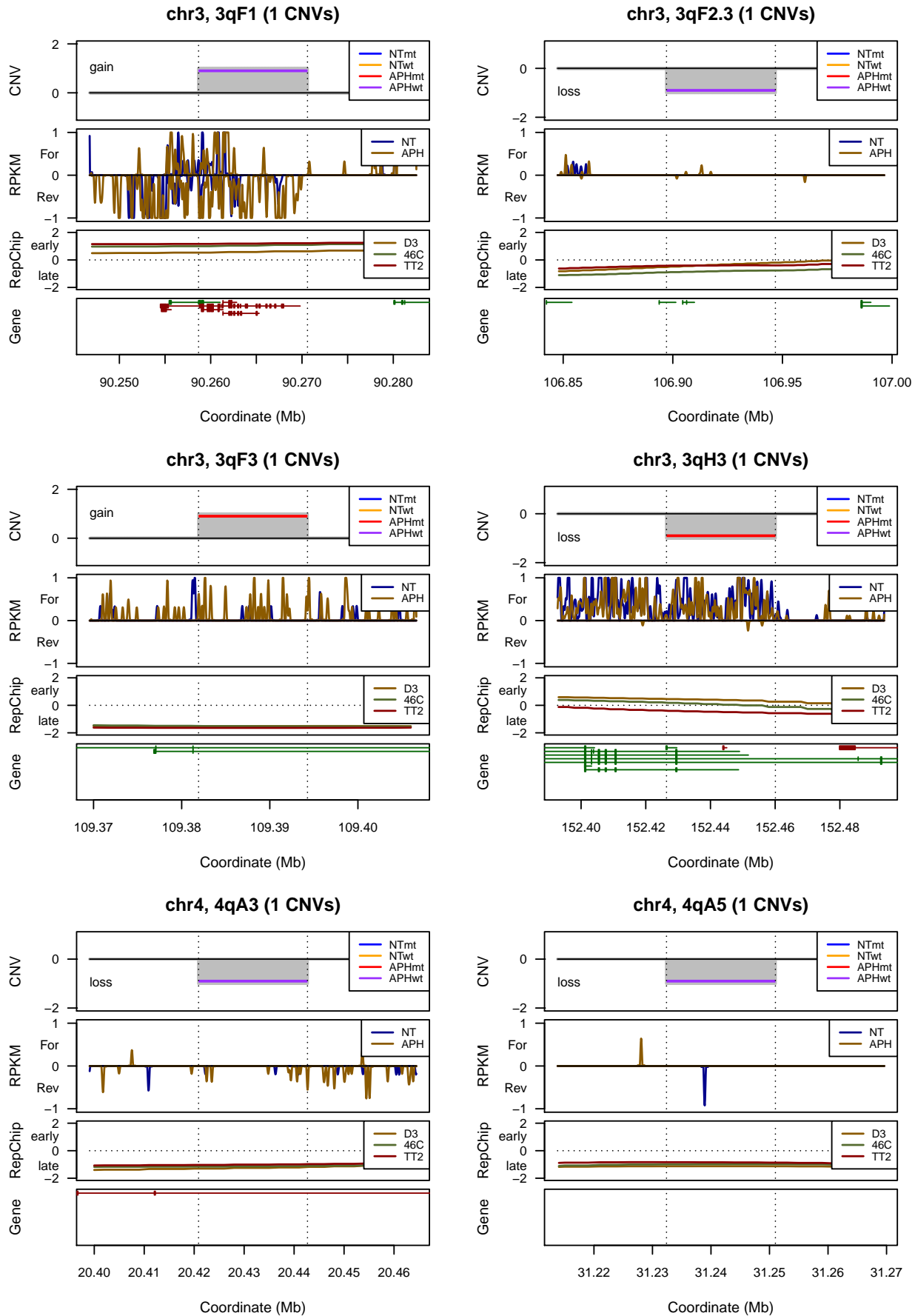

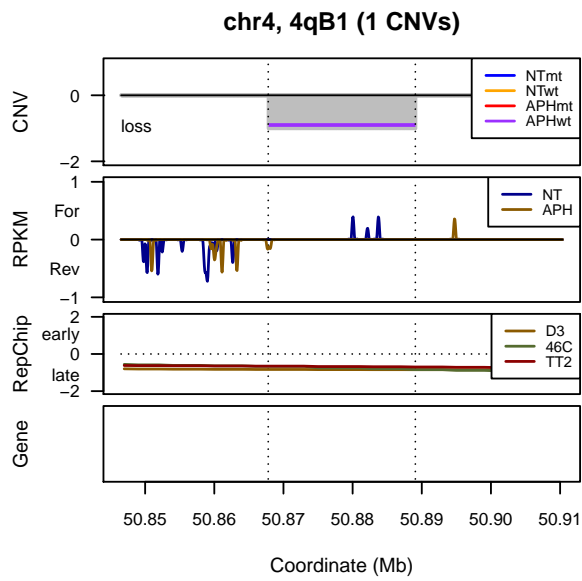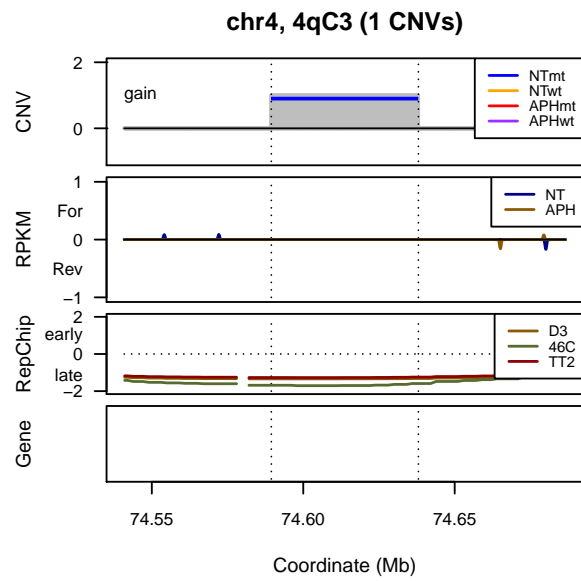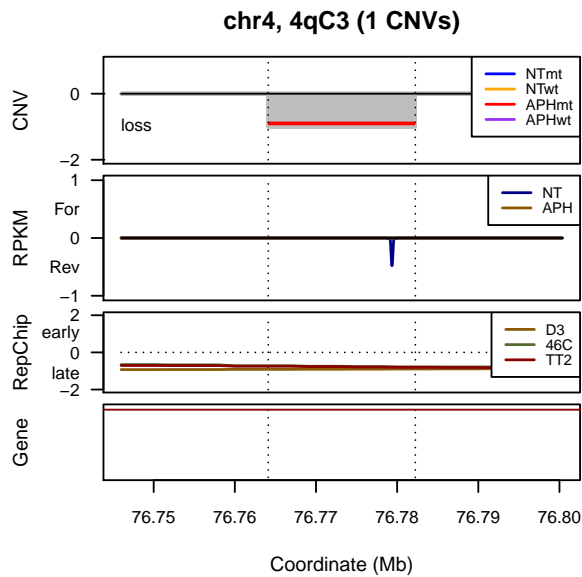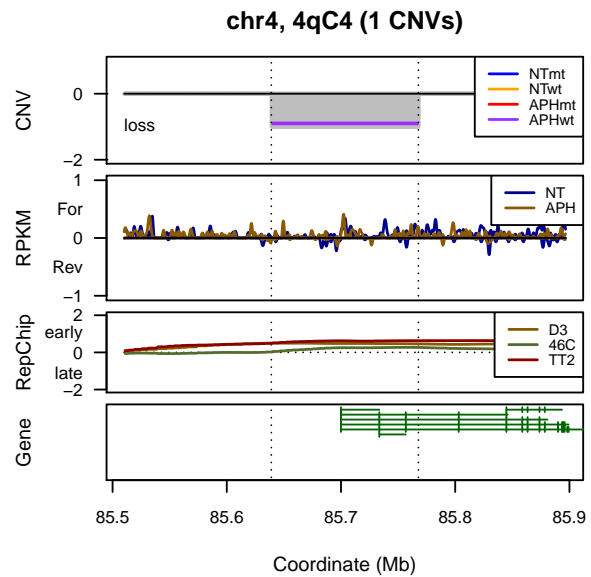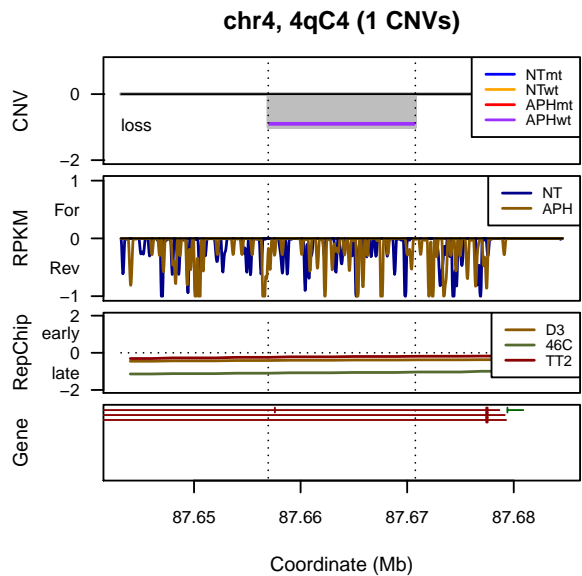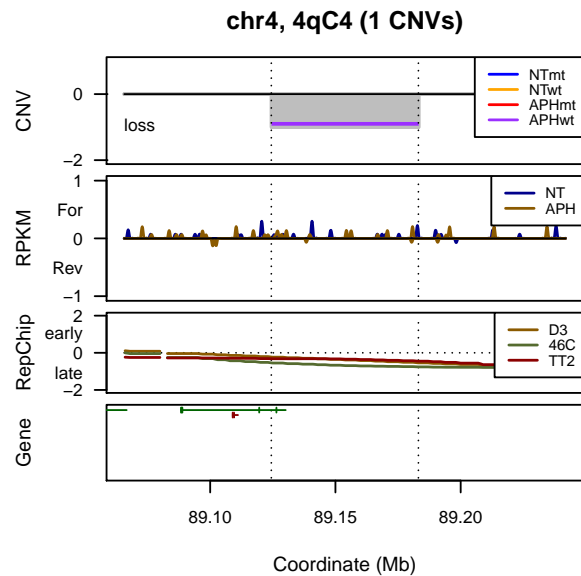

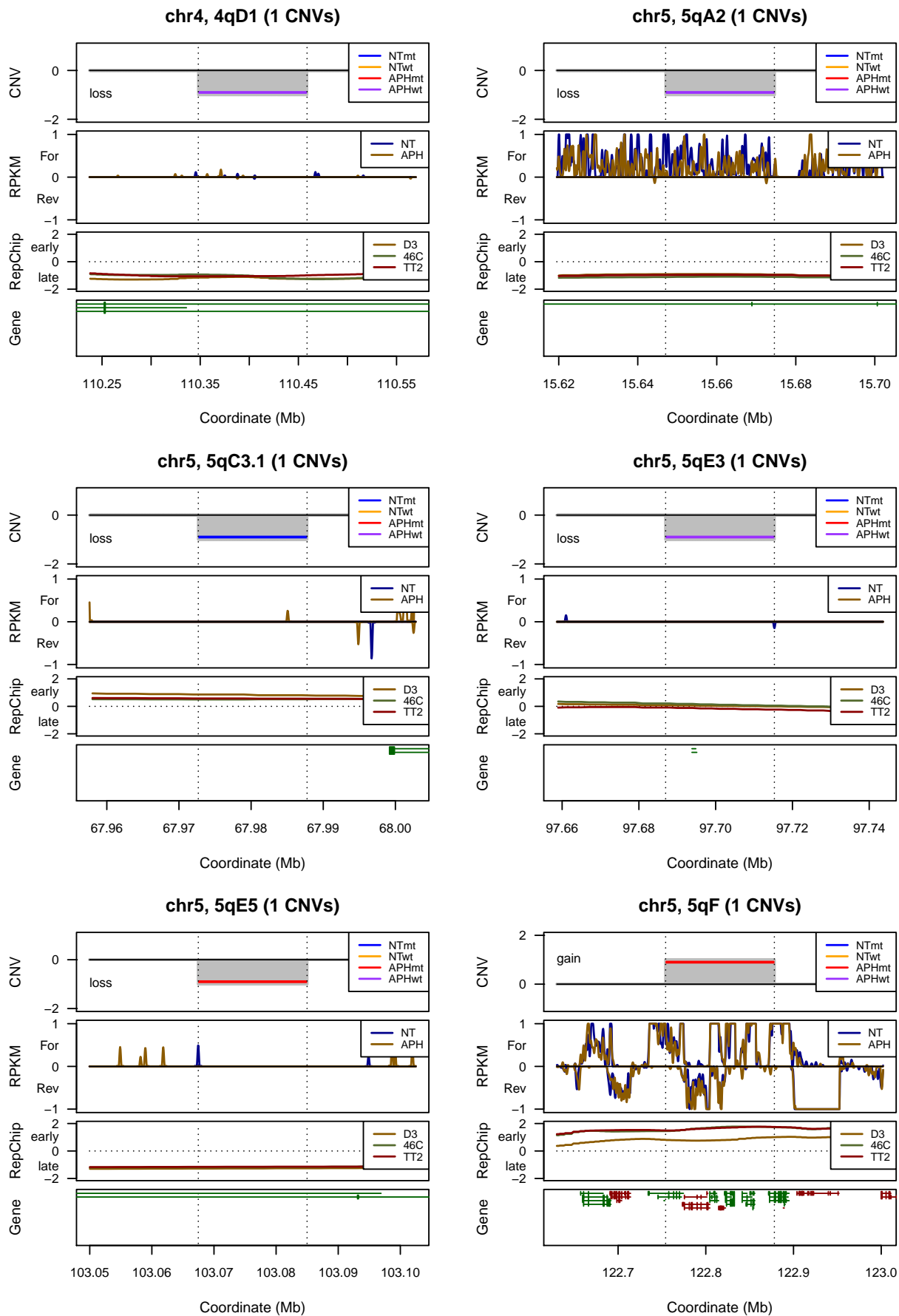

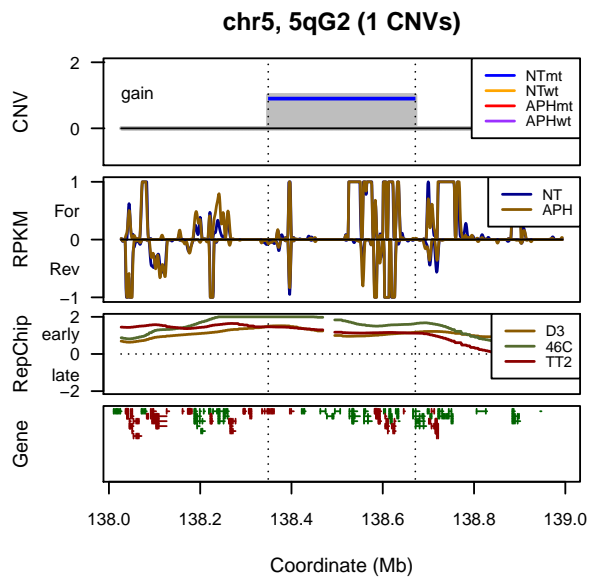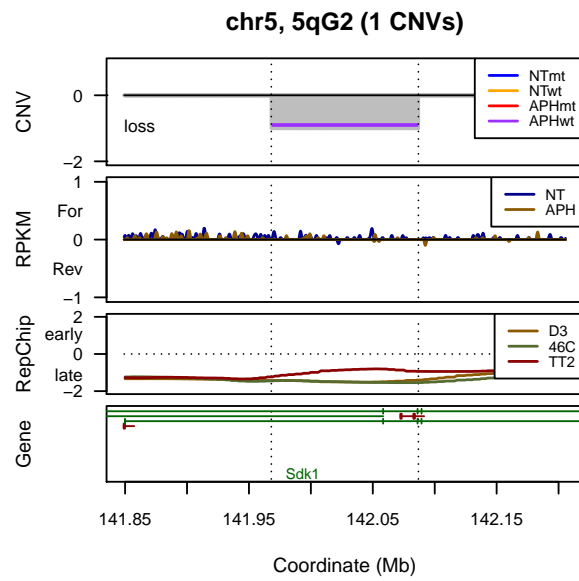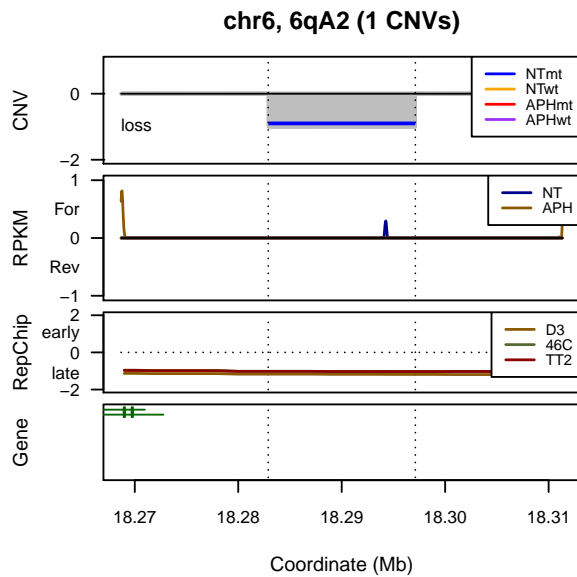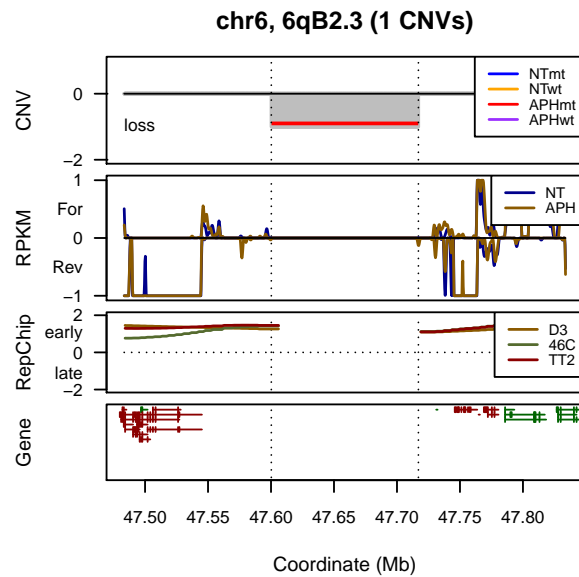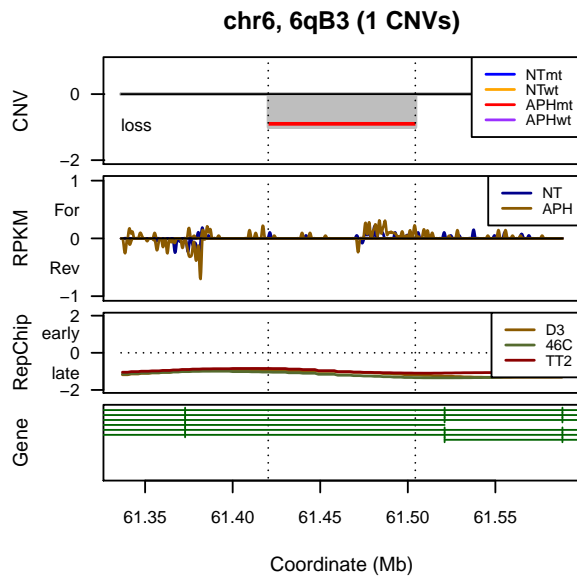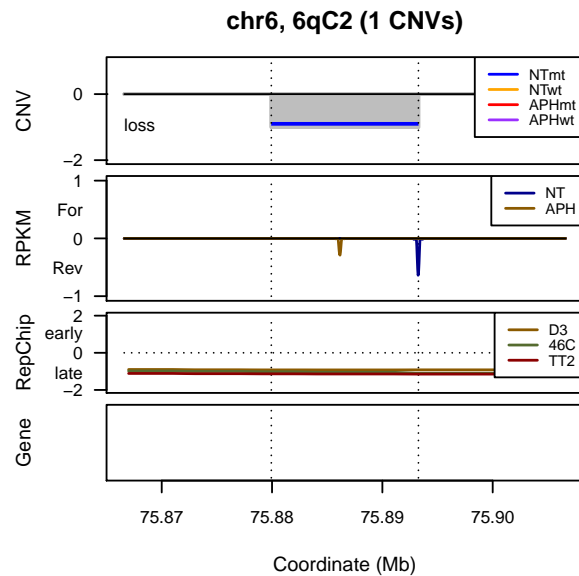

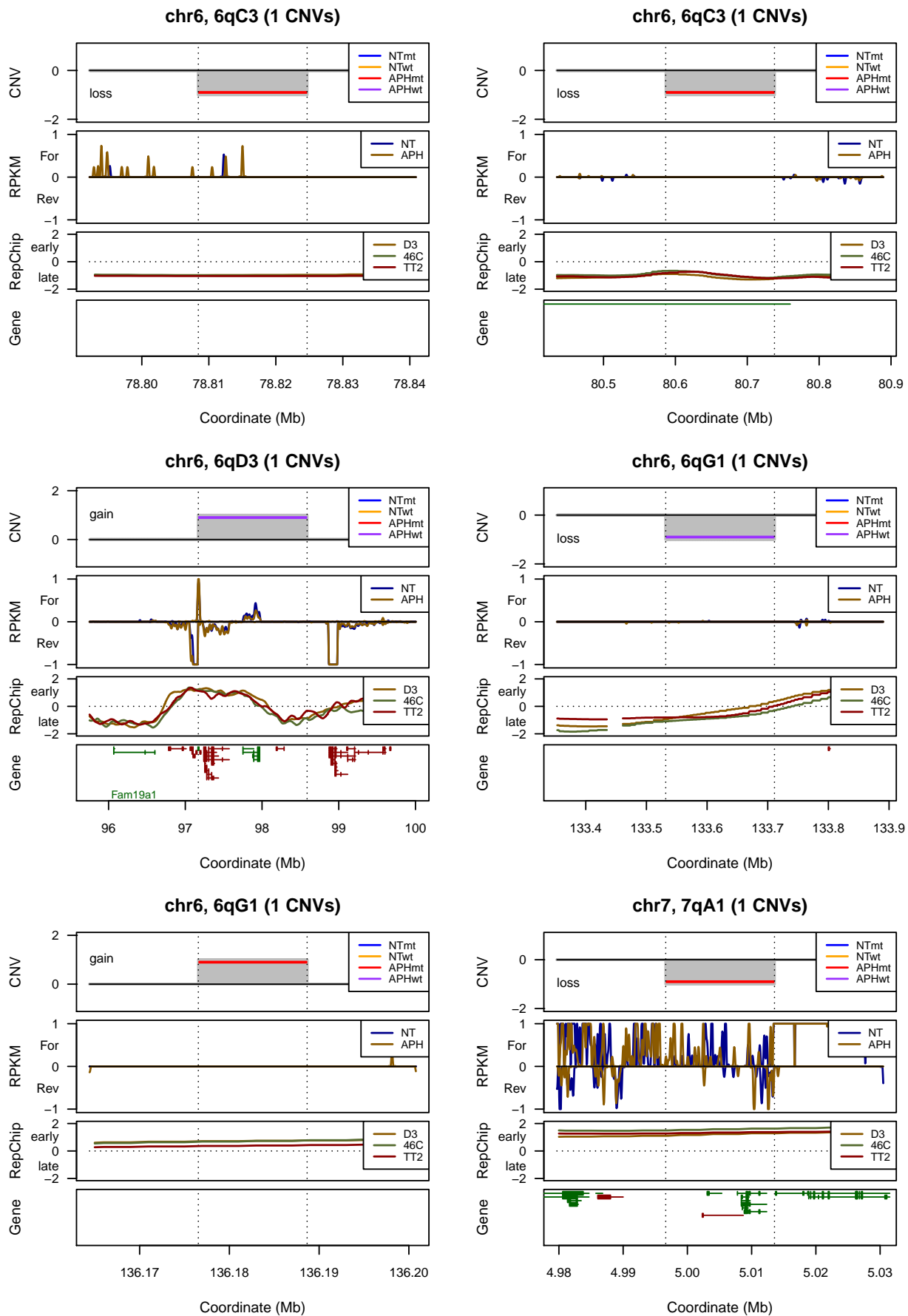

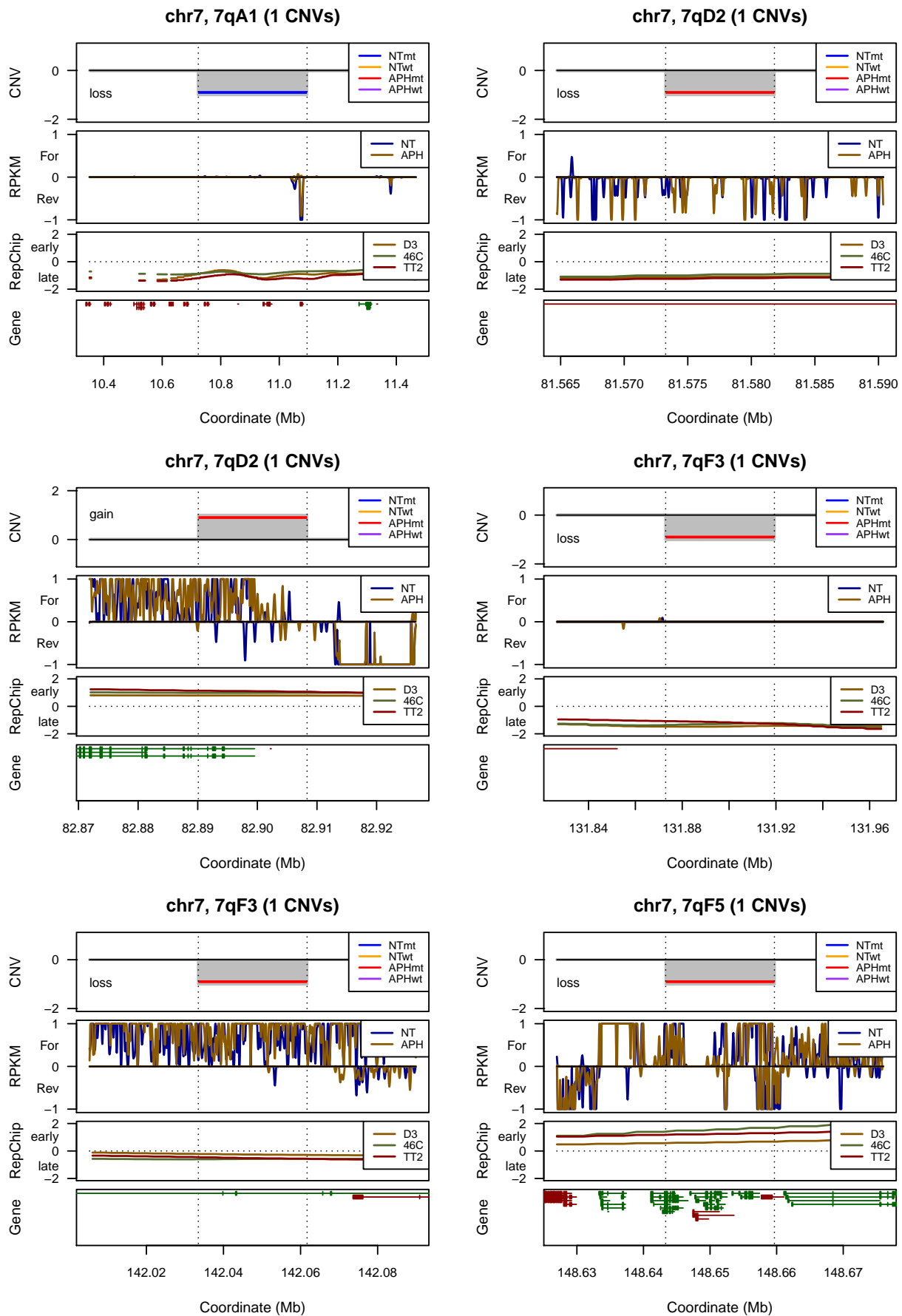

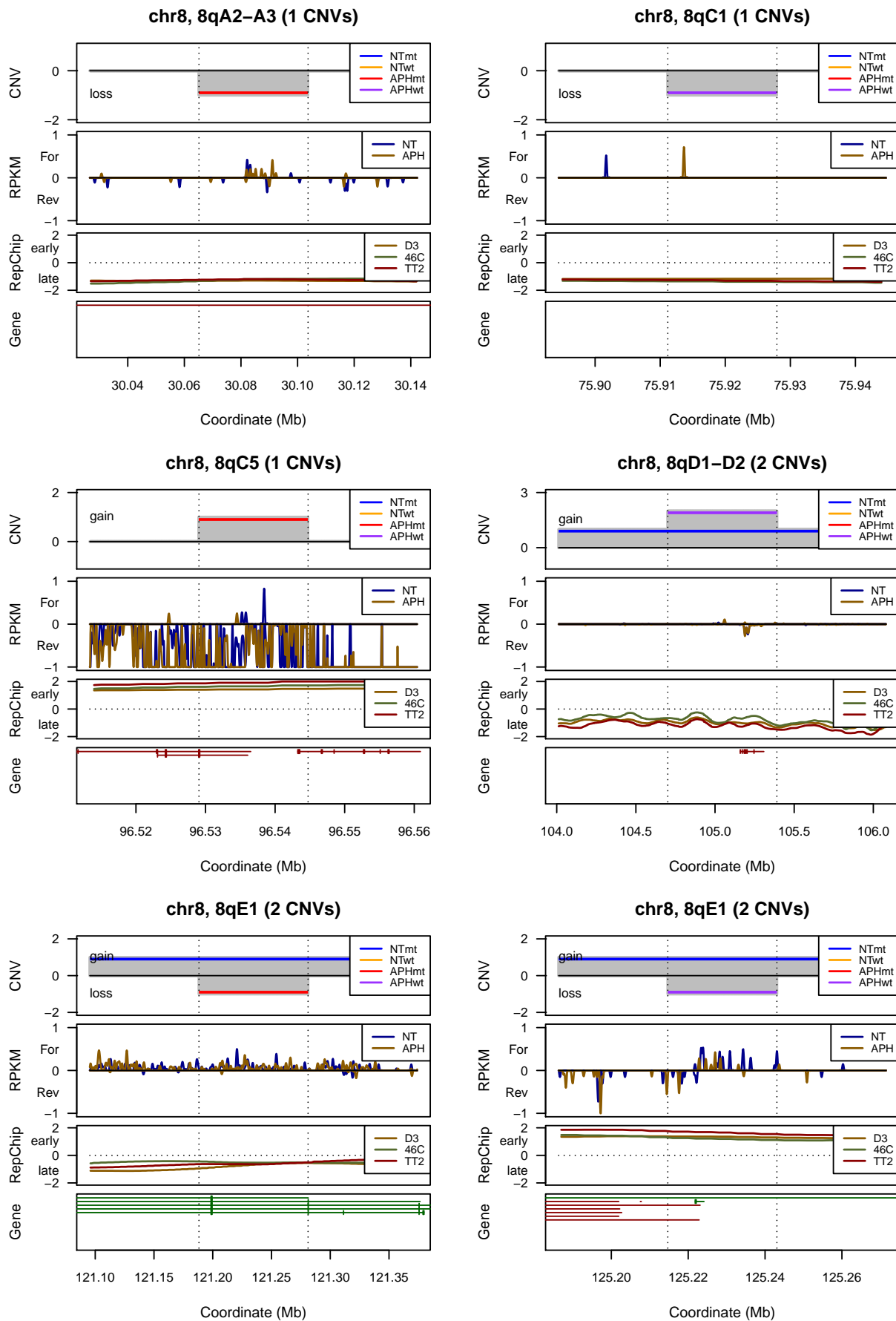

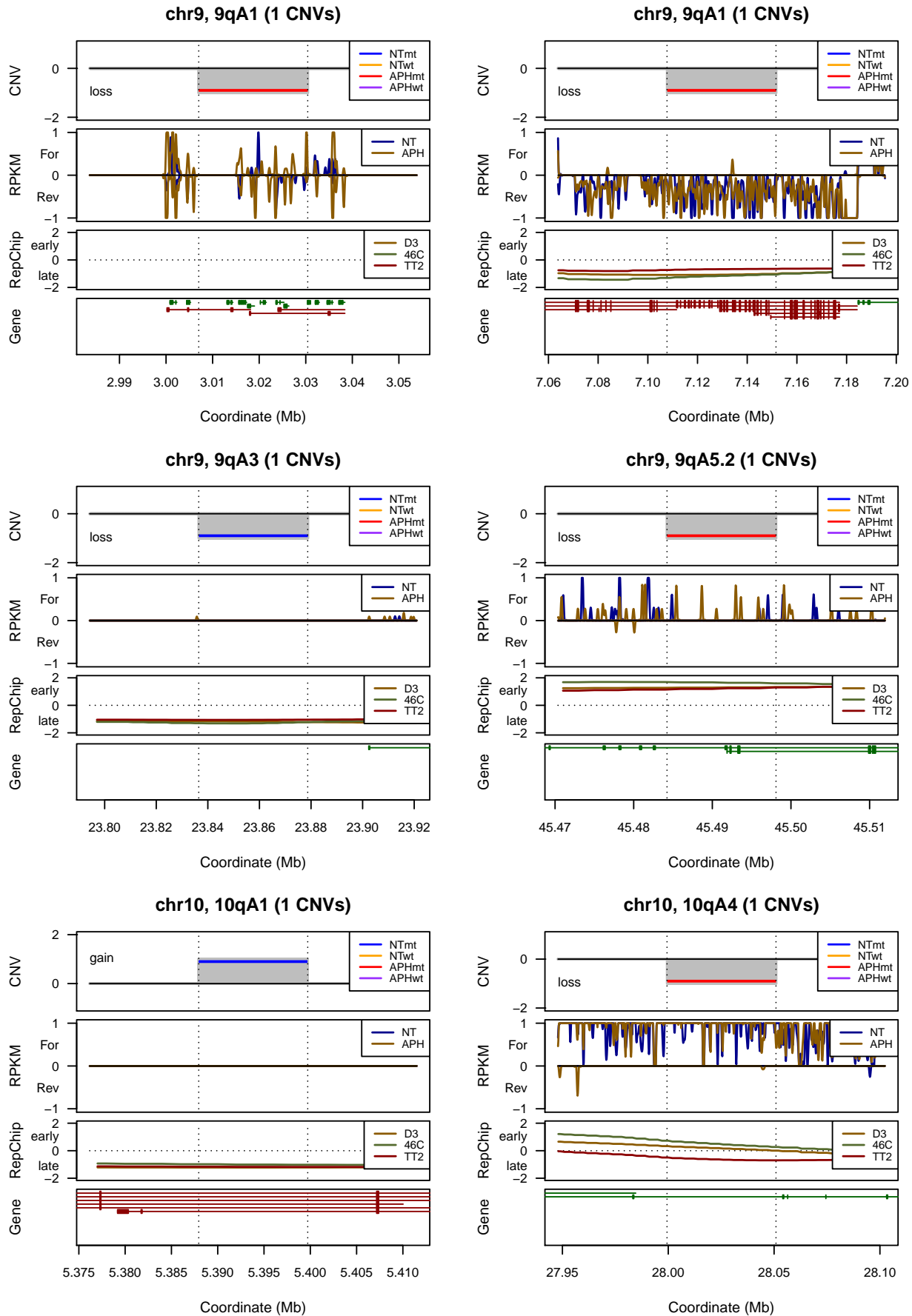

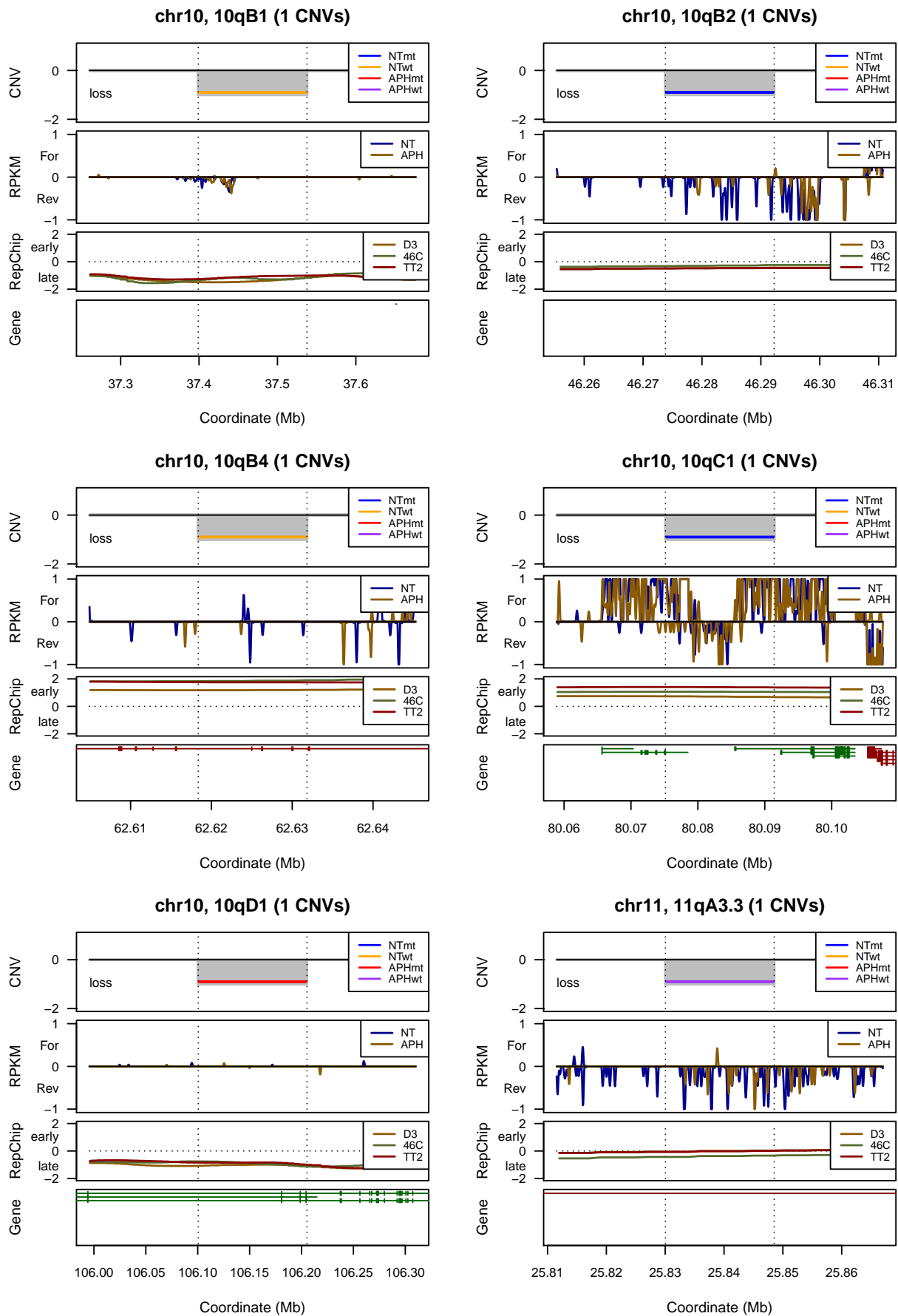

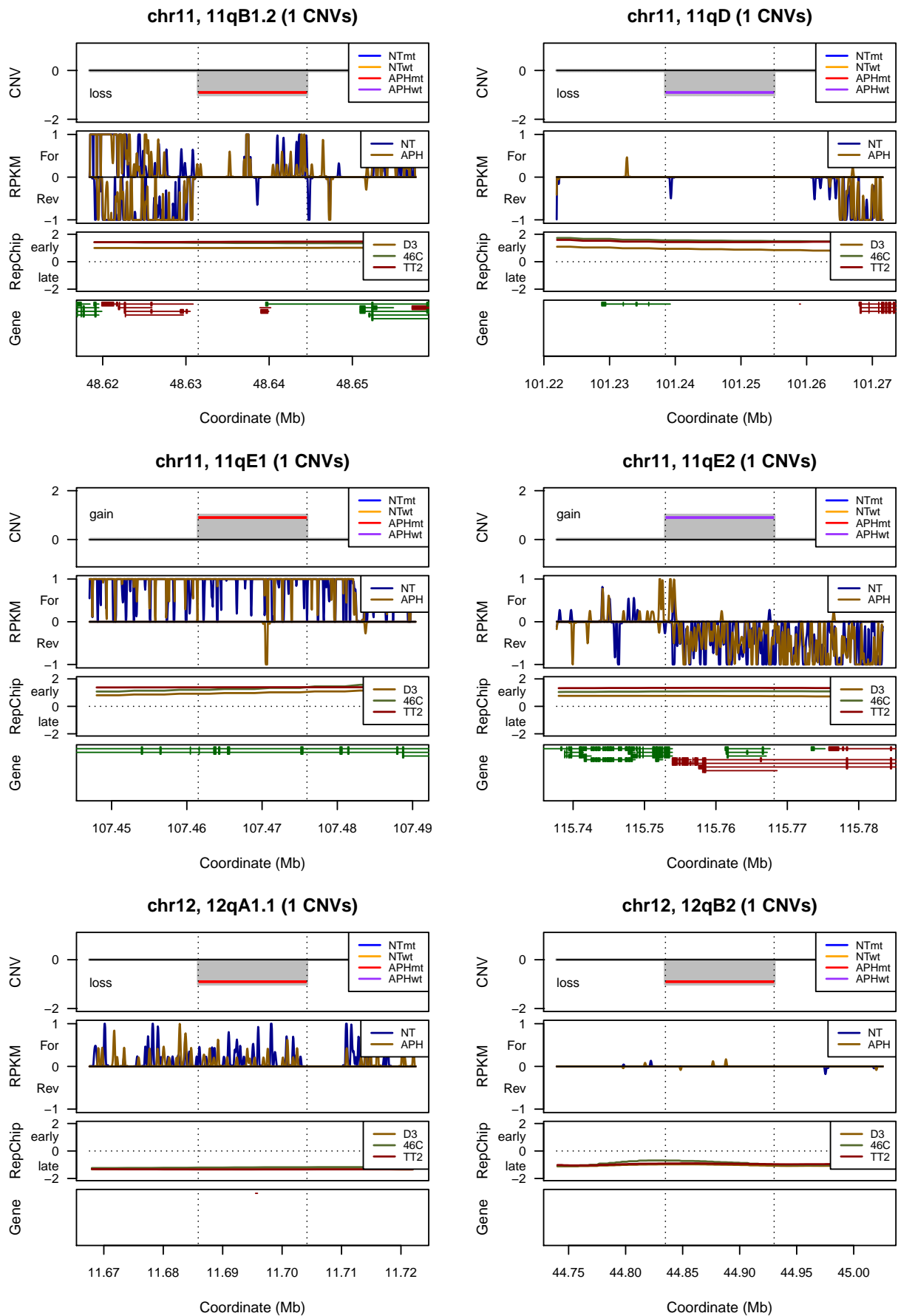

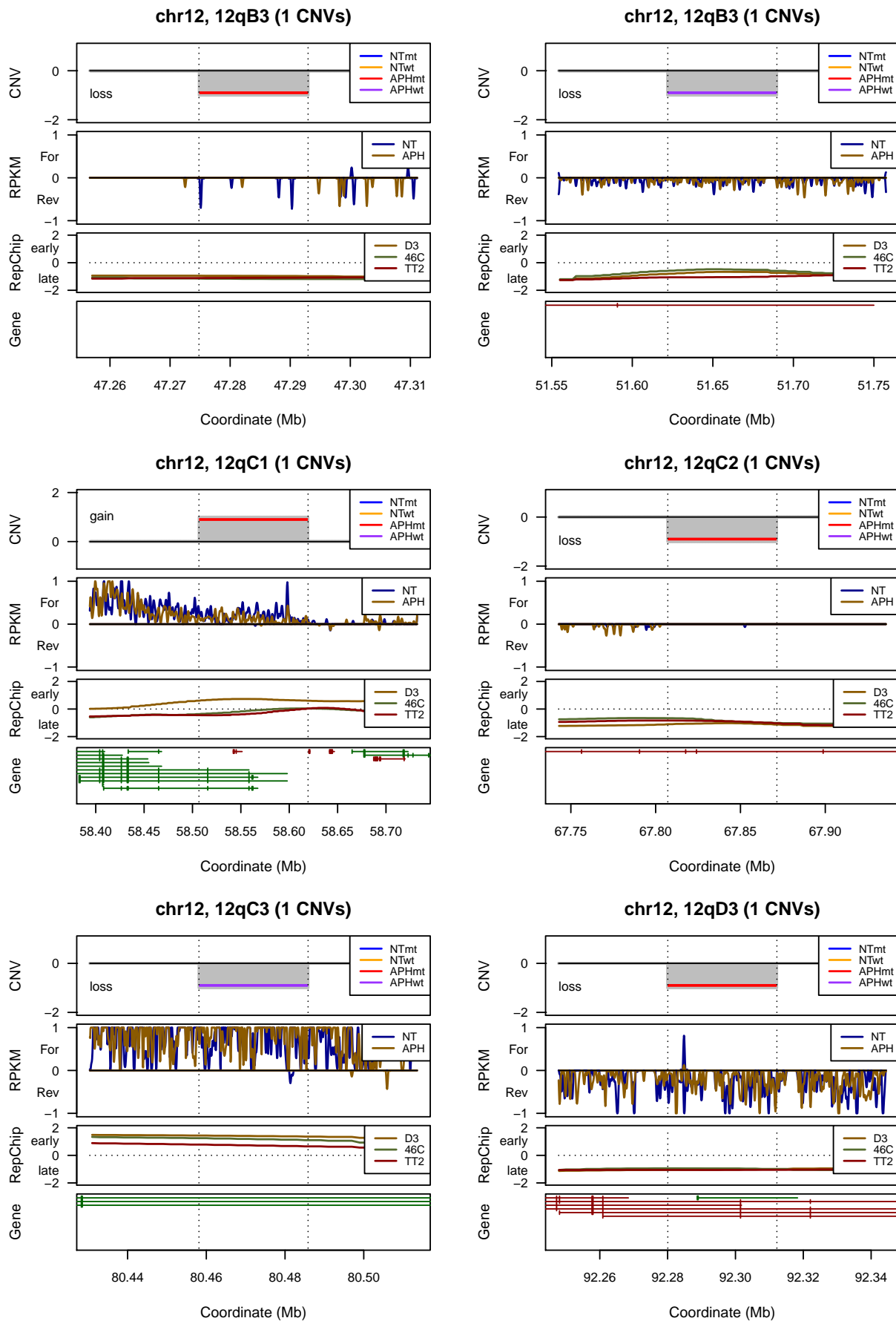

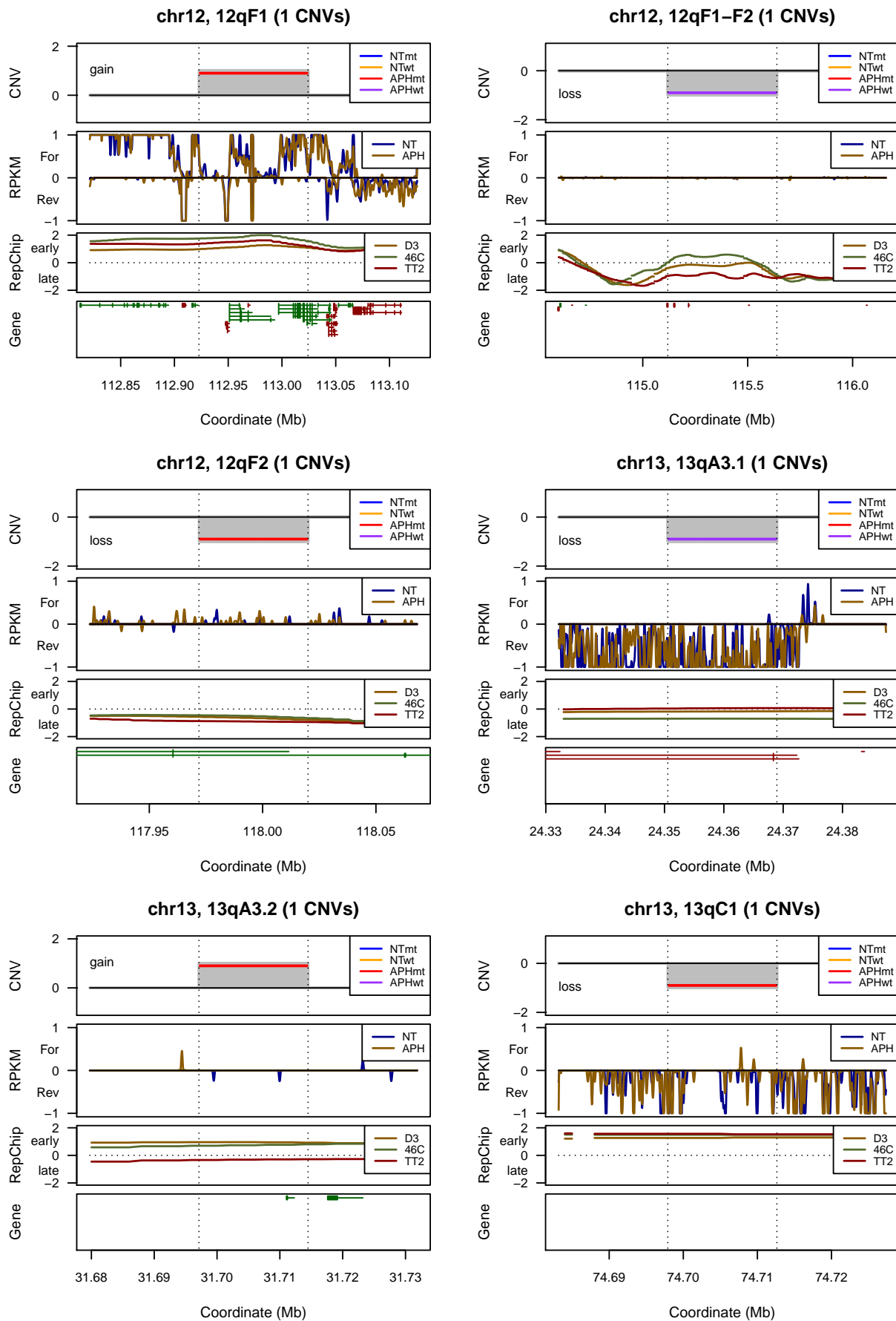

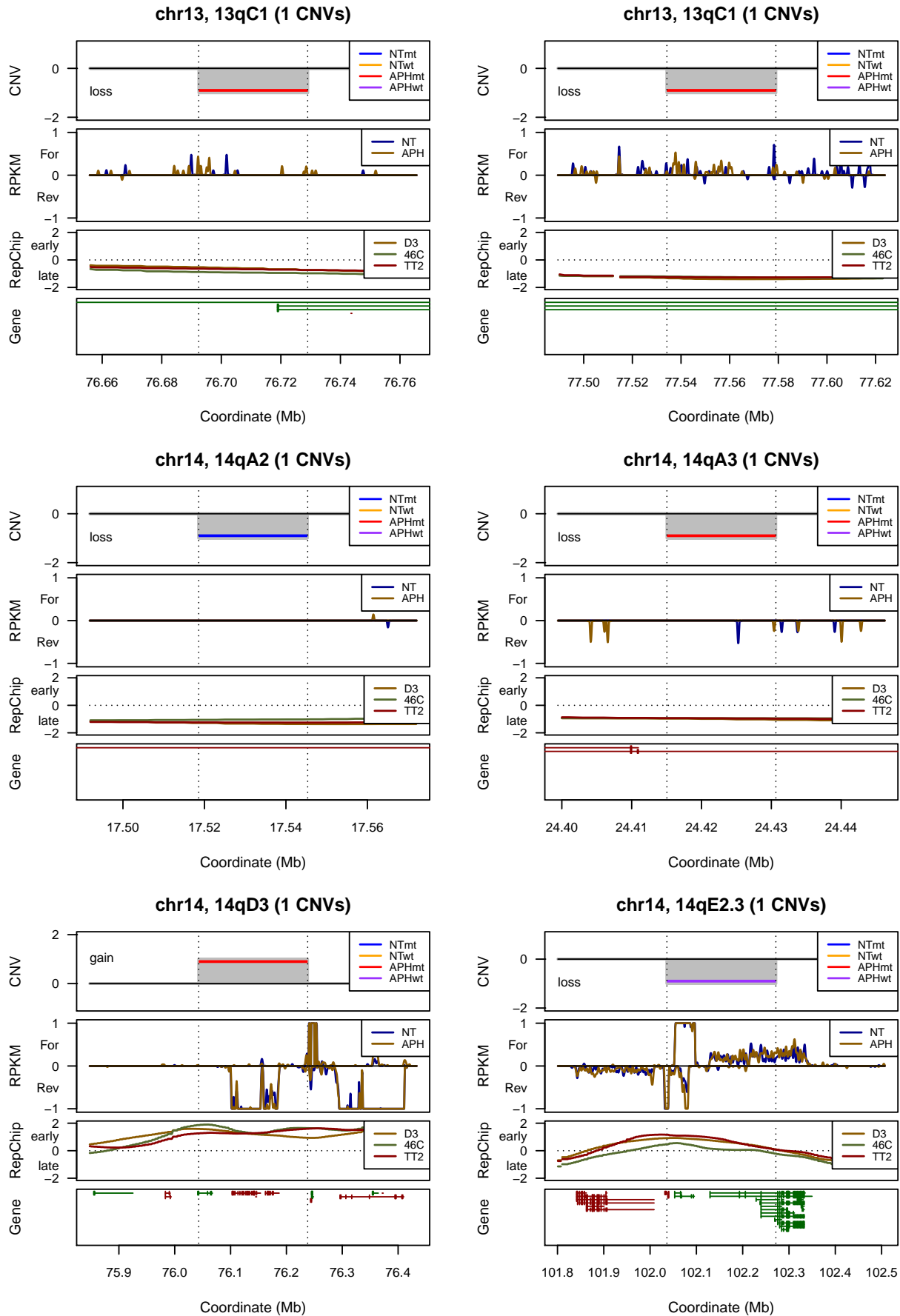

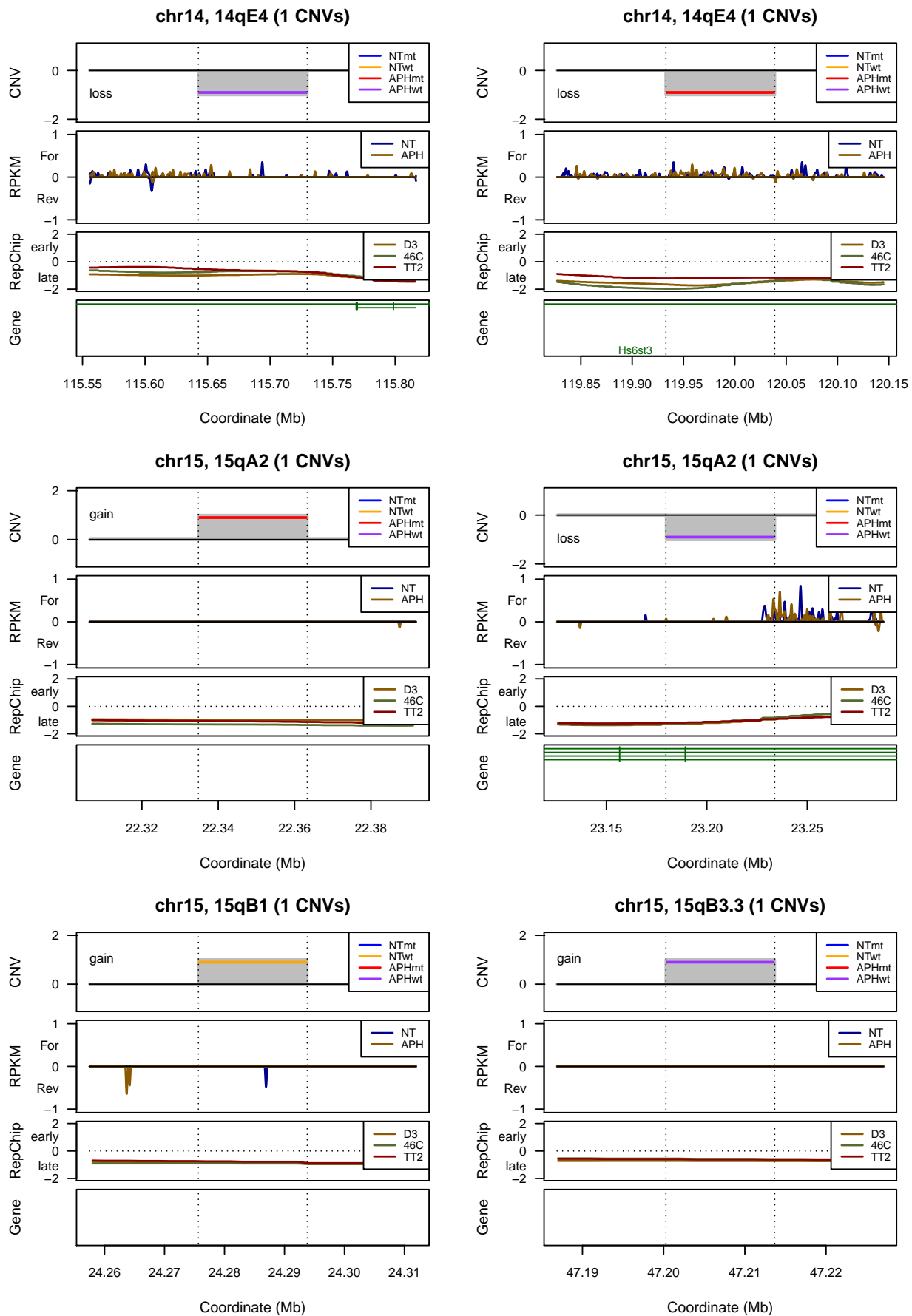

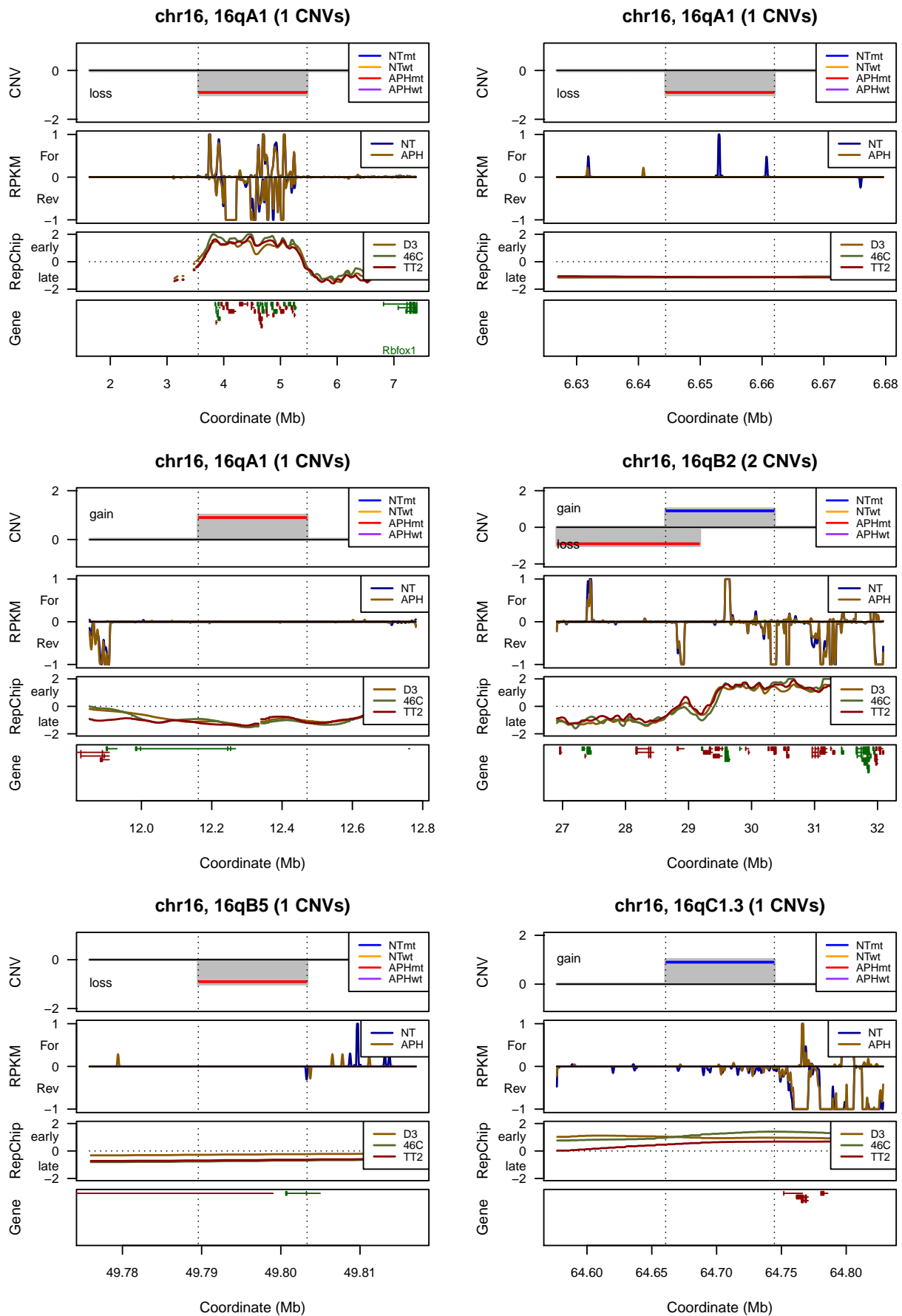

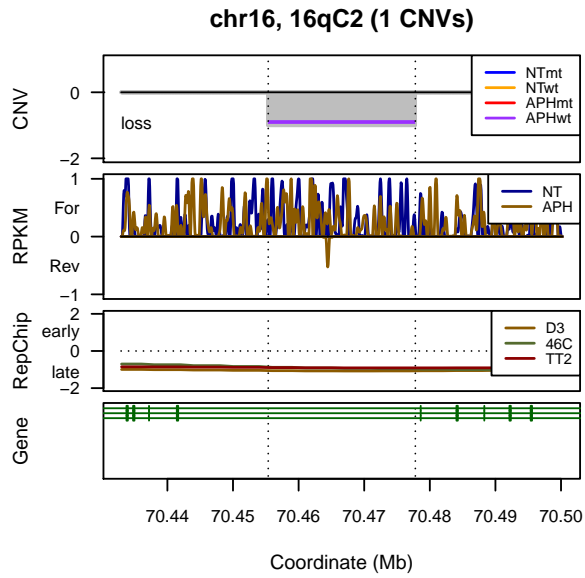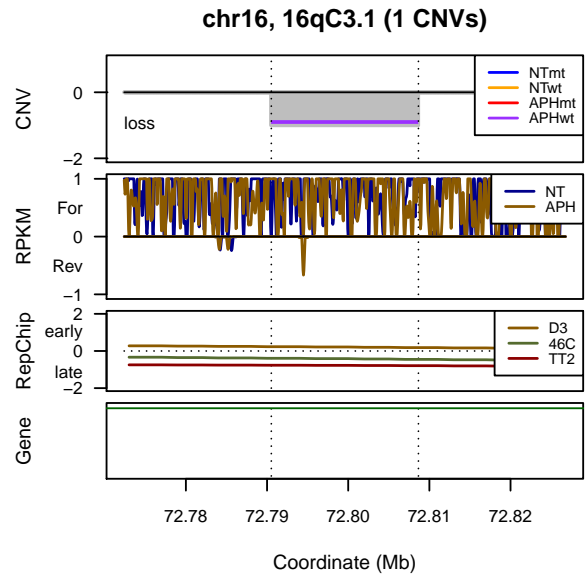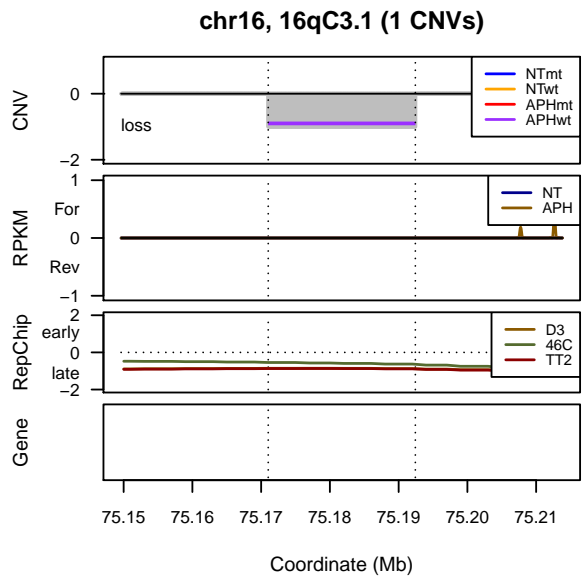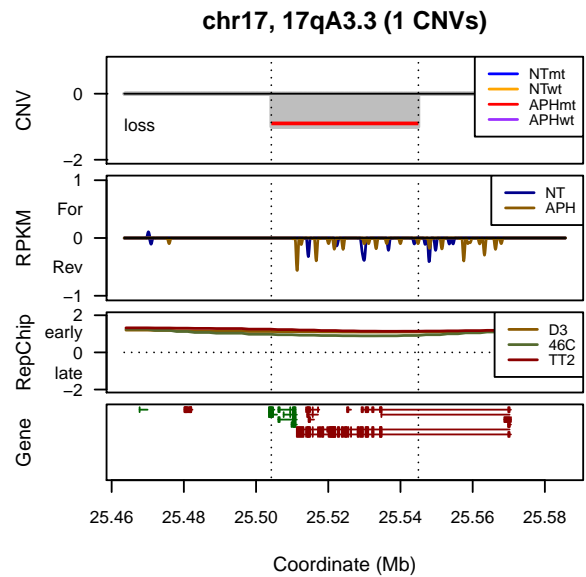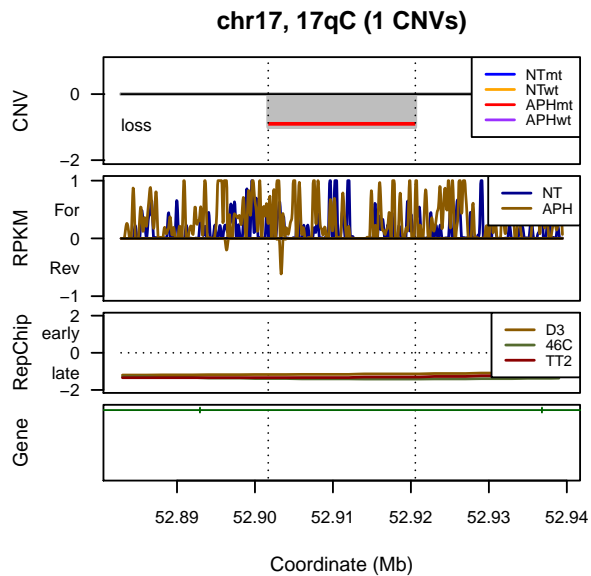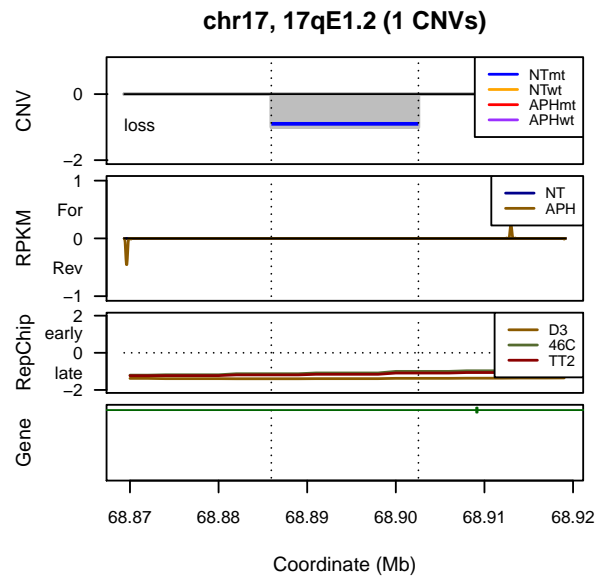

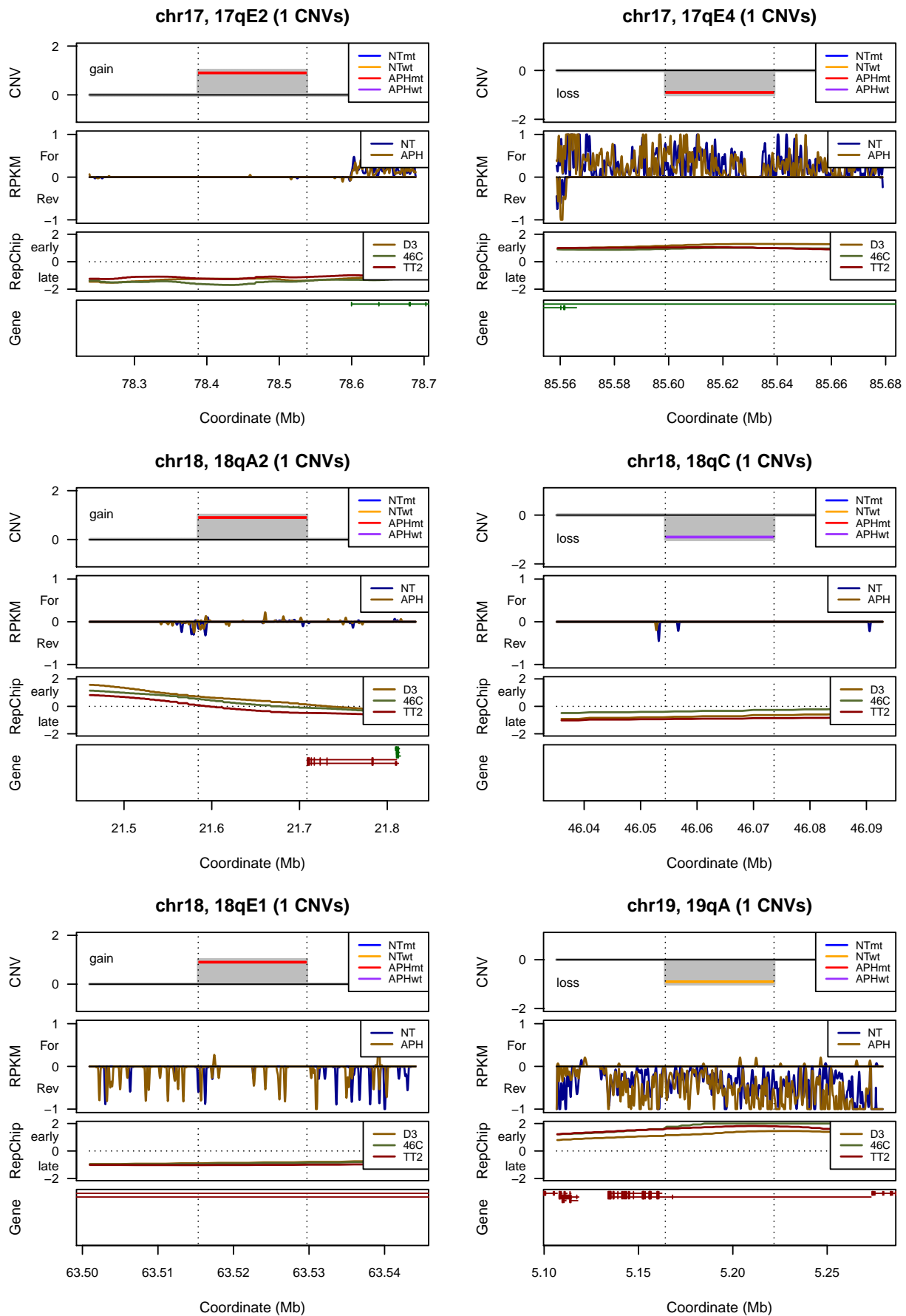

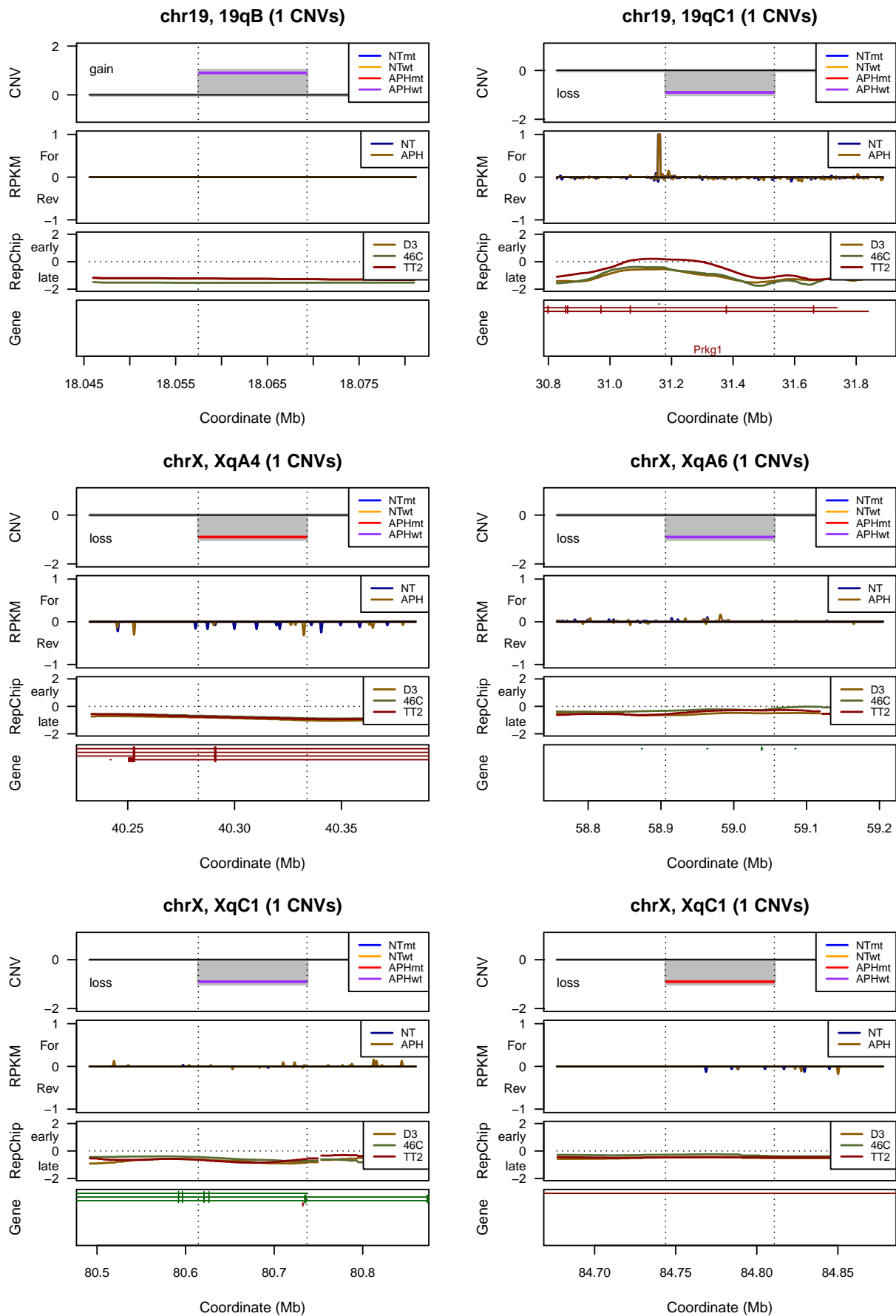

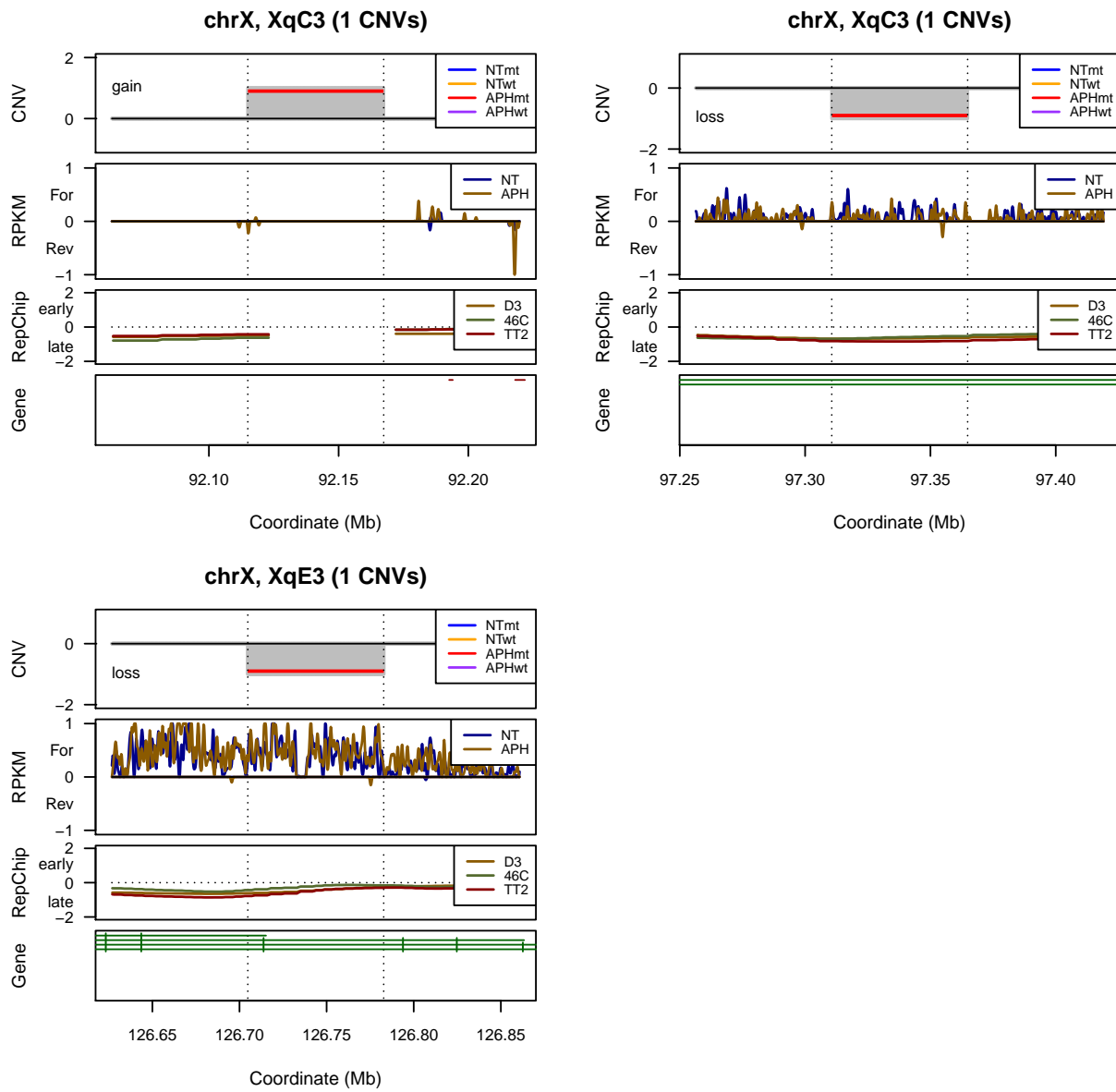

Supplement: Supplemental Material [file supp_gr.177121.114_Figure_S1H.pdf]

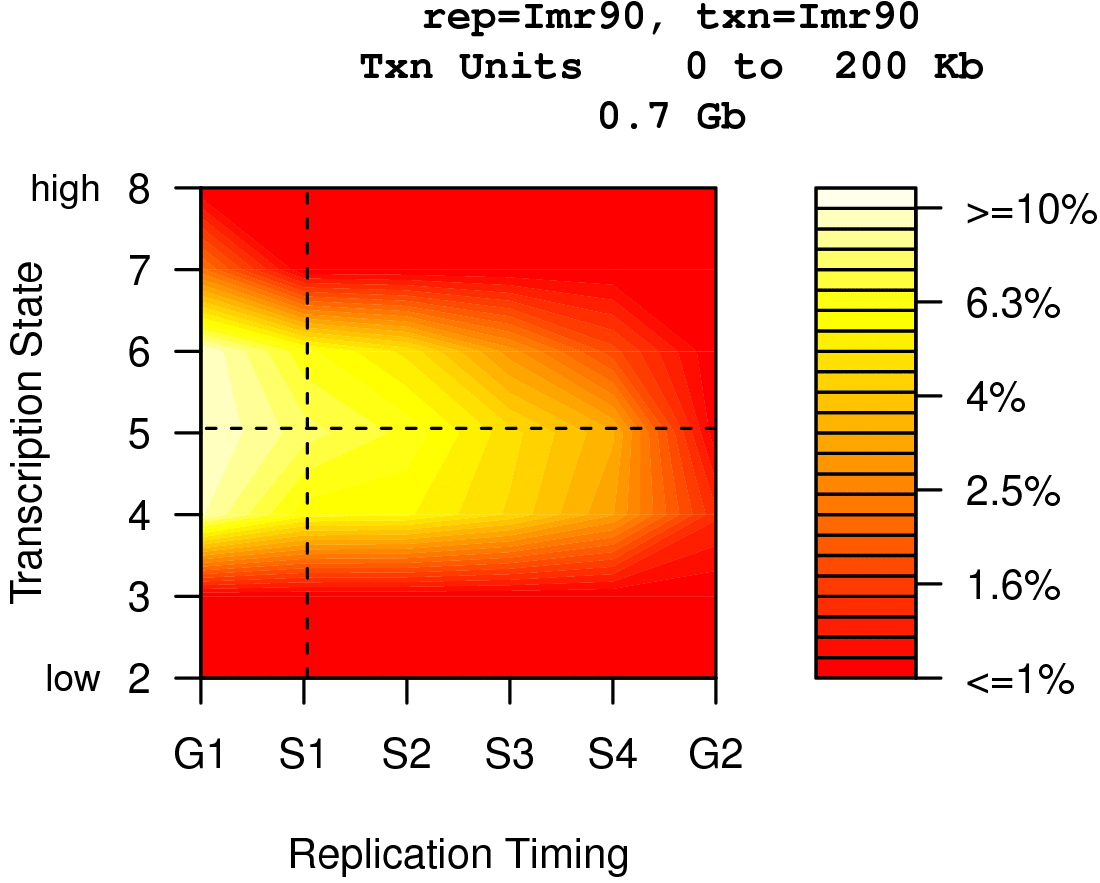

Supplement: Supplemental Material [file supp_gr.177121.114_Figure_S8H.gif]

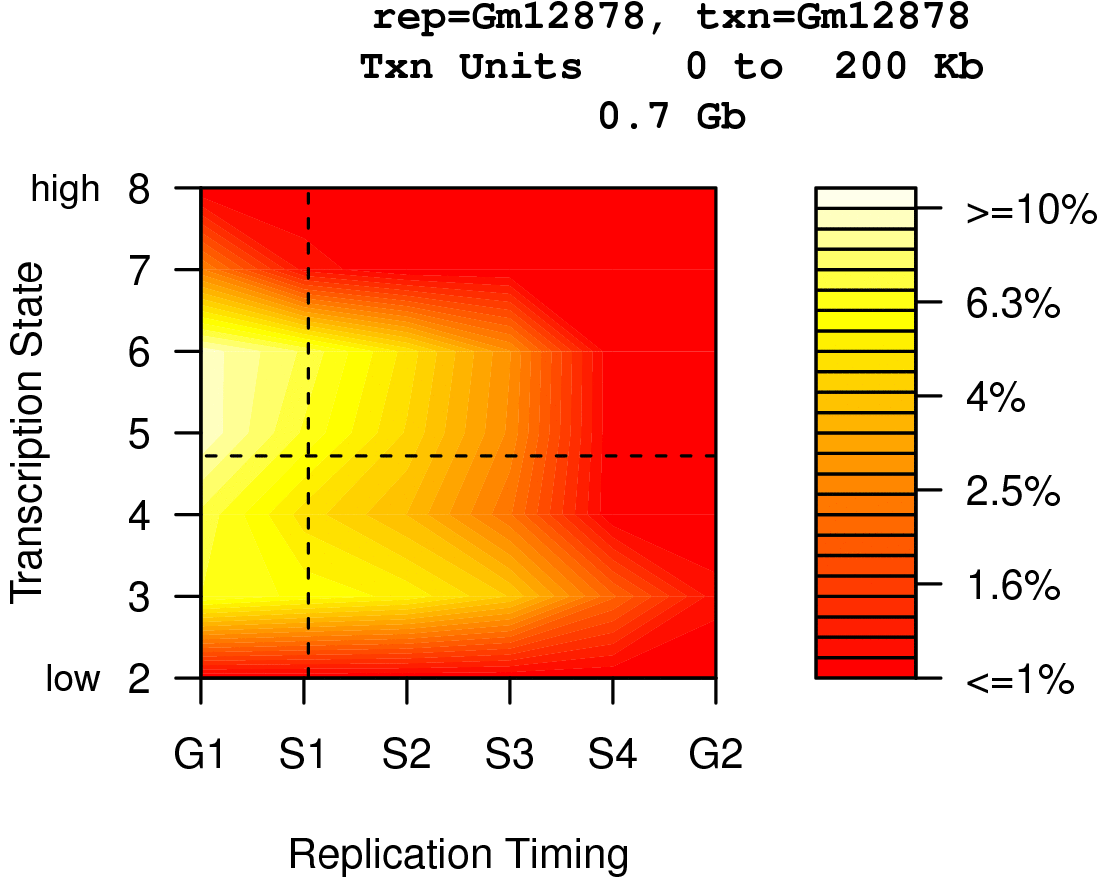

Supplement: Supplemental Material [file supp_gr.177121.114_Figure_S8I.gif]
