# Supplementary material for: Large transcription units unify copy number variants and common fragile sites arising under replication stress
Source: Genome Res. 2015 Feb;25(2):189–200. doi: 10.1101/gr.177121.114 (PMC4315293; doi:10.1101/gr.177121.114)
Supplement: Supplemental Material [file supp_gr.177121.114_Supplemental_Material.pdf]

Supplemental Information - Table of Contents

Supplemental Methods **2**
SNP PCR detection of *de novo* CNVs 2
Bioinformatics pipeline 2
CNV cluster region identification 2
Transcription unit identification 3
Comparison to reference genome features 4
Enrichment analysis 4
Correlation analysis 5
Repli-seq replication timing 5
Repli-chip replication timing 6
Transcription-replication correlation 6

Supplemental Figures **7**
Figure S1. Additional CNV region profiles 7
Figure S2. Common fragile sites 8
Figure S3. Additional base and gene content plots 9
Figure S4. Bru-seq sample and state comparisons 10
Figure S5. mES cell transcription plots 11
Figure S6. Additional transcription plots 12
Figure S7. mES cell replication timing plots 13
Figure S8. Additional replication timing plots 14
Figure S9. Late replication of large genes does not depend on transcription 15
Figure S10. mES cell transcription unit alignment plots 16
Figure S11. Additional transcription unit alignment plots 17

Supplemental Tables **18**
Table S1. CNV counts and size distributions 18
Table S2. CNV cluster thresholds 19
Table S3. CNVs, genes, transcription units, and replication regions 20
Table S4. Common fragile sites 20
Table S5. Simulation statistics 20
Table S6. Transcription unit overlaps 21
Table S7. Gaps in CNV cluster regions 23
Table S8. Human HF1 fibroblast CNVs 25
Table S9. CNVs in larges genes in human genomic disease and cancer 27

Supplemental References **29**

Supplemental Methods

SNP PCR detection of *de novo* CNVs

We designed the PCR primers below to flank SNPs that were informative in both 090 and HF1 fibroblasts and that conferred a restriction fragment length polymorphism. PCR was performed on HF1 genomic DNAs (Gentra PureGene) from APH-treated cell clones, followed by digestion with the indicated restriction enzymes and agarose gel electrophoresis.

gene	SNP	primers forward/reverse, 5’-3’	restriction enzyme
LSAMP	rs2090584	TGTAACGGCACAAACTTACAGT<br>TGTCCTTGAGCAGCAGTTTC	*Mbo*II
DAB1	rs1504589	TTCCCTGCCAGACCATATTC<br>TTCACACTTGAGCAGGATG	*Sty*I
DAB1	rs35453940	GGTGACTGGTTAAGCAGTGC<br>ACAAGAAACCGAGGCTCAGA	*Sfc*I
DAB1	rs12566928	GCCATCTTCTTCTCGCTGTG<br>CAGCCTGCATTCATCCATCC	*Psi*I
DAB1	rs79114629	CCCATTCTCACCACAGACCA<br>TCTCTCTGTGCTTGGGGATC	*Bcc*I
DAB1	rs1408138	TGCCCACATCATGCAGAGTA<br>GAATCGCTTCCTTCCGTTCC	*Xcm*I
DAB1	rs4087335	TGAGACTGCCGGCATTAAGA<br>GAGCTGCAATTTGGACCCAT	*Rsa*I

Bioinformatics pipeline

An HTML-formatted report of the pipeline code and job log files for this study, as generated by the q pipeline management utility (http://sourceforge.net/projects/q-ppln-mngr/), can be viewed at http://tewlab.path.med.umich.edu/q/Wilson_et_al_2014/pipeline.html. A description of the major analysis steps follows.

CNV cluster region identification

The first step in analyzing copy number variant (CNV) clustering was to discern any potential biases in the CNV data sets. The microarrays used provide excellent sensitivity for CNVs ~20 kb and larger and can often detect smaller CNVs in regions with dense probe spacing. The median observed CNV size was considerably larger than this limit (Table S1) and thus not skewed to larger CNVs by the detection method (Arlt et al. 2009; Arlt et al. 2011; Arlt et al. 2012; Arlt et al. 2014). CNV hotspots did not have an unusually high probe density in either human 090 fibroblasts or mouse embryonic stem (mES) cells (Table S5 and Figure S3) so that CNVs in them were not easier to detect than in the rest of the genome. mES cells did have a significantly higher median probe density in singleton CNVs, but this effect was driven by a small number of regions with a probe density no more than twice the typical genome value and thus was not considered to be a strong confounder. The clonal outgrowth required to detect CNVs might have introduced a selection bias against deleterious mutations, even though heterozygous, but this effect is expected for only a small portion of the genome and unpredictable and so could not be accounted for systematically.

We next collapsed the observed human and mouse CNV sets into overlap regions (see Figure 3A). To do this, we first removed all CNVs 2.5 Mb or larger from the sets, corresponding to 25 of 360 human CNVs (6.9%) and 8 of 377 mouse CNVs (2.1%). This step was essential because a small number of CNVs were much larger than the median size (Table S1) and their inclusion led to uninformative

grouping of CNVs into overlap regions that spanned large portions of a chromosome. Their exclusion prevented chromosome arms and similarly expansive regions from being nominated as CNV hotspots and ensured that candidate hotspots had a size scale similar the majority of the input CNVs. All CNVs, including those subjected to size exclusion, are depicted in region profile plots. The algorithm further allowed closely adjacent CNVs to be collapsed into a single cluster region, where adjacency was defined as two CNV endpoints separated by no more than 750 kb, a smaller distance than the size of many hotspots with contiguous CNV overlap. Thus, CNVs separated by a distance consistent with them being a single hotspot were considered as one region. The contribution of adjacency gaps to CNV cluster regions is tabulated in Table S7. The CNV exclusion size and adjacency allowance were determined empirically; changing them had only small effects on final results.

To determine which CNV cluster regions were non-random, we modified our previously described simulation approach (Arlt et al. 2011). The difference was that here we considered the process to proceed without replacement. Specifically, the many CNVs in highly intense and non-random cluster regions are not available for distribution throughout the rest of the genome. Thus, those CNVs should not be used to judge the randomness of less intense cluster regions. Accordingly, multiple simulation rounds were performed. In the first round, all CNVs were randomly permuted throughout the genome. Permuted CNVs were only allowed to fall within non-gap genomic regions where sufficient array probes (four and five for mouse and human, respectively) were present so that a CNV could have been detected in our experiments if it had arisen in that location. Permuted CNVs in each of 10,000 iterations were then collapsed as described above to determine how often regions randomly contained different numbers of overlapping or closely adjacent CNVs. This first round of simulation was used to generate the p-value estimates for the actual CNV region(s) containing the greatest number of CNVs. The actual CNV(s) from those highest ranking region(s) were then removed from the data set and a next simulation round performed to estimate the p-values for the next most highly clustered actual region(s). This process continued until no more CNVs remained. When done, p-values were examined as in Table S2 to determine how many CNVs in a region were required so that the probability of observing even one region with that many CNVs was <0.05. Actual clusters with this many or more CNVs were taken as validated hotpots. We also determined the CNV count for which the probability of observing as many clusters as we actually observed was <0.05.

Transcription unit identification

Bru-seq transcription intensity of genes and regions were scored using reads per kb per million reads (RPKM) units (Mortazavi et al. 2008). Profile plots show RPKM values for equally sized genome bins, with the bin size scaled to the width of the visualized window.

For transcription state calling, untreated (NT) and aphidicolin (APH)-treated Bru-seq samples for each species were combined to provide replicate input data. Genome transcription spans were initially called using a previously described hidden Markov model (HMM) segmentation algorithm in which ten discrete logarithmically distributed RPKM states (indices 0 to 9, with 9 being the most transcription) were solved for 1 kb genome bins (Paulsen et al. 2013a; Paulsen et al. 2013b). Because Bru-seq monitors nascent RNA, HMM segments included both exons and introns. Visualization of HMM segments throughout the genome revealed that segments with a state of 3 and higher could be reliably judged as having signals above background (Figure S4). Such segments were assigned a Boolean state of “transcribed”, although HMM states 1 and 2 might reflect very low levels of transcription, or transcription in only a small fraction of cells.

Contiguous transcribed segments were fused to identify preliminary transcription units (TUs), defined for this study as a contiguous genomic span undergoing active transcription in the cell type under study. This approach could not unambiguously identify transcription start sites so that some

preliminary TUs incorrectly merged what in fact were distinct adjacent units on the same genome strand. To resolve this, a splitting algorithm based on the Ensembl transcript annotation (Flicek et al. 2014) was employed in which a preliminary TU that covered at least 80% of any transcript isoform of each of two adjacent genes on the same strand was split at the most proximal transcription start site of all matching transcripts of the downstream gene, yielding final annotation-refined TU calls. Importantly, called TUs need not and typically did not correspond precisely to annotated gene boundaries, except as introduced by the splitting algorithm, but instead reflected the actual span of observed nascent transcription. Extensions past gene boundaries included the 3' run-on transcription required for mRNA polyadenylation, in addition to apparently novel transcript isoforms. Other TUs corresponded to transcribed enhancers and other phenomena.

An annotated gene was considered transcribed if 10% or more its span was occupied by TUs on the same strand. The transcribed portions of genes were obtained by splitting them against called TUs. Such splitting was inconsequential for most genes, which were wholly transcribed or untranscribed, but prevented long genes from being declared as transcribed in their entirety when only a small isoform was actually being actively expressed. On the other hand, if a long and a short isoform of a gene were both expressed, the called TU and thus the transcribed gene span reflected the longer of the overlapping isoforms.

As a nascent RNA approach, IMR-90 Gro-seq data were subjected to the same analyses as Bru-seq data, except that we found a transcribed cut-off of state 4 to be more appropriate for this sample.

Comparison to reference genome features

A series of score types were calculated for CNV regions and TUs, generically referred to as query features (Table S5). Score types represented comparisons to annotated or measured reference genome features. Most comparisons were performed without respect to strand because CNVs do not have strandedness. Boolean score types assessed whether a query feature overlapped one or more reference features. Coverage score types ("fraction in reference features") measured the fraction of the span of a query feature that overlapped reference features, thus accounting for query feature occupancy. Other score types calculated an aggregate attribute, such as average RPKM, for all reference features that were overlapped by a query feature, thereby providing information on the quality of those reference features. For all score types, adjacency gaps within CNV regions were considered to be part of the region and included in the score calculation, since we sought to characterize the properties of the entire cluster region, not just the genome bases that were actually covered by CNVs. Because of the limited number and size of gaps (Table S7) and the fact that most hotspot CNVs had both of their endpoints in the same TU (Table S6), recalculating scores with adjacency gaps excluded had minimal impact on results. Reference feature sets included the CNV regions and TUs themselves, for comparison to each other, array probe positions, and other inherent genome base properties. Publicly available reference data included the Ensembl gene annotation (Flicek et al. 2014) and Repli-seq and Repli-chip replication segments described below.

Enrichment analysis

To assess whether query features were enriched in locations with either higher or lower reference scores than expected by chance, 10,000- (CNV regions) or 1,000- (TUs) iteration simulations were performed in which all query features were randomly placed around the genome. Permuted features were required to reside on a single chromosome and, like the query features themselves, were not allowed to overlap within an iteration. CNV region placements were made without respect to strand and were restricted to locations where CNVs could have been detected by the microarrays (see above). TU placements were strand-specific and allowed in any non-gap genomic location. The actual and permuted features were scored as described above to create a table for each score type.

To generate enrichment p-value estimates from the simulation data, the mean or median score, or the percentage of positive Boolean scores, was calculated for all actual query features and for all randomly permuted features within each iteration (Table S5). Importantly, any bias introduced by variable query length was manifest equally in all iterations. For all score types, a one-tailed p-value was estimated as the fraction of aggregated iteration scores greater (or less) than the actual score or as the reciprocal of the total number of iterations if there were no such iterations. If the aggregated iteration scores were normally distributed, as judged using the Shapiro-Wilk test at a threshold of p=0.05, a preferred alternative one-tailed p-value estimate was obtained from a fit Gaussian function and reported in figures and tables. For all p-value estimates, the value is reported as "~0" when it was too small to calculate.

For most score types, all query features were used in the enrichment calculations, with a score of 0 for query features that did not overlap any reference features. However, actual and permuted query features that did not overlap a reference feature were omitted when aggregating an attribute score of the overlapped reference features. For example, estimates of enrichment for the size of reference features asked whether the query features that actually overlapped reference features were more likely to overlap large reference features than small ones.

Correlation analysis

Two approaches were used to relate reference feature enrichment to the input properties of query features, such as query feature length or region CNV count. First, Spearman correlation coefficients (r) were calculated for the input query feature score and the calculated reference score across all query features. Additionally, query features were stratified into groups based on their input scores and the enrichment analyses described above were repeated on these subsets. Grouping in this fashion allowed larger numbers of query features to be analyzed together to increase statistical power. CNV regions were stratified according to the number of CNVs they contained, as described above and in Results. TUs were stratified by length and by percentile groups of length and RPKM to facilitate comparisons of approximately equal numbers of the most deviant TUs for each inherent TU property. Statistical differences between query feature groups were calculated using a two-sample two-sided Wilcoxon test for most score types and Fisher's exact test for Boolean score types.

Repli-seq replication timing

As described in the associated publication (Hansen et al. 2010), obtained ENCODE Repli-seq data were divided into six fractions, G1, S1, S2, S3, S4 and G2, reflecting increasingly late replication times in the cell cycle. We used the percent normalized values as provided, which are the percent of sequence reads in each 1 kb genome bin assigned to each cell cycle fraction, except that we excluded from consideration any bins with fewer than 100 total reads. We then aggregated the six values for each bin into a single value reflecting the bin's consensus replication timing in an approach analogous to the ENCODE wavelet smoothed signal, except that we assigned integer states to each of the cell cycle fractions ranging from 6 (G1) to 1 (G2) and used a percentage-weighted median rather than an average across cell-cycle states prior to wavelet smoothing. This calculation was fractional such that a median falling at the boundary between two states yielded a half-state, for example, 20% G1, 30% S1, 40% S2, 5% S3, and 5% S4 gave a value of 4.5, midway between S1 (state 5) and S2 (state 4). Final replication timing values ranged from 6.0 (all signal in the G1 fraction) to 1.0 (all signal in the G2 fraction).

We next solved a six-state HMM to segment the genome according to replication timing in which bin percent-normalized values were taken as the emission probabilities and transition probabilities were set to a 0.75 probability of remaining in state, a 0.11 probability of changing to an adjacent state (e.g. from S2 to S3) and a 0.01 probability of changing to a non-adjacent state. The average replication timing over all bins in a segment yielded the consensus replication timing for that genomic span. For

merged samples, bin percent-normalized values were averaged across all input samples prior to the analyses described above. The consequences of these various manipulations are visualized in Figure S8.

Repli-chip replication timing

As described in the associated publication (Hiratani et al. 2008), obtained mES cell Repli-chip data were expressed as the replication timing ratio, which is the log2 of the normalized microarray signal for a given probe in an early S-phase sample divided by a late S-phase sample. Thus values greater and less than zero represent early and late replication, respectively. Because replication timing ratios were only defined at the position of microarray probes, we inferred the replication timing of inter-probe spans by projecting the value for a given probe to the points halfway between it and the adjacent probes on the chromosome. However, the maximum distance that a probe's value could be projected was 10 kb; probes separated by more than 20 kb resulted in genome gaps with no replication timing data. Projected probe spans were used equivalently to Repli-seq HMM segments. For transcription-replication correlation, the replication timing ratios of these segments were limited to values between 2 and -2 and rounded to bins of width 0.2, thereby creating 20 different Repli-chip segmentation states.

Transcription-replication correlation

To correlate Bru-seq/Gro-seq transcription and Repli-seq/Repli-chip replication timing values, all TUs and replication segments, excluding genome gaps and bins with insufficient Repli-seq/Repli-chip data, were split against each other at all boundaries. The length of all split segments corresponding to each of the possible transcription-replication state combinations were then summed and expressed as a percentage of the total length of all segments. To restrict this analysis to only a portion of the genome, such as CNV regions, the above boundaries were further split against the query features and only the split segments that overlapped query features were used in the comparison, with percentages expressed relative to the total length of just the query features. As appropriate, values in the resulting transcription-replication table were summed by row or by column to obtain results stratified by just transcription intensity or by replication timing. For a given set of query features, the fractional median replication timing was determined from the split segments by the same percentage-weighted approach described above, which took into account the fact that a given query feature might encompass a variety of replication states. To allow p-value calculation when comparing two sets of query features, a single replication timing value was assigned to each feature by averaging the values of all of its bins. A Wilcoxon test was then applied to the two sets of replication timing values.

Supplemental Figures

Figure S1. Additional CNV region profiles

(A) and **(B)** Exceptions to the CNV hotspot-large transcription unit relationship, as described in Results and plotted in Figure 1.
(C) to **(F)** Examples of relationship patterns between singleton CNVs and transcription units.
(G) and **(H)** The accompanying PDF files provide similar profile images for all human fibroblast and mES cell CNV regions, respectively, sorted by the number of CNVs in the region.

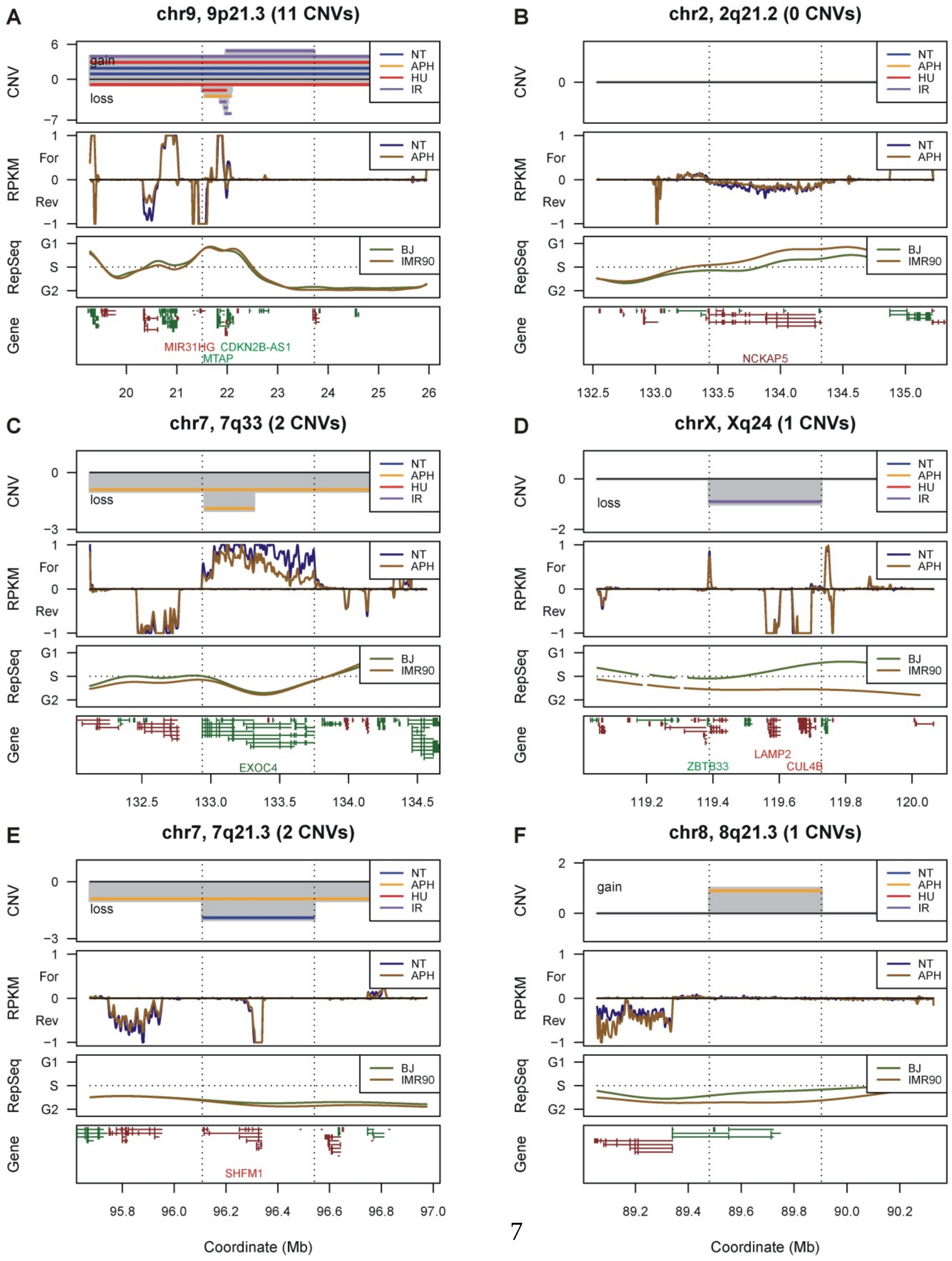

Figure S2. Common fragile sites

(A) Representative examples of CFS breaks in human fibroblasts.
(B) Human chromosome ideograms show the locations of all detected *de novo* CNVs (above) and CFS breaks and gaps (below) for 090 (red) and HF1 (blue) fibroblast cell lines.

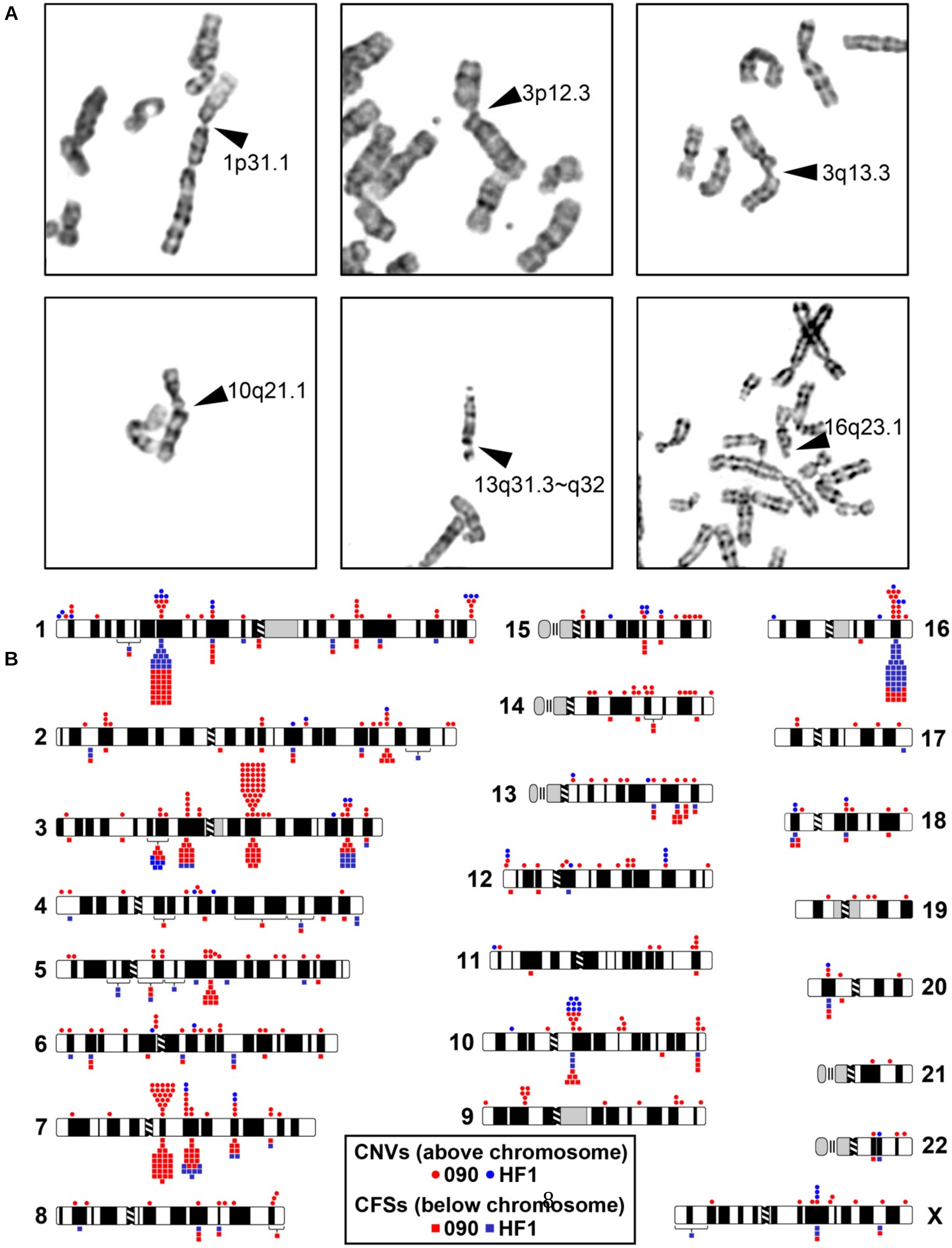

Figure S3. Additional base and gene content plots

(A) to **(G)** 090 fibroblast enrichment plots similar to Figure 3B for array probe density, CpG, G/C and simple repeat fractions, gene and large gene overlaps, and longest overlapped genes, respectively.
(H) to **(N)** The same as (A) to (G) for mES cells.

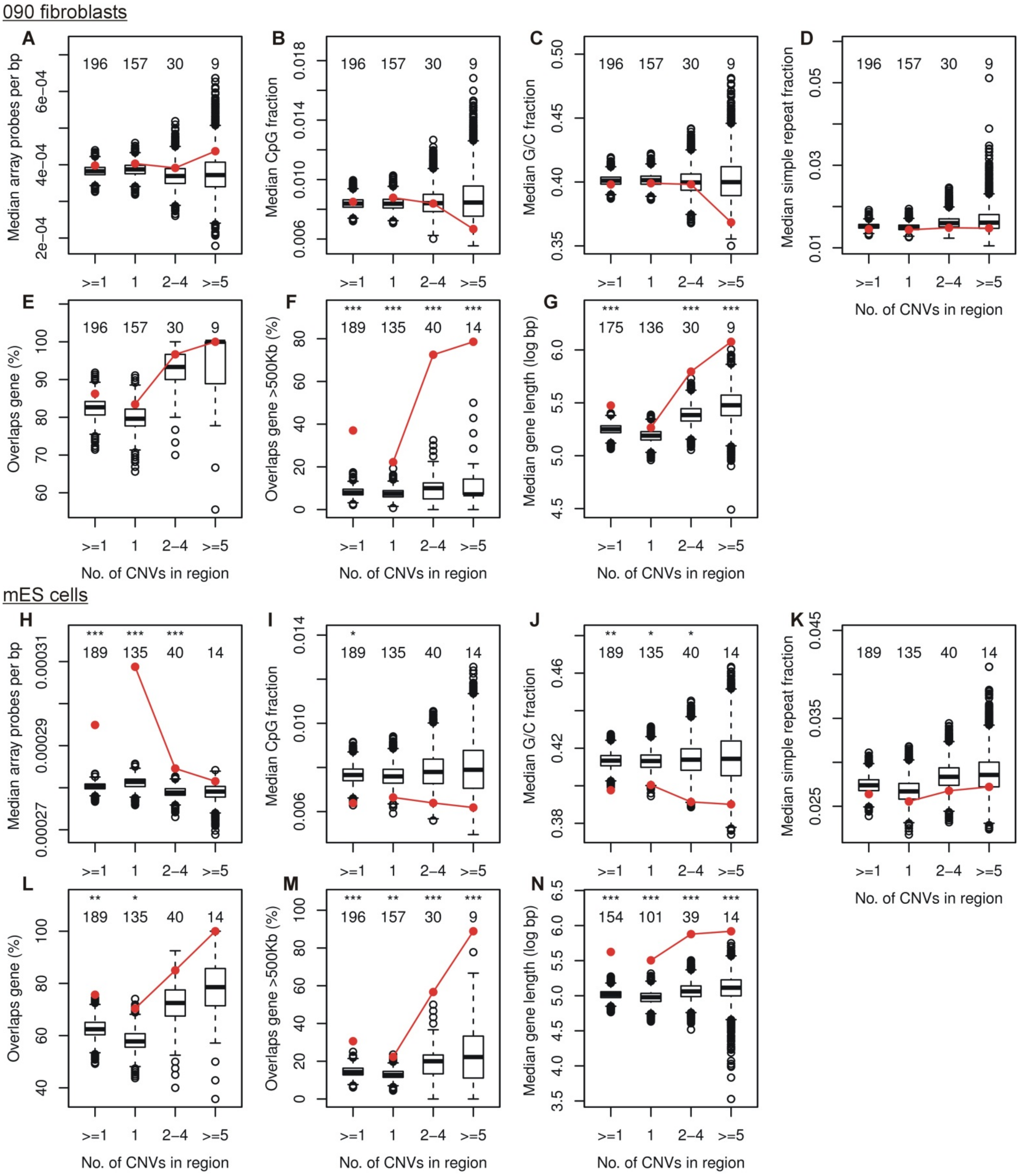

Figure S4. Bru-seq sample and state comparisons

(A) to **(C)** MA plots comparing untreated (NT) to APH-treated human 090 fibroblast Bru-seq samples, NT to APH-treated mES samples, and human 090 to HF1 fibroblasts, respectively. Each dot represents one gene. Red dots denote genes with a significant inter-sample difference as determined by DESeq (Anders and Huber 2010) at a false discovery rate of 0.05.
(D) and **(E)** Two representative ~50 kb genome regions assigned Bru-seq HMM states 2 and 3, respectively, where states 0 to 2 were considered "not transcribed" and states 3 to 9 were "transcribed". RPKM traces use a 500 bp bin size. The *MST4* gene corresponds to the state-3 TU. Dashed horizontal lines indicate the genome-wide average RPKM for transcription states 2 and 3, respectively. Increasingly unambiguous transcription was observed at HMM states above 3. Tables below summarize the fraction of the genome and Bru-seq reads that contributed to the transcribed and non-transcribed states for each cell line.

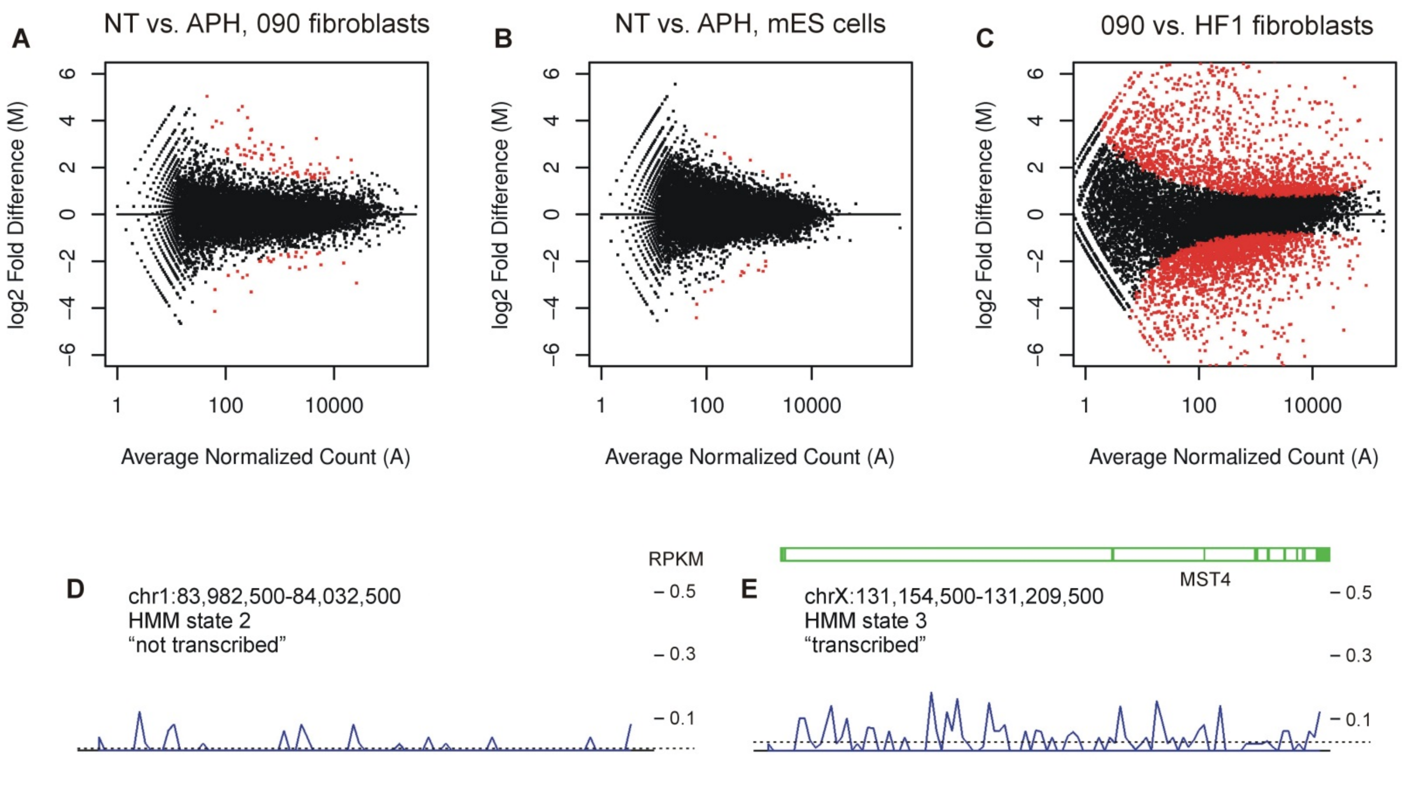

HMM state 2 or lower

	090	mES
**bp**	4.8E+09 (79%)	4.0E+09 (78%)
**reads**	5.2E+05 (0.5%)	2.5E+05 (0.6%)

HMM state 3 or higher

	090	mES
**bp**	1.8E+09 (21%)	1.9E+09 (22%)
**reads**	1.4E+06 (99%)	7.3E+05 (99%)

Figure S5. **mES cell transcription plots**

(A) to **(G)** The exact same types of transcription enrichment plots are shown here for mES cells as shown for 090 fibroblasts in Figure 4, supporting similar conclusions for both cell types.

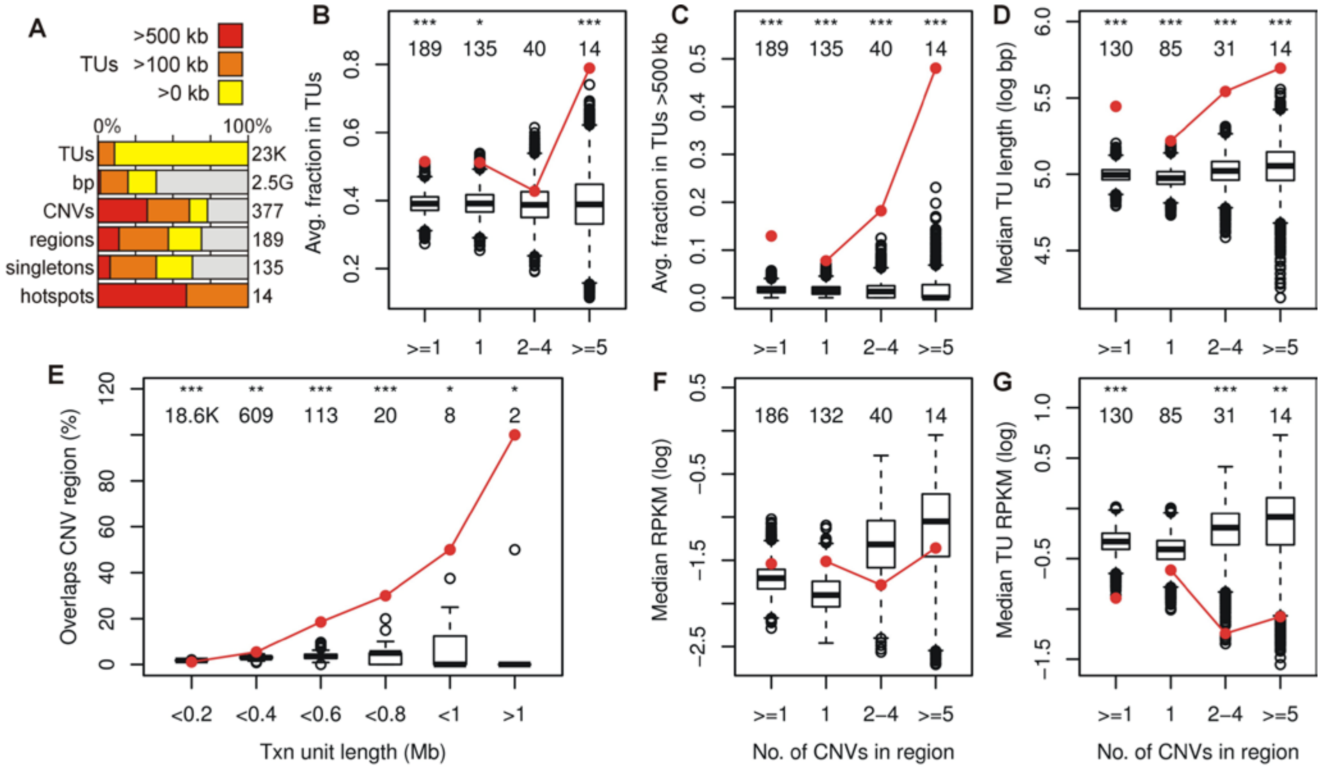

Figure S6. Additional transcription plots

(A) and **(B)** Enrichment plots for the fraction of 090 CNV regions matching the transcribed and untranscribed portions of Ensembl genes, respectively.
(C) and **(D)** Enrichment plots for the fraction of 090 TUs in CNV regions, stratified by percentile groups of TU length and RPKM, respectively. Percentile groups are mutually exclusive, e.g. a TU in group <1 was not also present in group <10.
(E) Enrichment plot assessing whether one or both of the ends of 090 CNV regions were within a TU, in contrast to other plots that compared the entire span of CNV regions.

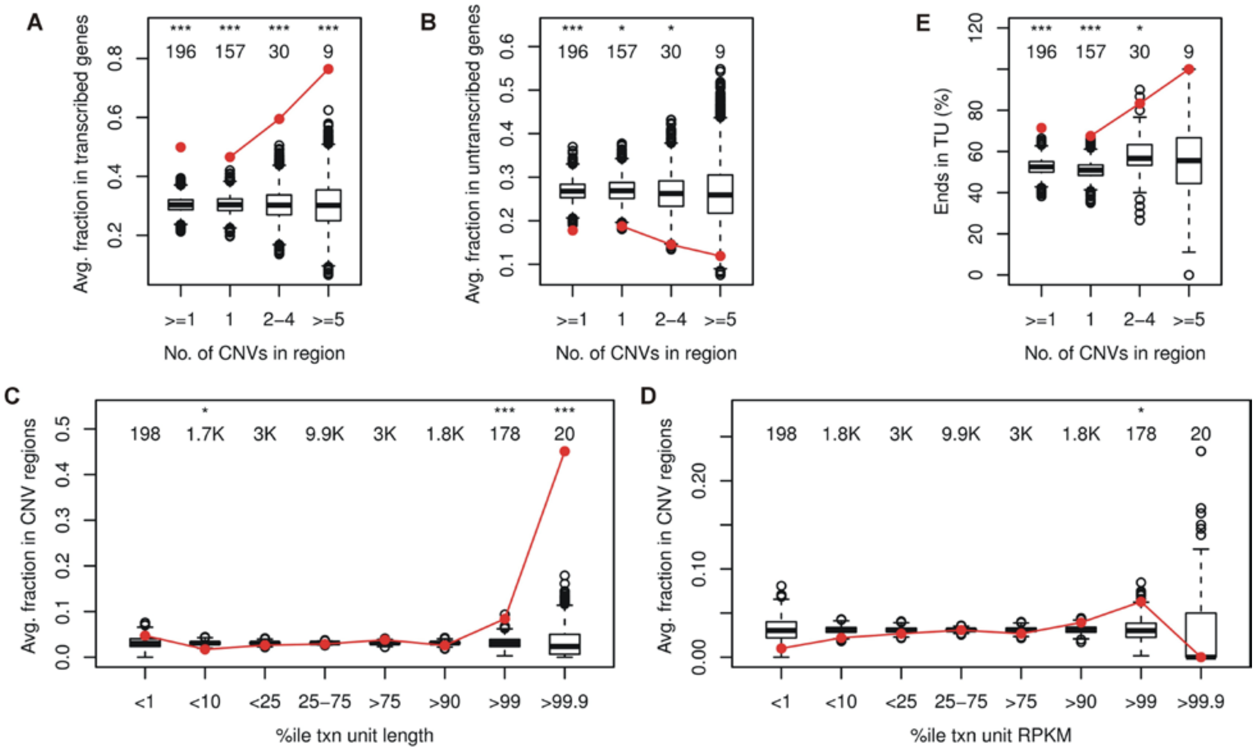

Figure S7. mES cell replication timing plots

(A) to **(F)** The exact same types of replication timing plots are shown here for mES cells as shown for human fibroblasts in Figure 6, supporting similar conclusions for both cell types. mES cell Repli-chip data are plotted as the previously described replication timing ratio, where values above and below zero represent early and late replication, respectively (Hiratani et al. 2008).

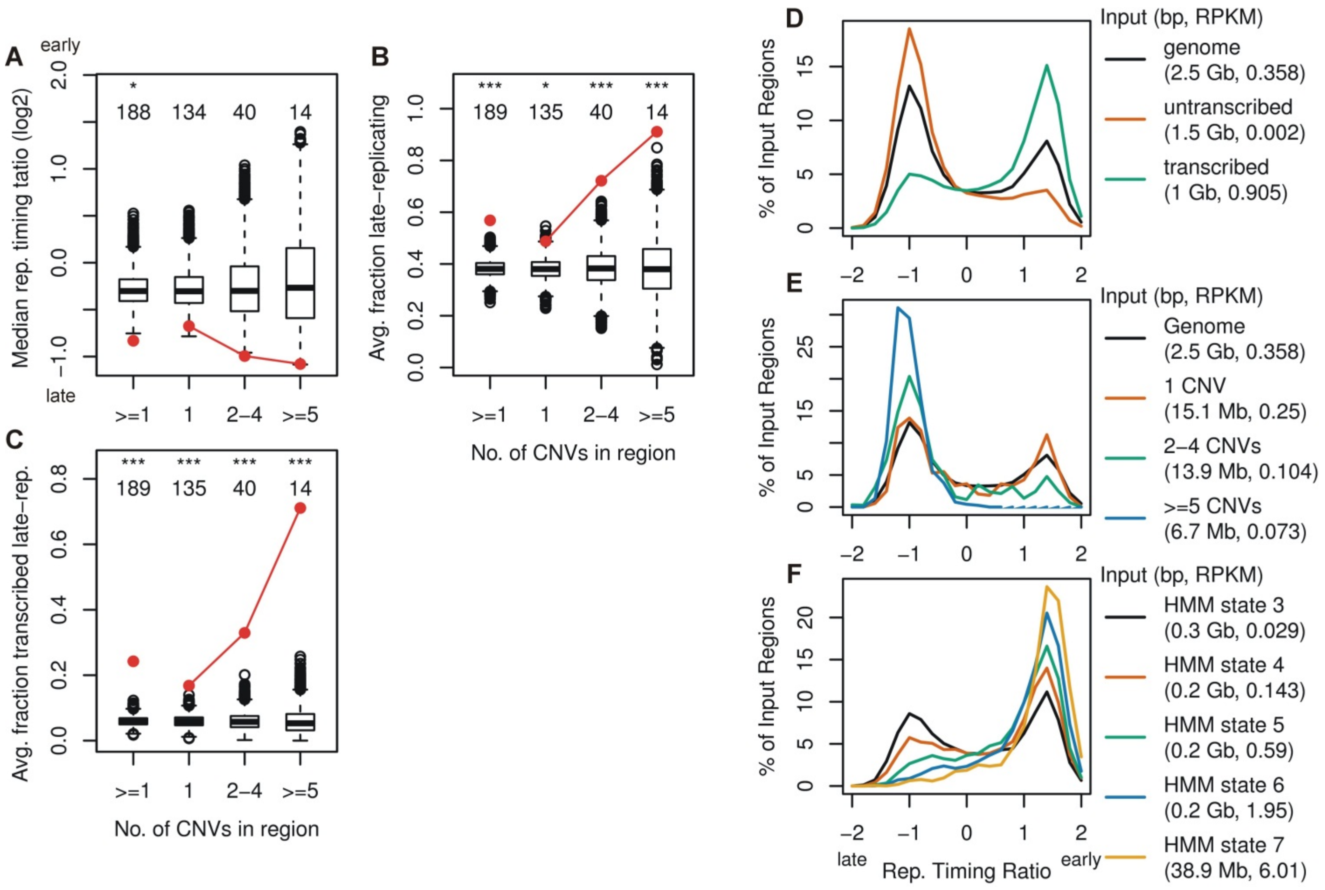

Figure S8. Additional replication timing plots

(A) Example of IMR-90 Repli-seq analysis for a 5.5 Mb span of chr5. Grey shading reflects percent normalized values for each bin/state combination, the red curve shows the calculated median replication timing, and red horizontal lines show the HMM replication segments.
(B) and **(C)** Correlation plots of 50,000 randomly selected genome bins comparing median replication timing for IMR-90 and BJ fibroblasts and GM12878 and GM06990 lymphoblastoid cells, respectively. Pearson correlation coefficients (r) are given.
(D) and **(E)** Stratification of replication timing by transcription, comparing well-matched IMR-90 Repli-seq/Gro-seq and GM12878 Repli-seq/Bru-seq sample pairs, respectively.
(F) and **(G)** Stratification of replication timing by transcription intensity, similar to (D) and (E). Results in (D) to (G) validate the IMR-90 to 090 comparisons made in Figure 6.
(H) and **(I)** Accompanying files show the relationship between transcription intensity and replication timing as a function of TU length for the IMR-90 and GM12878 sample pairs, respectively. Animation frames correspond to increasingly large TUs in 200 kb size increments. Coloring indicates the percentage of those transcription units accounted for by the combinations of replication timing and transcription intensity on the axes (red = low, yellow/white = high).

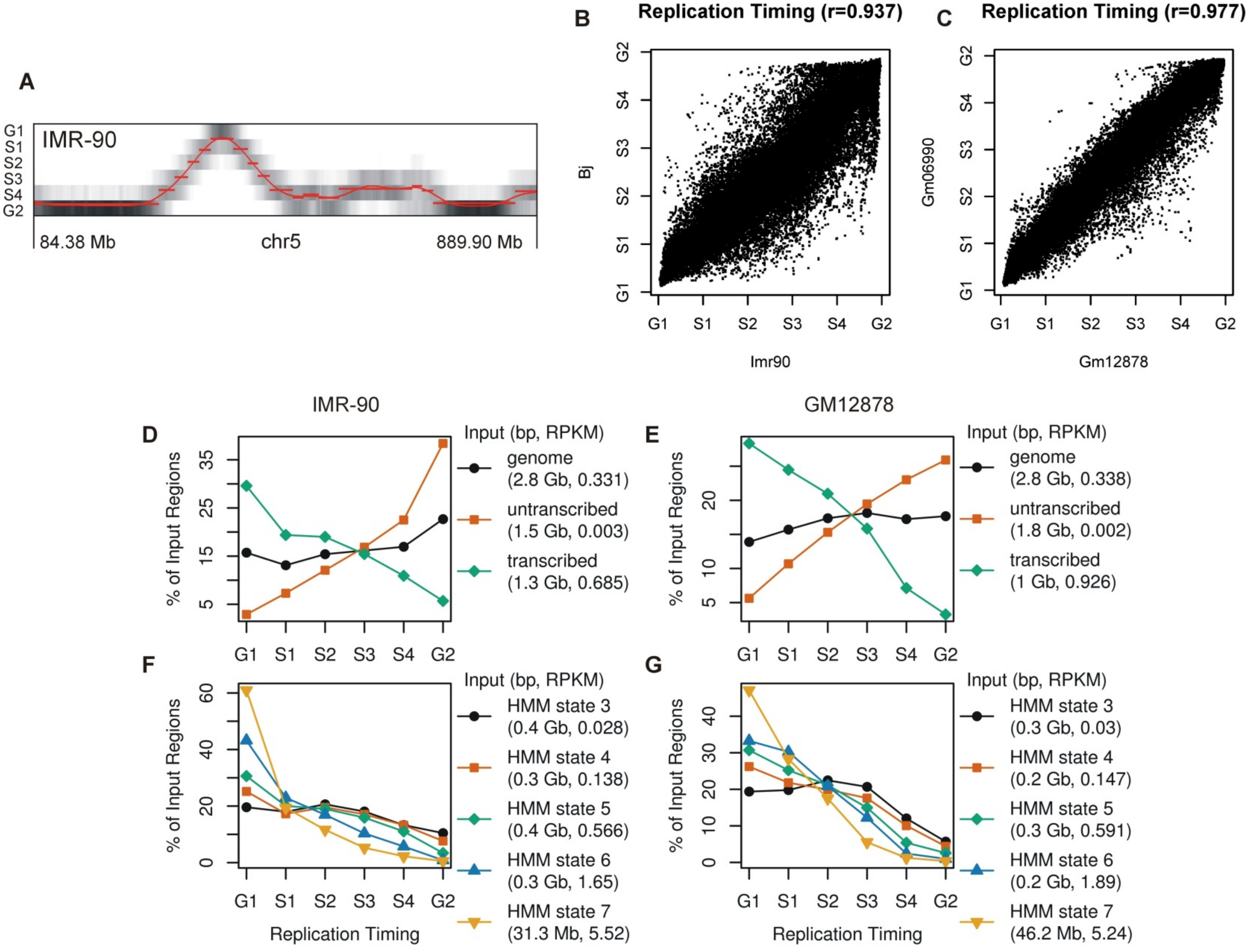

Figure S9. Late replication of large genes does not depend on transcription

(A) Example of an HF1 fibroblast nucleus that had replicated both copies of an early-replicating *C16orf45* control probe (green) but had not yet replicated an *AUTS2* hotspot probe (red).
(B) and **(C)** Summary of the percentage of replicated chromosomes for hotspot genes *LSAMP* and *AUTS2* compared to control as a measure of the relative hotspot replication timing.
(D) and **(E)** Example profile plots of large genes *CDH13* and *FHIT*, respectively, which are differentially transcribed in IMR-90 fibroblasts and GM12878 lymphoblasts.
(F) and **(G)** Average replication timing over the body of Ensembl-annotated genes >1 Mb, stratified by the longest active TU within each gene, for the IMR-90 and GM12878 cell types.
(H) Correlation of replication timing between IMR-90 and GM12878 for genes that showed differential isoform transcription (TU sizes are in the legend). Large genes replicated late whether transcribed or not, although sometimes slightly earlier when transcribed.

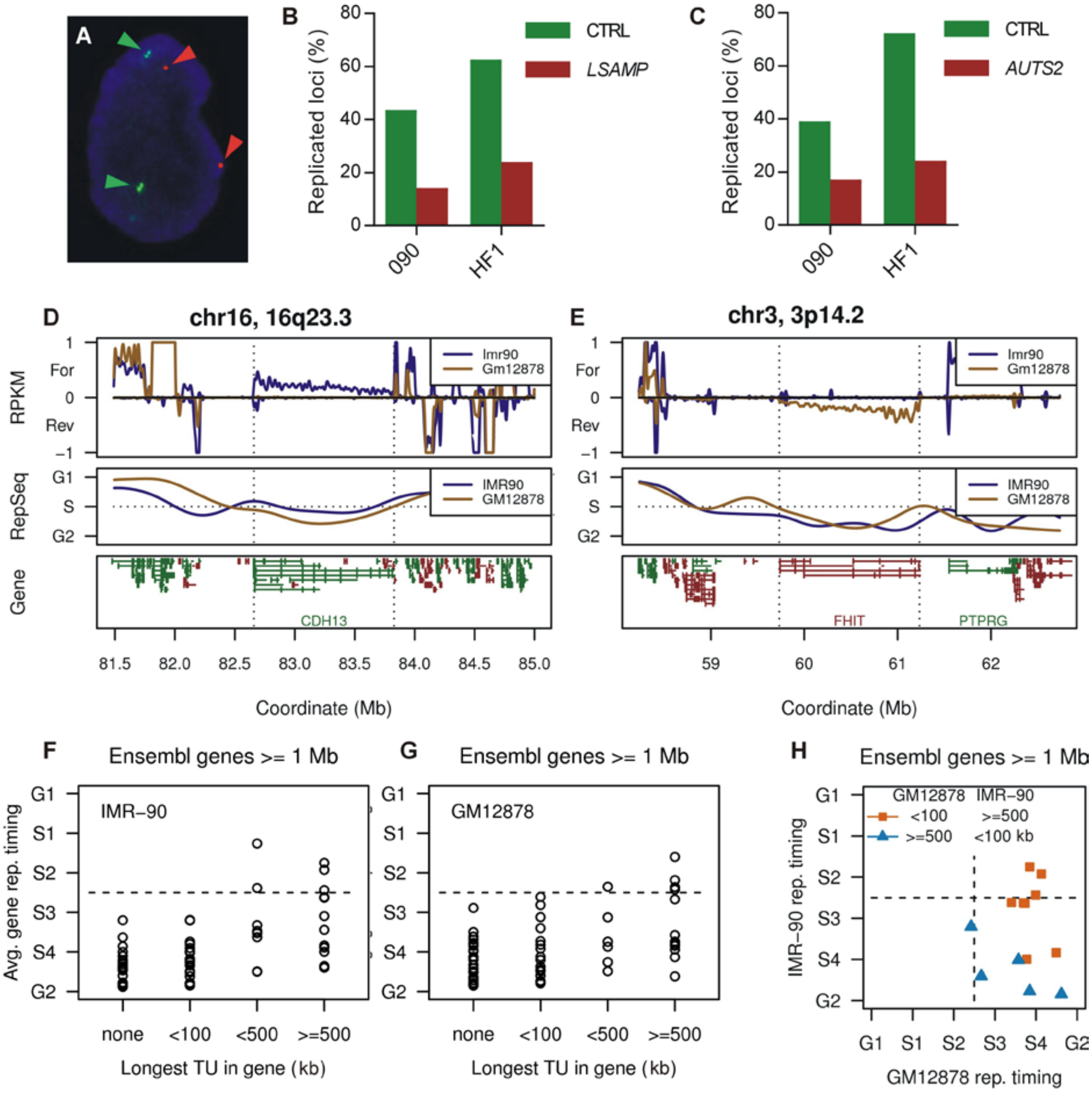

Figure S10. mES cell transcription unit alignment plots

(A) to **(F)** The exact same types of CNV region size correlation and transcription unit alignment plots are shown here for mES cells as shown for human fibroblasts in Figure 7, supporting similar conclusions for both cell types.

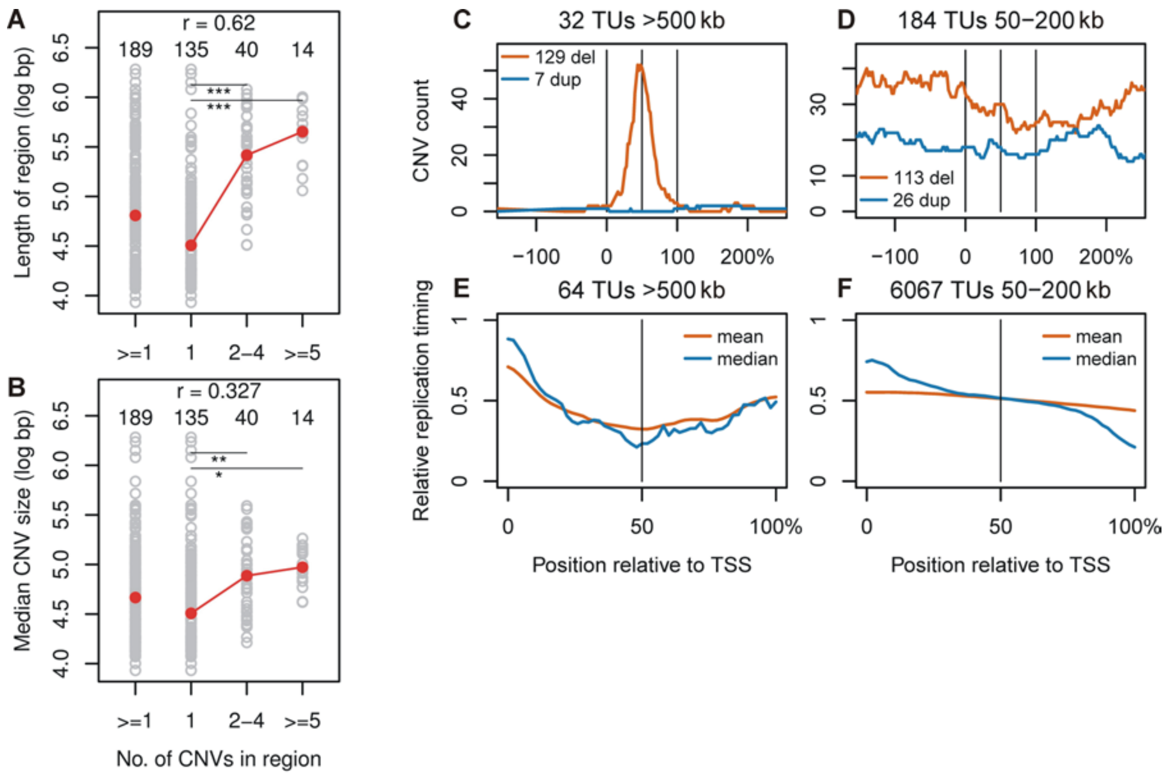

Figure S11. Additional transcription unit alignment plots

(A) and **(B)** Sum of CNV counts within and flanking TUs >500 kb for HF1 and 090 cells, respectively, omitting all IR-induced 090 CNVs.
(C) to **(E)** Mean and median relative replication timing by position for TUs >500 kb for IMR-90 Gro-seq/Repli-seq, GM12878 Bru-seq/Repli-seq, and HF1 Bru-seq/IMR-90+BJ Repli-seq, respectively.
(F) to **(H)** Median replication timing for the indicated Repli-seq (rep) and Bru-seq/Gro-seq (txn) combinations, similar to Figure 6G except that three different traces are shown for just the 10 kb at the 5' ends, middles, and 3' ends of all TUs in each size bin.

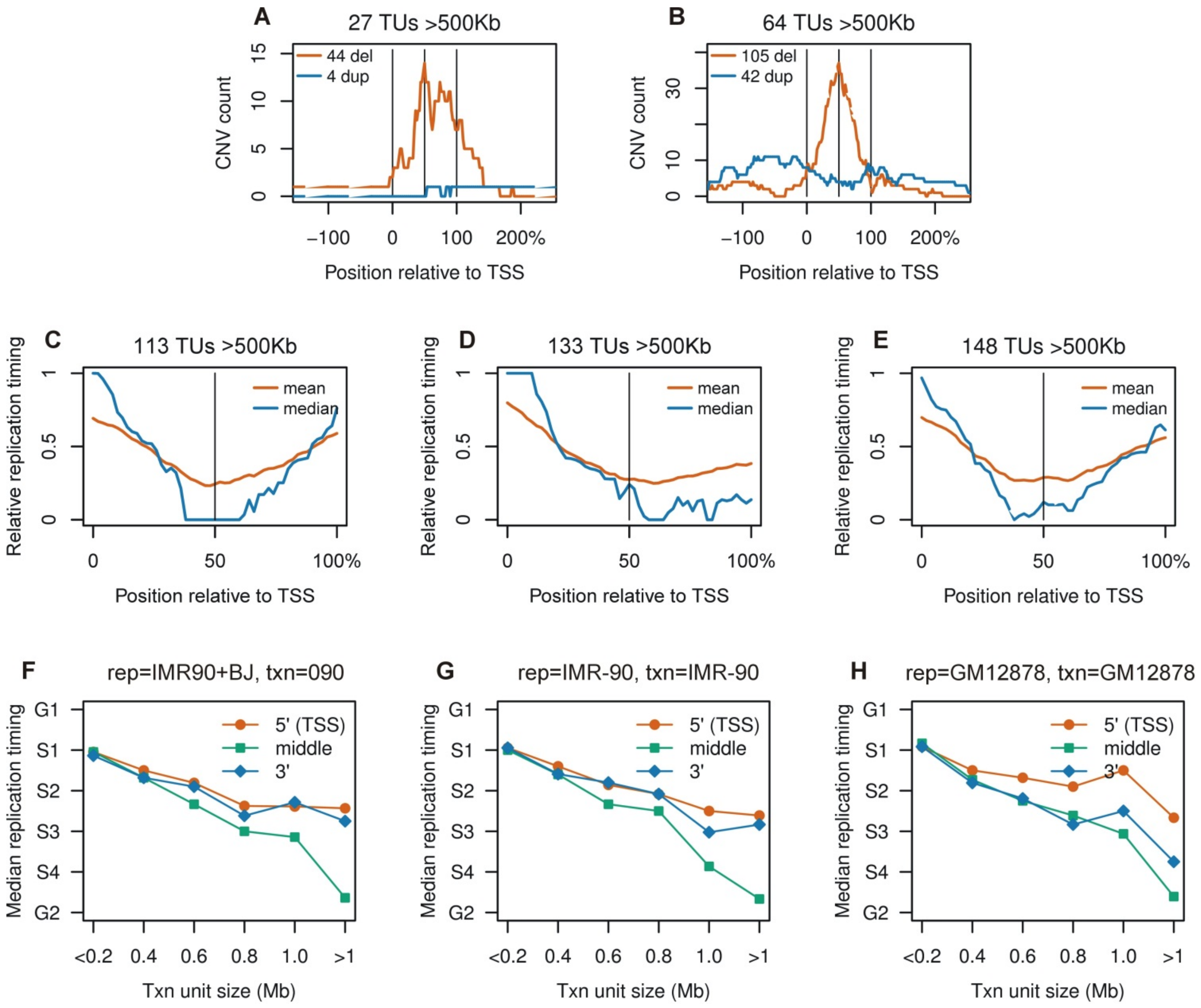

Supplemental Tables

Table S1. CNV counts and size distributions

treatment	genotype	cell clones	dups	dels	all	%dels	min (kb)	median (kb)	max (kb)
**Human 090 fibroblasts**									
**NT**[a]	**-**	52[b]	20	19	39	49%	19[c]	260	35,835
**APH**	**-**	24	13	58	71	82%	1	165	80,442
**HU**	**-**	73	51	94	145	65%	1	132	35,695
**IR**	**-**	74	55	50	105	48%	2	289	34,214
**all**		223	139	221	360	61%	1	186	80,442
**Mouse embryonic stem cells**									
**NT**	**Xrcc4$^{+/+}$**	14[d]	3	3	6	50%	13	39	1,202
**NT**	**Xrcc4$^{-/-}$**	16	8	13	21	62%	10	19	26,203
**APH**	**Xrcc4$^{+/+}$**	26	10	127	137	93%	12	62	1,418
**APH**	**Xrcc4$^{-/-}$**	29	31	182	213	85%	8	68	7,163
**all**		85	52	325	377	86%	8	63	26,203
**Human HF1 fibroblasts**									
**APH**	**-**	14	9	53	62	85%	4	103	696

[a] Abbreviations are: NT, untreated; APH, aphidicolin; HU, hydroxyurea; IR, ionizing radiation.

[b] The column lists the number of independent cell clones from which the duplication (dup) and deletion (del) CNVs were derived. Lists of individual 090 and mES CNVs are provided in (Arlt et al. 2009; Arlt et al. 2011; Arlt et al. 2012; Arlt et al. 2014). HF1 CNVs are in Table S7.

[c] The rightmost three columns indicate the smallest (min), median and largest (max) observed CNV size in kb for each row.

[d] HF1 counts only include CNVs detected by genome-wide microarray.

Table S2. CNV cluster thresholds

CNVs in region	Observed (N)	CNVs	p(>=1)[a]	p(>=N)[b]
**Human 090 fibroblasts**				
41	1	41	<0.0001[c]	<0.0001
21	1	21	<0.0001	<0.0001
9	1	9	<0.0001	<0.0001
7	2	14	0.0002	<0.0001
6	1	6	0.003	0.003
5[d]	3	15	0.0174	<0.0001
4	2	8	0.1288	0.0095
3	8	24	0.7768	0.0001
2	20	40	1	0.0402
1	157	157	1	1
**Mouse embryonic stem cells**				
32	1	32	<0.0001	<0.0001
15	1	15	<0.0001	<0.0001
14	1	14	<0.0001	<0.0001
10	1	10	<0.0001	<0.0001
9	2	18	<0.0001	<0.0001
8	1	8	<0.0001	<0.0001
7	2	14	0.0002	<0.0001
6	1	6	0.0007	0.0007
5	4	20	0.0112	<0.0001
4	3	12	0.0969	0.0002
3	11	33	0.6741	<0.0001
2	26	52	1	<0.0001
1	135	135	1	1
**Human HF1 fibroblasts**				
8	1	8	<0.0001	<0.0001
4	1	4	0.0002	0.0002
3	4	12	0.0142	<0.0001
2	9	18	0.3919	<0.0001
1	22	22	1	1

[a] p(>=1) is the frequency of simulation iterations having at least one genome region with the number of clustered CNVs indicated in column "CNVs in region".

[b] p(>=N) is the frequency of iterations having at least as many such cluster regions as the actual data, as indicated in column "Observed (N)".

[c] When no iterations yielded matching clusters, p-values are estimated as the reciprocal of the number of iterations, i.e. <1/10,000.

[d] Horizontal lines separate groups used in CNV region simulations. Definitive hotspots had p(>=1) less than 0.05, non-hotspot candidate cluster regions had p(>=N) less than 0.05.

Table S3. CNVs, genes, transcription units, and replication regions

Accompanying Excel files contain cross-referenced lists for all:

(A) CNV regions, sorted by number of CNVs.

(B) Ensembl genes >=2 kb, sorted by length.

(C) TUs, sorted by length.

(D) Human fibroblast replication segments, sorted by replication timing.

Each file includes all relevant cell types, one cell type per worksheet tab. RPKM is the Bru-seq transcription intensity across the entire region. Transcription (txn) and CNV coverage are the percentage of the region overlapped by Bru-seq TUs and CNVs, respectively. The longest TU and all genes over 500 kb overlapped by any portion of the region are listed. Further columns are CNV counts stratified by source: NT, untreated; APH, aphidicolin; HU, hydroxyurea; IR, ionizing radiation; +, Xrcc4$^{+/+}$; -, Xrcc4$^{-/-}$.

Table S4. Common fragile sites

The accompanying Excel file tabulates all CFS regions detected in this study, sorted by chromosomal coordinate. Note that although many could be confidently determined, some CFS breaks at the 7q11.2/7q21.1 and 16q23.1/16q23.3 adjacent band pairs could not be assigned with confidence depending on the quality and resolution of the chromosome banding.

Table S5. Simulation statistics

The accompanying Excel file tabulates final statistics for 10,000-iteration simulations of 090 and mES CNV regions and 1,000-iteration simulations of 090 and mES TUs for the score types described in the text. One worksheet tab is provided for each combination of cell line and feature type. Columns include: aggregate, the manner in which grouped scores were aggregated;
r, Spearman correlation coefficients of raw scores of all CNV regions vs. the number of CNVs they contained, or all TUs vs. their length. Rightmost columns show results for CNV region subsets grouped by the number of CNVs they contained or TU subsets grouped by length, in format “actual aggregate score (p-value)”. Groups are mutually exclusive, e.g. length group “<0.4Mb” does not include TUs in group “<0.2Mb”.

Table S6. Transcription unit overlaps

TU overlap type[b]	TU length[a] NA	>=500 kb	>=100 kb	>0 kb	row total
**090 fibroblast singleton CNVs**					
none	34 (22%)	-	-	-	34 (22%)
both ends in same TU	-	17 (11%)	21 (13%)	5 (3%)	43 (27%)
two ends in different TUs	-	4 (3%)	27 (17%)	7 (4%)	38 (24%)
one end in a TU	-	4 (3%)	10 (6%)	11 (7%)	25 (16%)
spans one TU	-	-	2 (1%)	7 (4%)	9 (6%)
spans multiple TUs	-	-	6 (4%)	2 (1%)	8 (5%)
	34 (22%)	25 (16%)	66 (42%)	32 (20%)	157 (100%)
**090 fibroblast regions with 2-4 CNVs**					
none	1 (3%)	-	-	-	1 (3%)
both ends in same TU	-	3 (10%)	1 (3%)	-	4 (13%)
two ends in different TUs	-	8 (27%)	3 (10%)	-	11 (37%)
one end in a TU	-	5 (17%)	3 (10%)	2 (7%)	10 (33%)
spans one TU	-	-	-	1 (3%)	1 (3%)
spans multiple TUs	-	1 (3%)	2 (7%)	-	3 (10%)
	1 (3%)	17 (57%)	9 (30%)	3 (10%)	30 (100%)
**090 fibroblast CNVS in regions with 2-4 CNVs**					
none	7 (10%)	-	-	-	7 (10%)
both ends in same TU	-	20 (28%)	6 (8%)	-	26 (36%)
two ends in different TUs	-	8 (11%)	9 (13%)	2 (3%)	19 (26%)
one end in a TU	-	6 (8%)	3 (4%)	7 (10%)	16 (22%)
spans one TU	-	-	-	1 (1%)	1 (1%)
spans multiple TUs	-	-	2 (3%)	1 (1%)	3 (4%)
	7 (10%)	34 (47%)	20 (28%)	11 (15%)	72 (100%)
**090 fibroblast regions with >=5 CNVs**					
none	-	-	-	-	-
both ends in same TU	-	1 (11%)	-	-	1 (11%)
two ends in different TUs	-	2 (22%)	-	-	2 (22%)
one end in a TU	-	5 (56%)	1 (11%)	-	6 (67%)
spans one TU	-	-	-	-	-
spans multiple TUs	-	-	-	-	-
	-	8 (89%)	1 (11%)	-	9 (100%)
**090 fibroblast CNVS in regions with >=5 CNVs**					
none	2 (2%)	-	-	-	2 (2%)
both ends in same TU	-	84 (79%)	1 (1%)	-	85 (80%)
two ends in different TUs	-	3 (3%)	4 (4%)	-	7 (7%)
one end in a TU	-	10 (9%)	2 (2%)	-	12 (11%)
spans one TU	-	-	-	-	-
spans multiple TUs	-	-	-	-	-
	2 (2%)	97 (92%)	7 (7%)	-	106 (100%)
**mES cell singleton CNVs**					
none	50 (37%)	-	-	-	50 (37%)
both ends in same TU	-	10 (7%)	31 (23%)	7 (5%)	48 (36%)
two ends in different TUs	-	1 (1%)	2 (1%)	8 (6%)	11 (8%)
one end in a TU	-	-	6 (4%)	15 (11%)	21 (16%)
spans one TU	-	-	-	3 (2%)	3 (2%)
spans multiple TUs	-	-	2 (1%)	-	2 (1%)

TU overlap type[b]	TU length[a] NA	>=500 kb	>=100 kb	>0 kb	row total
	50 (37%)	11 (8%)	41 (30%)	33 (24%)	135 (100%)
**mES cell regions with 2-4 CNVs**					
none	9 (23%)	-	-	-	9 (23%)
both ends in same TU	-	6 (15%)	4 (10%)	-	10 (25%)
two ends in different TUs	-	1 (3%)	2 (5%)	-	3 (8%)
one end in a TU	-	2 (5%)	6 (15%)	1 (3%)	9 (23%)
spans one TU	-	-	-	5 (13%)	5 (13%)
spans multiple TUs	-	-	2 (5%)	2 (5%)	4 (10%)
	9 (23%)	9 (23%)	14 (35%)	8 (20%)	40 (100%)
**mES cell CNVS in regions with 2-4 CNVs**					
none	42 (43%)	-	-	-	42 (43%)
both ends in same TU	-	19 (20%)	16 (16%)	-	35 (36%)
two ends in different TUs	-	-	2 (2%)	-	2 (2%)
one end in a TU	-	4 (4%)	6 (6%)	3 (3%)	13 (13%)
spans one TU	-	-	-	3 (3%)	3 (3%)
spans multiple TUs	-	-	1 (1%)	1 (1%)	2 (2%)
	42 (43%)	23 (24%)	25 (26%)	7 (7%)	97 (100%)
**mES cell regions with >=5 CNVs**					
none	-	-	-	-	-
both ends in same TU	-	6 (43%)	1 (7%)	-	7 (50%)
two ends in different TUs	-	1 (7%)	1 (7%)	-	2 (14%)
one end in a TU	-	-	4 (29%)	-	4 (29%)
spans one TU	-	-	-	-	-
spans multiple TUs	-	-	1 (7%)	-	1 (7%)
	-	7 (50%)	7 (50%)	-	14 (100%)
**mES cell CNVS in regions with >=5 CNVs**					
none	9 (7%)	-	-	-	9 (7%)
both ends in same TU	-	86 (63%)	24 (18%)	-	110 (80%)
two ends in different TUs	-	2 (1%)	3 (2%)	-	5 (4%)
one end in a TU	-	-	10 (7%)	-	10 (7%)
spans one TU	-	-	-	1 (1%)	1 (1%)
spans multiple TUs	-	-	-	2 (1%)	2 (1%)
	9 (7%)	88 (64%)	37 (27%)	3 (2%)	137 (100%)

[a] Length group of the longest TU overlapped by a CNV or CNV region. NA, not applicable, i.e. the CNV or region did not overlap a gene. Groups are mutually exclusive, e.g. group “>=100 kb” does not include CNV regions in group “>=500 kb”.

[b] TU overlap types indicate whether or one or both ends of a CNV or CNV region fell within a TU, or whether the region instead spanned (i.e. contained) one or more TUs.

Table S7. Gaps in CNV cluster regions

CNV region	CNVs	gaps	size (kb) region	size (kb) largest gap	gap contained in one[a] gene	gap contained in one[a] TU
**Human 090 fibroblasts (18 of 39 regions with >1 CNV)**						
chr10:52699410-53983384	7	3	1284	210	yes,yes,yes	yes,yes,yes
chr1:71870126-72933163	7	2	1063	116	yes,yes	yes,yes
chr1:245413423-246284662	5	1	871	27	yes	yes
chr3:76299612-77188041	5	1	888	98	yes	yes
chr1:173615117-175294275	4	2	1679	738	no,yes	no,yes
chr3:174018217-175087884	4	2	1070	632	yes,yes	yes,yes
chr11:126153842-126788587	3	1	635	428	no	no
chr16:83212397-83993943	3	2	782	230	yes,yes	yes,yes
chr2:205121597-206342082	3	1	1220	356	yes	yes
chr2:36626077-36756196	3	1	130	19	yes	yes
chr6:56077916-57305875	3	1	1228	125	no	no
chr8:139618753-142414326	3	1	2796	8	no	no
chr12:1232370-1634168	2	1	402	7	yes	yes
chr14:50087752-50934205	2	1	846	744	no	no
chr20:14764446-15435564	2	1	671	577	yes	yes
chr3:186976990-188315629	2	1	1339	307	no	no
chr5:58535683-59371938	2	1	836	618	yes	yes
chr6:101049107-102037684	2	1	989	194	no	no
**Mouse embryonic stem cells (28 of 54 regions with >1 CNV)**						
chr2:140550330-141557233	9	1	1007	10	yes	yes
chr12:38642600-39183139	5	2	541	54	yes,yes	yes,yes
chr15:32274448-33241562	5	1	967	716	no	no
chr15:29596756-30675834	4	2	1079	310	yes,yes	yes,yes
chr3:118447467-118817178	4	1	370	88	yes	yes
chr12:42266930-42912024	3	1	645	383	yes	yes
chr3:25419264-25924125	3	1	505	292	yes	yes
chr4:75876533-75983897	3	1	107	5	yes	yes
chr7:56946520-57924682	3	1	978	730	no	no
chr9:68997130-70201450	3	1	1204	627	no	no
chr10:89667208-89923292	2	1	256	78	yes	yes
chr1:21636785-21828953	2	1	192	47	yes	yes
chr1:25406391-25729301	2	1	323	125	yes	yes
chr12:93372248-93437491	2	1	65	11	no	no
chr14:12657349-12726335	2	1	69	13	yes	yes
chr14:23212889-23350017	2	1	137	24	yes	yes
chr15:73864584-74363904	2	1	499	467	no	no
chr1:62048401-62126441	2	1	78	28	yes	yes
chr16:97041506-97307131	2	1	266	182	yes	yes
chr17:59643453-60460645	2	1	817	38	no	no
chr17:63108554-63466468	2	1	358	298	no	no

CNV region	CNVs	gaps	size (kb) region	size (kb) largest gap	gap contained in one[a] gene	gap contained in one[a] TU
chr2:14710180-14825201	2	1	115	48	yes	yes
chr2:178382089-179172258	2	1	790	313	no	no
chr3:156168240-156326627	2	1	158	92	no	no
chr6:63397825-63872019	2	1	474	356	yes	yes
chr8:79660047-79803051	2	1	143	16	yes	yes
chr9:96739498-97599236	2	1	860	340	no	no
chrX:69967118-70013398	2	1	46	9	no	no

[a] The table lists all human and mouse CNV regions that contained one or more CNV adjacency gaps. The rightmost columns indicates for each gap whether or not that gap was completely contained in a single gene or TU; if "yes", the gap was within the same gene or TU as each of its two flanking CNVs.

Table S8. Human HF1 fibroblast CNVs

chrom	start	end	kb	method	probes	copies	type	band
1	1527083	1733219	206	aCGH	115	3	gain	1p36.33
1	3518415	3522561	4	aCGH	12	3	gain	1p36.32
1	8961863	9556061	594	aCGH	818	3	gain	1p36.22
1	58048797	58225373	177	RFLP	4	1	loss	1p32.2
1	58138896	58148455	10	RFLP	2	1	loss	1p32.2
1	72088017	72113244	25	aCGH	17	1	loss	1p31.1
1	72239276	72352237	113	aCGH	74	1	loss	1p31.1
1	72326978	72352237	25	aCGH	16	1	loss	1p31.1
1	72401185	72637154	236	aCGH	184	1	loss	1p31.1
1	98035253	98160717	125	aCGH	116	1	loss	1p21.3
1	98117882	98149330	31	aCGH	25	1	loss	1p21.3
1	245742124	246118050	376	aCGH	564	1	loss	1q44
1	245871108	246084652	214	aCGH	320	0	loss	1q44
1	245914673	245942059	27	aCGH	49	1	loss	1q44
2	142374952	142405801	31	aCGH	50	1	loss	2q22.2
2	149055476	149161514	106	aCGH	64	3	gain	2q23.1
2	206256232	206301132	45	aCGH	38	1	loss	2q33.3
3	165940003	166065099	125	aCGH	72	3	gain	3q26.1
3	173689779	174385622	696	aCGH	579	1	loss	3q26.31
3	174813530	174831994	18	aCGH	35	1	loss	3q26.31
4	86874894	87190305	315	aCGH	200	1	loss	4q23.1
4	87119224	87460112	341	aCGH	202	1	loss	4q21.3
6	56436335	56643026	207	aCGH	152	1	loss	6p12.1
6	86295429	86523508	228	aCGH	84	1	loss	6q14.3
7	78231922	78575481	344	aCGH	329	1	loss	7q21.11
7	78547842	78569021	21	aCGH	14	1	loss	7q21.11
7	110656251	110707029	51	aCGH	33	1	loss	7q31.1
7	110665997	110684216	18	aCGH	19	1	loss	7q31.1
10	13715421	13744567	29	aCGH	66	1	loss	10p13
10	53223446	53348048	125	aCGH	147	1	loss	10q21.1
10	53268580	53504174	236	aCGH	233	1	loss	10q21.1
10	53269467	53292516	23	aCGH	26	1	loss	10q21.1
10	53336425	53435774	99	aCGH	88	1	loss	10q21.1
10	53376213	53408296	32	aCGH	33	1	loss	10q21.1
10	53388223	53407270	19	aCGH	19	0	loss	10q21.1
10	53463410	53534471	71	aCGH	70	1	loss	10q21.1
10	53875195	53914073	39	aCGH	35	3	gain	10q21.1
11	1075747	1083946	8	aCGH	13	3	gain	11p15.5
12	1067895	1236729	169	aCGH	129	1	loss	12p13.33
12	1493559	1684076	191	aCGH	294	1	loss	12p13.33
12	44573740	44743021	169	aCGH	77	1	loss	12q12
12	99765812	100058839	293	aCGH	253	1	loss	12q23.1
12	99802508	100220452	418	aCGH	353	1	loss	12q23.1
12	99934156	100262533	328	aCGH	261	1	loss	12q23.1
13	21563311	21575612	12	aCGH	9	1	loss	13q12.11
13	73373325	73390447	17	aCGH	10	1	loss	13q22.1
15	42895210	42941759	47	aCGH	18	1	loss	15q15.2
15	60972325	61088810	116	aCGH	164	1	loss	15q22.2
15	61013134	61021909	9	aCGH	16	0	loss	15q22.2

chrom	start	end	kb	method	probes	copies	type	band
15	60983429	61130639	147	aCGH	206	1	loss	15q22.2
15	71908678	72038676	130	aCGH	147	1	loss	15q23
16	2257374	2262987	6	aCGH	14	3	gain	16p13.3
16	72598884	72696237	97	aCGH	32	1	loss	16q22.2
16	78604831	78689352	85	aCGH	186	1	loss	16q23.1
16	83041537	83247237	206	aCGH	435	1	loss	16q23
16	83165910	83261615	96	aCGH	194	1	loss	16q23.3
18	7859159	7877052	18	aCGH	16	1	loss	18p11.23
18	8101814	8116625	15	aCGH	11	1	loss	18p11.23
18	34345684	34992205	647	aCGH	398	3	gain	18q12.2
20	14568758	14702440	134	aCGH	117	1	loss	20p12.1
22	33922837	33994118	71	aCGH	94	1	loss	22q12.3
X	95963620	96075055	111	aCGH	57	0	loss	Xq21.33
X	96416258	96443397	27	aCGH	27	0	loss	Xq23.33
X	96591587	96793675	202	aCGH	60	0	loss	Xq21.33

Table S9. CNVs in larges genes in human genomic disease and cancer

gene[a]	size (Mb)[b]	*de novo* CNVs in this study	*de novo* CNVs in human genomic disease and cancer[c]	references
**matching long transcription unit in 090 or HF1 fibroblasts[d]**				
LSAMP	2.2	41	osteosarcoma	(Pasic et al. 2010)
MACROD2	2.1	3	major depressive disorder, multiple cancers	(Perlis et al. 2012; Zack et al. 2013)
PDE4D	1.6	2	esophageal adenocarcinoma	(Nancarrow et al. 2008)
AUTS2	1.2	19	ASD, developmental delay	(Mefford et al. 2010; Beunders et al. 2013; Nagamani et al. 2013)
WWOX	1.1	10	multiple cancers	(Beroukhim et al. 2010; Zack et al. 2013)
IMMP2L	0.9	5	Alzheimer's disease, ASD	(Maestrini et al. 2010; Swaminathan et al. 2012)
NEGR1	0.9	11	childhood obesity, cancers, dyslexia	(Jarick et al. 2011; Veerappa et al. 2013; Zack et al. 2013)
MAGI1	0.7	2	bipolar affective disorder, schizophrenia	(Karlsson et al. 2012)
**matching long transcription unit in mES cells only [e]**				
PTPRD	2.3	4	ADHD, hepatocellular carcinoma, esophageal adenocarcinoma	(Beroukhim et al. 2010; Elia et al. 2010; Nalesnik et al. 2012)
FHIT	1.5	5	ASD, multiple cancers	(Beroukhim et al. 2010; Girirajan et al. 2013; Zack et al. 2013)
NRXN1	1.1	7	ID, ASD, schizophrenia	(Gregor et al. 2011; Tucker et al. 2013)
NLGN1	0.9	3	ASD, ID	(Glessner et al. 2009)
**not transcribed in cell lines in this study[d]**				
CNTNAP2	2.3	0	schizophrenia, autism, epilepsy, ADHD, dyslexia, multiple cancers	(Friedman et al. 2008; Elia et al. 2010; Mikhail et al. 2011; Veerappa et al. 2013; Zack et al. 2013)
DMD	2.2	0	Duchene muscular dystrophy, multiple cancers	(White and den Dunnen 2006; Beroukhim et al. 2010; Mitsui et al. 2010; Zack et al. 2013)
DLG2	2.2	0	ID, multiple congenital abnormalities	(Vulto-van Silfhout et al. 2013)
NRXN3	1.6	0	ASD	(Vaags et al. 2012)
PARK2	1.4	0	Parkinsonism, ASD, multiple cancers	(Glessner et al. 2009; Mitsui et al. 2010; Wang et al. 2013; Zack et al. 2013)
GRID1	0.8	0	ASD	(Glessner et al. 2009)

[a] The table provides examples of genes with human disease-associated CNVs and is not inclusive.

[b] Gene sizes are from the Ensembl annotation.
[c] Abbreviations: ASD, autism spectrum disorder; ADHD, attention deficit hyperactivity disorder; ID, intellectual disability.
[d] CNV counts are from combined 090 and HF1 fibroblast data.
[e] CNV counts are from mES cell data. No CNVs were found at these loci in 090 and HF1 cells.

Supplemental References

Anders S, Huber W. 2010. Differential expression analysis for sequence count data. *Genome Biol* **11**(10): R106.

Arlt MF, Mulle JG, Schaibley VM, Ragland RL, Durkin SG, Warren ST, Glover TW. 2009. Replication stress induces genome-wide copy number changes in human cells that resemble polymorphic and pathogenic variants. *Am J Hum Genet* **84**(3): 339-350.

Arlt MF, Ozdemir AC, Birkeland SR, Wilson TE, Glover TW. 2011. Hydroxyurea induces de novo copy number variants in human cells. *Proc Natl Acad Sci U S A* **108**(42): 17360-17365.

Arlt MF, Rajendran S, Birkeland SR, Wilson TE, Glover TW. 2012. De novo CNV formation in mouse embryonic stem cells occurs in the absence of Xrcc4-dependent nonhomologous end joining. *PLoS Genet* **8**(9): e1002981.

-. 2014. Copy number variants are produced in response to low-dose ionizing radiation in cultured cells. *Environ Mol Mutagen* **55**(2): 103-113.

Beroukhim R, Mermel CH, Porter D, Wei G, Raychaudhuri S, Donovan J, Barretina J, Boehm JS, Dobson J, Urashima M et al. 2010. The landscape of somatic copy-number alteration across human cancers. *Nature* **463**(7283): 899-905.

Beunders G, Voorhoeve E, Golzio C, Pardo LM, Rosenfeld JA, Talkowski ME, Simonic I, Lionel AC, Vergult S, Pyatt RE et al. 2013. Exonic deletions in AUTS2 cause a syndromic form of intellectual disability and suggest a critical role for the C terminus. *Am J Hum Genet* **92**(2): 210-220.

Elia J, Gai X, Xie HM, Perin JC, Geiger E, Glessner JT, D'Arcy M, deBerardinis R, Frackelton E, Kim C et al. 2010. Rare structural variants found in attention-deficit hyperactivity disorder are preferentially associated with neurodevelopmental genes. *Mol Psychiatry* **15**(6): 637-646.

Flicek P, Amode MR, Barrell D, Beal K, Billis K, Brent S, Carvalho-Silva D, Clapham P, Coates G, Fitzgerald S, et al. 2014. Ensembl 2014. *Nucleic Acids Res*. **42**(Database issue):D749-55.

Friedman JI, Vrijenhoek T, Markx S, Janssen IM, van der Vliet WA, Faas BH, Knoers NV, Cahn W, Kahn RS, Edelmann L et al. 2008. CNTNAP2 gene dosage variation is associated with schizophrenia and epilepsy. *Mol Psychiatry* **13**(3): 261-266.

Girirajan S, Dennis MY, Baker C, Malig M, Coe BP, Campbell CD, Mark K, Vu TH, Alkan C, Cheng Z et al. 2013. Refinement and discovery of new hotspots of copy-number variation associated with autism spectrum disorder. *Am J Hum Genet* **92**(2): 221-237.

Glessner JT, Wang K, Cai G, Korvatska O, Kim CE, Wood S, Zhang H, Estes A, Brune CW, Bradfield JP et al. 2009. Autism genome-wide copy number variation reveals ubiquitin and neuronal genes. *Nature* **459**(7246): 569-573.

Gregor A, Albrecht B, Bader I, Bijlsma EK, Ekici AB, Engels H, Hackmann K, Horn D, Hoyer J, Klapecki J et al. 2011. Expanding the clinical spectrum associated with defects in CNTNAP2 and NRXN1. *BMC Med Genet* **12**: 106.

Hansen RS, Thomas S, Sandstrom R, Canfield TK, Thurman RE, Weaver M, Dorschner MO, Gartler SM, Stamatoyannopoulos JA. 2010. Sequencing newly replicated DNA reveals widespread plasticity in human replication timing. *Proc Natl Acad Sci U S A* **107**(1): 139-144.

Hiratani I, Ryba T, Itoh M, Yokochi T, Schwaiger M, Chang CW, Lyou Y, Townes TM, Schubeler D, Gilbert DM. 2008. Global reorganization of replication domains during embryonic stem cell differentiation. *PLoS Biol* **6**(10): e245.

Jarick I, Vogel CI, Scherag S, Schafer H, Hebebrand J, Hinney A, Scherag A. 2011. Novel common copy number variation for early onset extreme obesity on chromosome 11q11 identified by a genome-wide analysis. *Hum Mol Genet* **20**(4): 840-852.

Karlsson R, Graae L, Lekman M, Wang D, Favis R, Axelsson T, Galter D, Belin AC, Paddock S. 2012. MAGI1 copy number variation in bipolar affective disorder and schizophrenia. *Biol Psychiatry* **71**(10): 922-930.

Maestrini E, Pagnamenta AT, Lamb JA, Bacchelli E, Sykes NH, Sousa I, Toma C, Barnby G, Butler H, Winchester L et al. 2010. High-density SNP association study and copy number variation analysis of the AUTS1 and AUTS5 loci implicate the IMMP2L-DOCK4 gene region in autism susceptibility. *Mol Psychiatry* **15**(9): 954-968.

Mefford HC, Muhle H, Ostertag P, von Spiczak S, Buysse K, Baker C, Franke A, Malafosse A, Genton P, Thomas P et al. 2010. Genome-wide copy number variation in epilepsy: novel susceptibility loci in idiopathic generalized and focal epilepsies. *PLoS Genet* **6**(5): e1000962.

Mikhail FM, Lose EJ, Robin NH, Descartes MD, Rutledge KD, Rutledge SL, Korf BR, Carroll AJ. 2011. Clinically relevant single gene or intragenic deletions encompassing critical neurodevelopmental genes in patients with developmental delay, mental retardation, and/or autism spectrum disorders. *Am J Med Genet A* **155A**(10): 2386-2396.

Mitsui J, Takahashi Y, Goto J, Tomiyama H, Ishikawa S, Yoshino H, Minami N, Smith DI, Lesage S, Aburatani H et al. 2010. Mechanisms of genomic instabilities underlying two common fragile-site-associated loci, PARK2 and DMD, in germ cell and cancer cell lines. *Am J Hum Genet* **87**(1): 75-89.

Mortazavi A, Williams BA, McCue K, Schaeffer L, Wold B. 2008. Mapping and quantifying mammalian transcriptomes by RNA-Seq. *Nat Methods* **5**(7): 621-628.

Nagamani SC, Erez A, Ben-Zeev B, Frydman M, Winter S, Zeller R, El-Khechen D, Escobar L, Stankiewicz P, Patel A et al. 2013. Detection of copy-number variation in AUTS2 gene by targeted exonic array CGH in patients with developmental delay and autistic spectrum disorders. *Eur J Hum Genet* **21**(3): 343-346.

Nalesnik MA, Tseng G, Ding Y, Xiang GS, Zheng ZL, Yu Y, Marsh JW, Michalopoulos GK, Luo JH. 2012. Gene deletions and amplifications in human hepatocellular carcinomas: correlation with hepatocyte growth regulation. *Am J Pathol* **180**(4): 1495-1508.

Nancarrow DJ, Handoko HY, Smithers BM, Gotley DC, Drew PA, Watson DI, Clouston AD, Hayward NK, Whiteman DC. 2008. Genome-wide copy number analysis in esophageal adenocarcinoma using high-density single-nucleotide polymorphism arrays. *Cancer Res* **68**(11): 4163-4172.

Pasic I, Shlien A, Durbin AD, Stavropoulos DJ, Baskin B, Ray PN, Novokmet A, Malkin D. 2010. Recurrent focal copy-number changes and loss of heterozygosity implicate two noncoding RNAs and one tumor suppressor gene at chromosome 3q13.31 in osteosarcoma. *Cancer Res* **70**(1): 160-171.

Paulsen MT, Veloso A, Prasad J, Bedi K, Ljungman EA, Magnuson B, Wilson TE, Ljungman M. 2013a. Use of Bru-Seq and BruChase-Seq for genome-wide assessment of the synthesis and stability of RNA. *Methods*.

Paulsen MT, Veloso A, Prasad J, Bedi K, Ljungman EA, Tsan YC, Chang CW, Tarrier B, Washburn JG, Lyons R et al. 2013b. Coordinated regulation of synthesis and stability of RNA during the acute TNF-induced proinflammatory response. *Proc Natl Acad Sci U S A* **110**(6): 2240-2245.

Perlis RH, Ruderfer D, Hamilton SP, Ernst C. 2012. Copy number variation in subjects with major depressive disorder who attempted suicide. *PLoS One* **7**(9): e46315.

Swaminathan S, Shen L, Kim S, Inlow M, West JD, Faber KM, Foroud T, Mayeux R, Saykin AJ. 2012. Analysis of copy number variation in Alzheimer's disease: the NIALOAD/ NCRAD Family Study. *Curr Alzheimer Res* **9**(7): 801-814.

Tucker T, Zahir FR, Griffith M, Delaney A, Chai D, Tsang E, Lemyre E, Dobrzeniecka S, Marra M, Eydoux P et al. 2013. Single exon-resolution targeted chromosomal microarray analysis of known and candidate intellectual disability genes. *Eur J Hum Genet*.

Vaags AK, Lionel AC, Sato D, Goodenberger M, Stein QP, Curran S, Ogilvie C, Ahn JW, Drmic I, Senman L et al. 2012. Rare deletions at the neurexin 3 locus in autism spectrum disorder. *Am J Hum Genet* **90**(1): 133-141.

Veerappa AM, Saldanha M, Padakannaya P, Ramachandra NB. 2013. Family-based genome-wide copy number scan identifies five new genes of dyslexia involved in dendritic spinal plasticity. *J Hum Genet* **58**(8): 539-547.

Vulto-van Silfhout AT, Hehir-Kwa JY, van Bon BW, Schuurs-Hoeijmakers JH, Meader S, Hellebrekers CJ, Thoonen IJ, de Brouwer AP, Brunner HG, Webber C et al. 2013. Clinical significance of de novo and inherited copy-number variation. *Hum Mutat* **34**(12): 1679-1687.

Wang L, Nuytemans K, Bademci G, Jauregui C, Martin ER, Scott WK, Vance JM, Zuchner S. 2013. High-resolution survey in familial Parkinson disease genes reveals multiple independent copy number variation events in PARK2. *Hum Mutat* **34**(8): 1071-1074.

White SJ, den Dunnen JT. 2006. Copy number variation in the genome; the human DMD gene as an example. *Cytogenet Genome Res* **115**(3-4): 240-246.

Zack TI, Schumacher SE, Carter SL, Cherniack AD, Saksena G, Tabak B, Lawrence MS, Zhang CZ, Wala J, Mermel CH et al. 2013. Pan-cancer patterns of somatic copy number alteration. *Nat Genet* **45**(10): 1134-1140.
